# Structure-Based Design of a Novel Class of Autotaxin Inhibitors Based on Endogenous Allosteric Modulators

**DOI:** 10.1021/acs.jmedchem.2c00368

**Published:** 2022-04-20

**Authors:** Jennifer M. Clark, Fernando Salgado-Polo, Simon J. F. Macdonald, Tim N. Barrett, Anastassis Perrakis, Craig Jamieson

**Affiliations:** † Department of Pure and Applied Chemistry, University of Strathclyde, 295 Cathedral Street, Glasgow G1 1XL, United Kingdom; ‡ Oncode Institute and Division of Biochemistry, Netherlands Cancer Institute, Plesmanlaan 121, 1066CX Amsterdam, The Netherlands; § Medicines Design, GlaxoSmithKline R&D, Stevenage, Hertfordshire SG1 2NY, United Kingdom

## Abstract

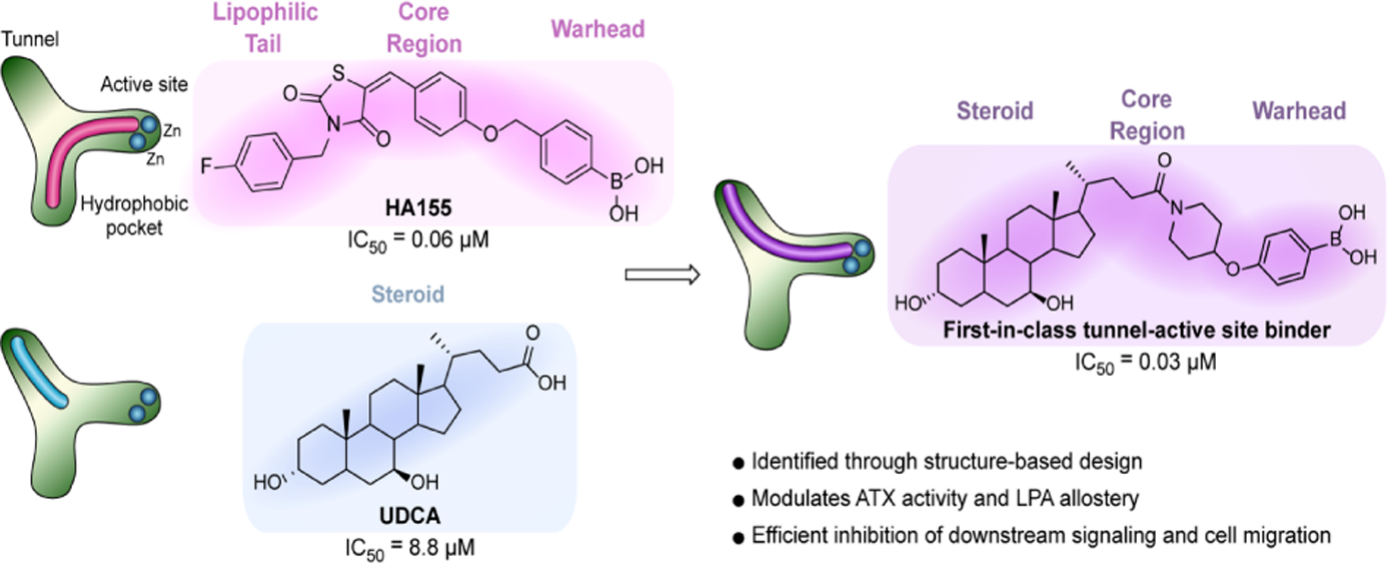

Autotaxin (ATX) facilitates the hydrolysis of lysophosphatidylcholine to lysophosphatidic acid (LPA), a bioactive phospholipid, which facilitates a diverse range of cellular effects in multiple tissue types. Abnormal LPA expression can lead to the progression of diseases such as cancer and fibrosis. Previously, we identified a potent ATX steroid-derived hybrid (partially orthosteric and allosteric) inhibitor which did not form interactions with the catalytic site. Herein, we describe the design, synthesis, and biological evaluation of a focused library of novel steroid-derived analogues targeting the bimetallic catalytic site, representing an entirely unique class of ATX inhibitors of type V designation, which demonstrate significant pathway-relevant biochemical and phenotypic biological effects. The current compounds modulated LPA-mediated ATX allostery and achieved indirect blockage of LPA_1_ internalization, in line with the observed reduction in downstream signaling cascades and chemotaxis induction. These novel type V ATX inhibitors represent a promising tool to inactivate the ATX-LPA signaling axis.

## Introduction

The diversity associated with the ectonucleotide pyrophosphatase/phosphodiesterase (ENPP) family of enzymes has inspired extensive research into their independent pathophysiological functions. Of the seven structurally related enzymes, all of which elicit varying cell signaling responses, autotaxin (ATX, ENPP2) is unique in that it is the only non-membrane bound family member,^1^ therefore representing an attractive and clinically relevant biomarker.

**Figure 1:**
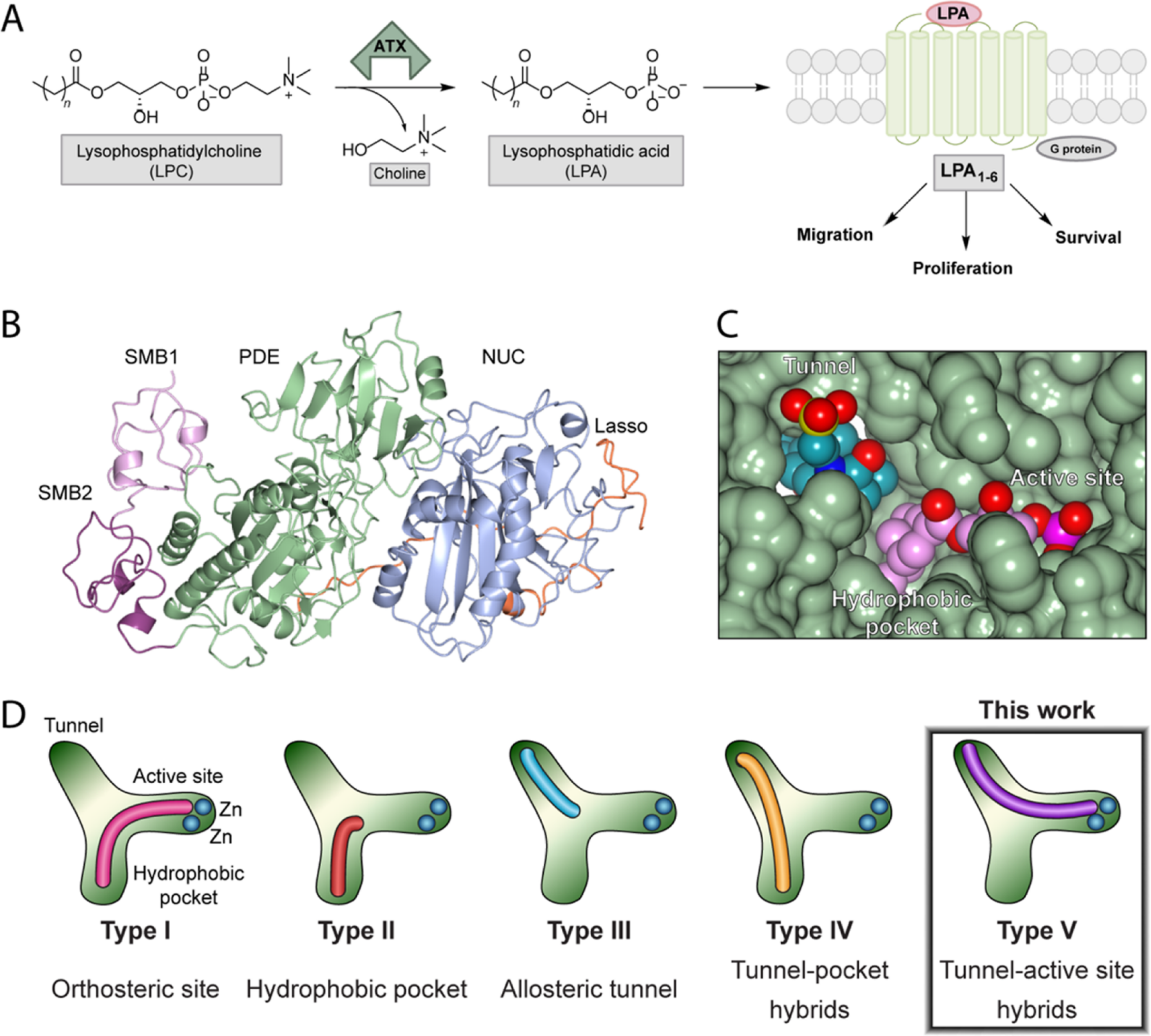
ATX–LPA signaling axis and ATX inhibitor family. (A) Formation of LPA by ATX-mediated hydrolysis of LPC and subsequent receptor recognition and activation. (B) Domain structure of ATX. (C) Surface representation of the ATX tripartite site within the PDE catalytic domain, where 18:1 LPA (pink) and TUDCA (blue) are bound in the orthosteric site and the tunnel, respectively (PDB 5DLW). (D) Classification of the distinct binding modes within the ATX inhibitor family.

First identified in 1992,^2^ ATX was originally defined as an autocrine motility factor. It was later established to be the fundamental mediator responsible for release of the bioactive signaling lipid lysophosphatidic acid (LPA), a family of lysolipids with differing lengths and saturation of their single aliphatic chain, through the cleavage of the corresponding lysophosphatidyl choline (LPC) moiety.^3^ Binding of LPA to its cognate G protein-coupled receptors, LPA_1–6_, initiates its biological activity through receptor activation provoking a cascade of cellular responses, including survival, migration, and proliferation (Figure 1A). The ATX–LPA axis has been implicated in many clinical indispositions, for example, cancer,^4,5^ inflammation,^6^ fibrosis,^7^ autoimmune,^8^ and cardiovascular diseases.^9^ Given its well-defined role in a plethora of pathological and physiological modalities, in addition to its extracellular nature, ATX has been actively pursued over the years as an attractive target for drug discovery in industry and academia alike.^10^

ATX can be subdivided into three main domains: two N-terminal somatomedin β-like (SMB) domains, a phosphodiesterase (PDE) domain and a C-terminal (inactive) nuclease domain (NUC) connected by a lasso loop (Figure 1B).^3,11,12^ The natural LPA substrates bind in the PDE catalytic domain, forming a tripartite binding site: a deep hydrophobic pocket, the bimetallic active site where substrate hydrolysis takes place, and a solvent-accessible hydrophobic tunnel (Figure 1C). The ATX tunnel serves in turn as a secondary LPA binding site, which results in the increase of the catalytic rate of LPC hydrolysis.

A number of ATX inhibitors have been reported in the past decade, which has ultimately led to the classification of four inhibitor types with distinct binding modes in ATX (Figure 1D).^13−18^ A significant fraction of these fall into the category of orthosteric site modulators (type I) as they function to competitively block substrate binding by binding in the active site and hydrophobic pocket. Perhaps two of the most pertinent tool compounds of this nature are 1 (HA155)^14^ and 2 (PF-8380),^13^ both of which are equipped with defined chemotypes which are structurally comparable to those in LPA: a lipophilic tail, a core and linker region, and a distinct warhead. Additionally, hydrophobic pocket binders (type II) compete with substrate binding, without the need of a warhead targeted for the active site, such as CRT0273750^19^ and PAT-494.^18^

Conversely, ATX tunnel binders (type III) owe their modest inhibitory effect to their non-competitive binding mode in the tunnel. In this regard, our extensive structural investigation into the function of the hydrophobic tunnel led to the discovery that sterols, for example, tauroursodeoxycholic acid (TUDCA) and ursodeoxycholic acid (UDCA), are partial non-competitive modulators with micromolar affinity for the ATX tunnel.^20^

Recent progress in inhibitor diversification has led to the evolution of potent hybrid inhibitors (type IV) binding in both the hydrophobic pocket and the hydrophobic tunnel of ATX, such as 3 (GLPG 1690, ziritaxestat),^21^ which reached phase III clinical trials, although it has subsequently been halted in development,^22^ and the steroid derivative 4 developed in our own laboratories.^15^

We have previously reported the structure-driven evolution of potent type IV competitive inhibitors based upon bile salts that act as weak allosteric inhibitors, which facilitated the development of lead compound **4**. Based on the success of 4 in the reduction of LPA levels in vivo, we considered further exploitation of this natural product-derived tunnel-binding skeleton, in combination with an appropriate warhead targeting the active site, which could give rise to a previously unexplored binding mode in terms of ATX inhibition.

In this article, we demonstrate the amalgamation of key structural features from two design hypotheses based on both endogenous allosteric modulators and competitive orthosteric ATX inhibitors, facilitating the development of novel “type V” steroid-derived inhibitors of ATX. A fragment-type biochemical screen identified boron-containing functionalities as suitable warheads, which in conjunction with the steroidal anchor were critical for achieving potency. We then characterized the effect of the novel compound type on ATX kinetics, which provided further confirmation of its binding in the ATX tunnel, hampering both ATX activity and LPA allostery. Cell-based experimental approaches indicated that our compounds robustly modulated ATX activity, yielding in turn a reduction in LPA_1_ internalization and downstream signaling activation, which translated into a less migratory phenotype.

## Results and Discussion

A limited fragment-type screen focused on four warheads for attachment to the steroid manifold, which were predicted to interact with the active site based on their analogy with 1^23^ and structure–activity relationship (SAR)-derived analogues of 2. The activity of these fragments against ATX was determined by measuring LPC hydrolysis in a choline-release biochemical assay (Table 1 and Figure 3), which indicated that boron-containing warheads (6–8) were more favorable candidates than the sulfonamide (5).

**Figure 2:**
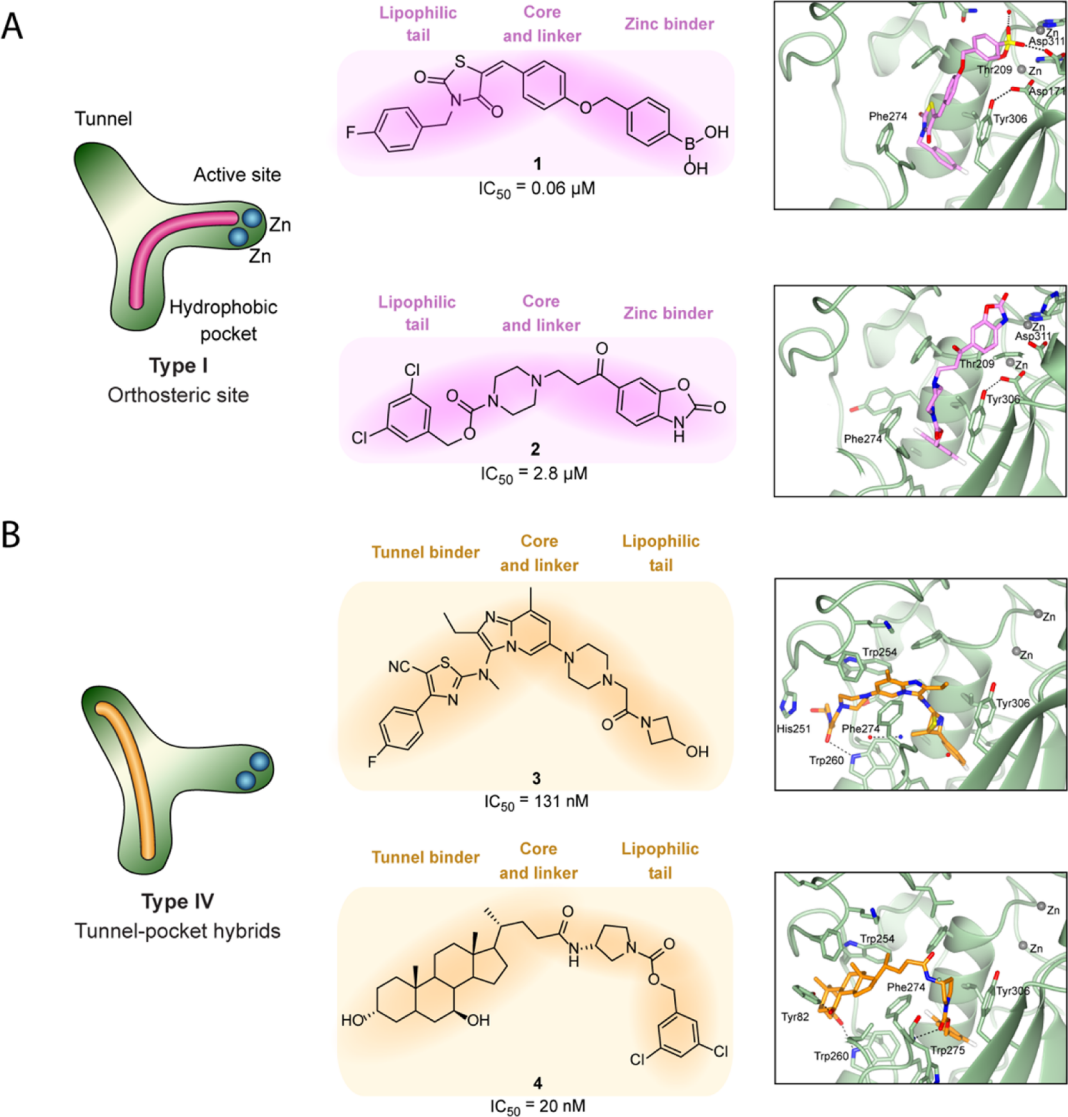
Relevant type I and type IV inhibitors employed for SAR studies. (A) Examples of type I ATX inhibitors which bind orthosterically in the hydrophobic pocket and active site. Structures of 1 (PDB 2XRG) and 2 (PDB 5L0K) are shown with their main interactions. (B) Examples of type IV ATX inhibitors which bind allosterically in the tunnel and hydrophobic pocket. Structures of 3 (PDB 5MHP) and 4 (PDB 5M0M) are shown with their main interactions.

**Figure 3:**
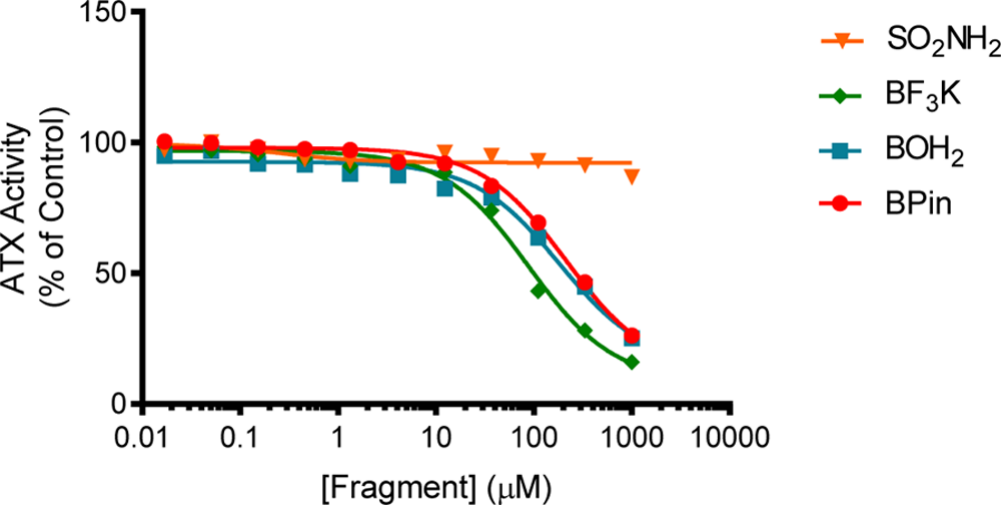
Preliminary screen of fragment-type compounds indicates potential warhead candidates. The choline oxidase-coupled activity assay was used to assess inhibition of LPC hydrolysis by selected fragment compounds **5–8**.

**Table 1:**
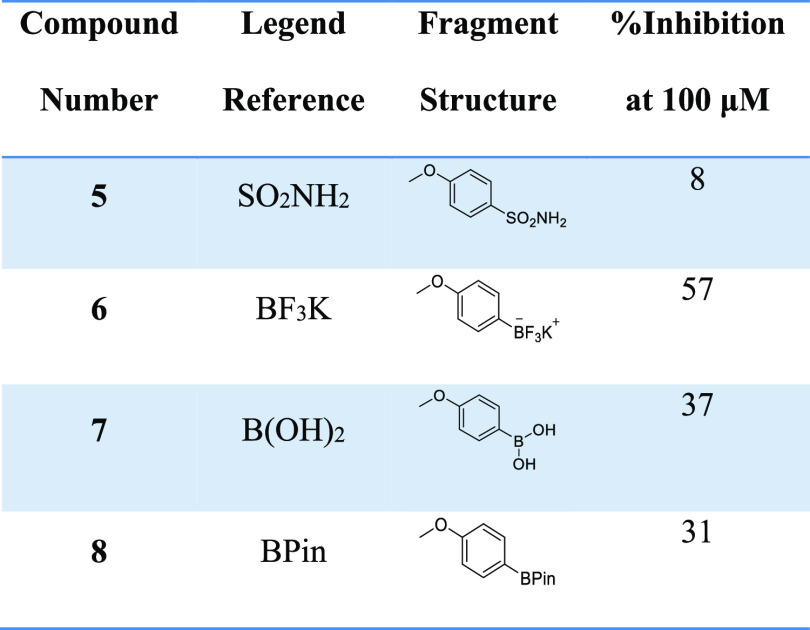
Fragment-Type Biochemical Screen for Selected Compounds **1**–**4**

Due to the novelty associated with the postulated binding mode, our preliminary SAR investigation was focused on probing the length of the spacer required between the warhead and steroid core for optimal biological activity. Despite the lower inhibition of the sulfonamide fragment in the screen, sulfonamides have precedent as active site binders for ATX^10^ and are more synthetically tractable in comparison to the boron-containing warheads. Therefore, the SAR was initially focused on optimizing the compound length with the sulfonamide warhead.

Our initial studies showed that propyl-linked compound **10** with para-sulfonamide substitution on the aryl ring displayed encouraging levels of potency (10, IC_50_ = 0.4 μM) compared to the progenitor steroid (9, IC_50_ = 9 μM) when measured using an LPC hydrolysis assay, shown in Table 2. The activity was lost completely on truncation (11, IC_50_ > 10 μM) and homologation (12, IC_50_ > 10 μM), demonstrating that there was a narrow window in optimal linker length in order to achieve potency. These results indicated that a three-carbon linker was most beneficial to potency.

**Table 2:**
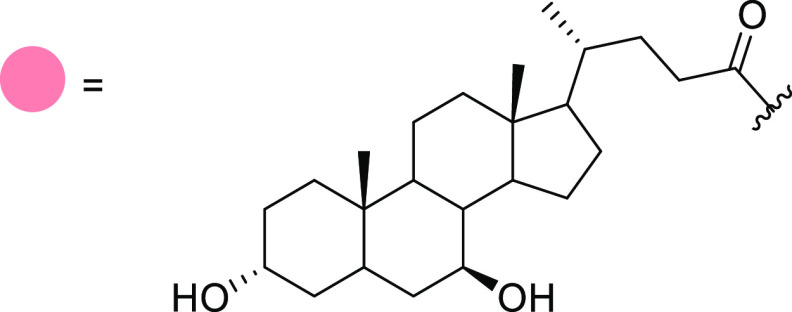

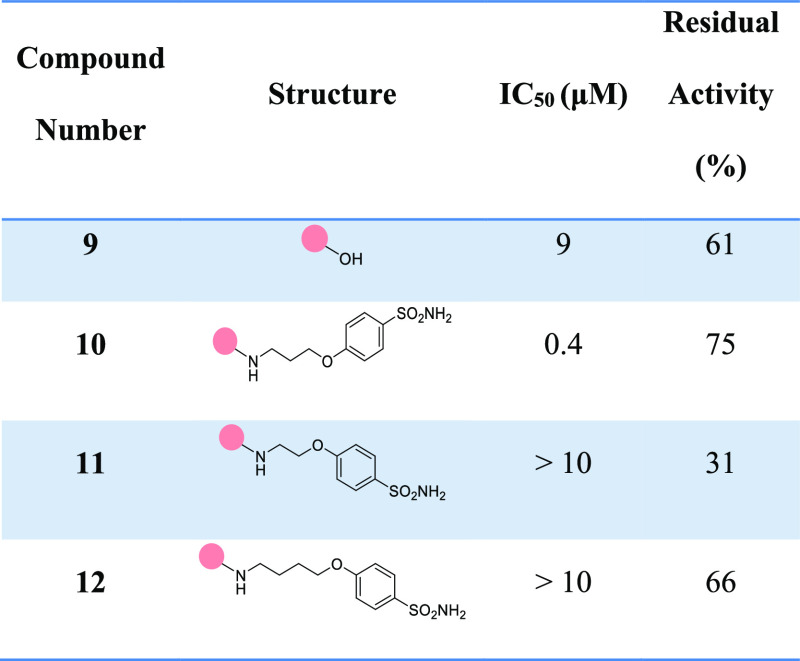
Evaluation of Sulfonamide Analogues

Boron-containing warheads were anticipated to be more active than their sulfonamide counterparts, and a series of matched pairs were generated to explore this hypothesis (Table 3). Although boron-containing compounds are not common features in drug molecules, there are increasing reports of their use in medicinal chemistry campaigns,^24,25^ including for ATX, as evidenced by compound **1** and further more recent analogues.^26^ A 10-fold increase in potency was observed moving from the ethyl-linked meta-substituted pinacol boronate (**13**, IC_50_ = 14 μM) to the para-substituted pinacol boronate (**14**, IC_50_ = 1.5 μM), which corroborated our hypothesis that para-substitution was preferred. This was further substantiated by homologating the linear linker to the propyl derivative, which resulted in a further 10-fold increase in ATX inhibition for meta- (**15**, IC_50_ = 0.15 μM) and para-substituted pinacol boronate (**16**, IC_50_ = 0.07 μM). This indicated that the intrinsic functionality associated with boron-containing motifs plays an important role in inhibitory activity and was better tolerated in combination with an appropriate length of the linker. Pleasingly, analogues bearing a pendant trifluoroborate (**17**, IC_50_ = 0.07 μM) or boronic acid (**18**, IC_50_ = 0.05 μM) retained inhibition of ATX when compared to pinacol boronate **16**. These results reflected the observations generated from the fragment-type biochemical screen, revealing the inherent versatility of all three boron-containing warheads in practice. We believe that partial hydrolysis of the boronate ester occurs under enzymatic assay conditions, which explains the comparable activity between the protected boronic acids (boronates) and the free boronic acid. Control experiments where a phenol is used to replace the boron species showed no enzyme inhibition, confirming the requirement of a warhead for ATX activity. A parameter that was also of importance throughout our SAR campaign, in addition to IC_50_, was the percentage of residual activity for each compound in our biochemical assay. We used this as a tool to aid us in determining the efficacy of each warhead component. As has been demonstrated by us, moving away from the sulfonamide to boron-containing warheads was instrumental in achieving full inhibition of ATX. This further reinforces our initial findings in the fragment screen which highlighted the boronate ester, trifluoroborate, and boronic acid as superior warheads.

**Table 3:**
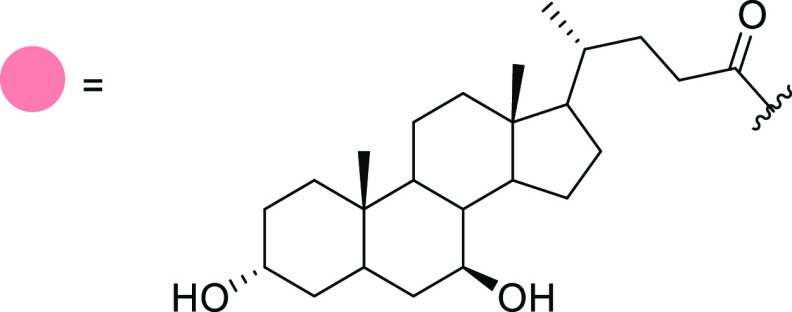

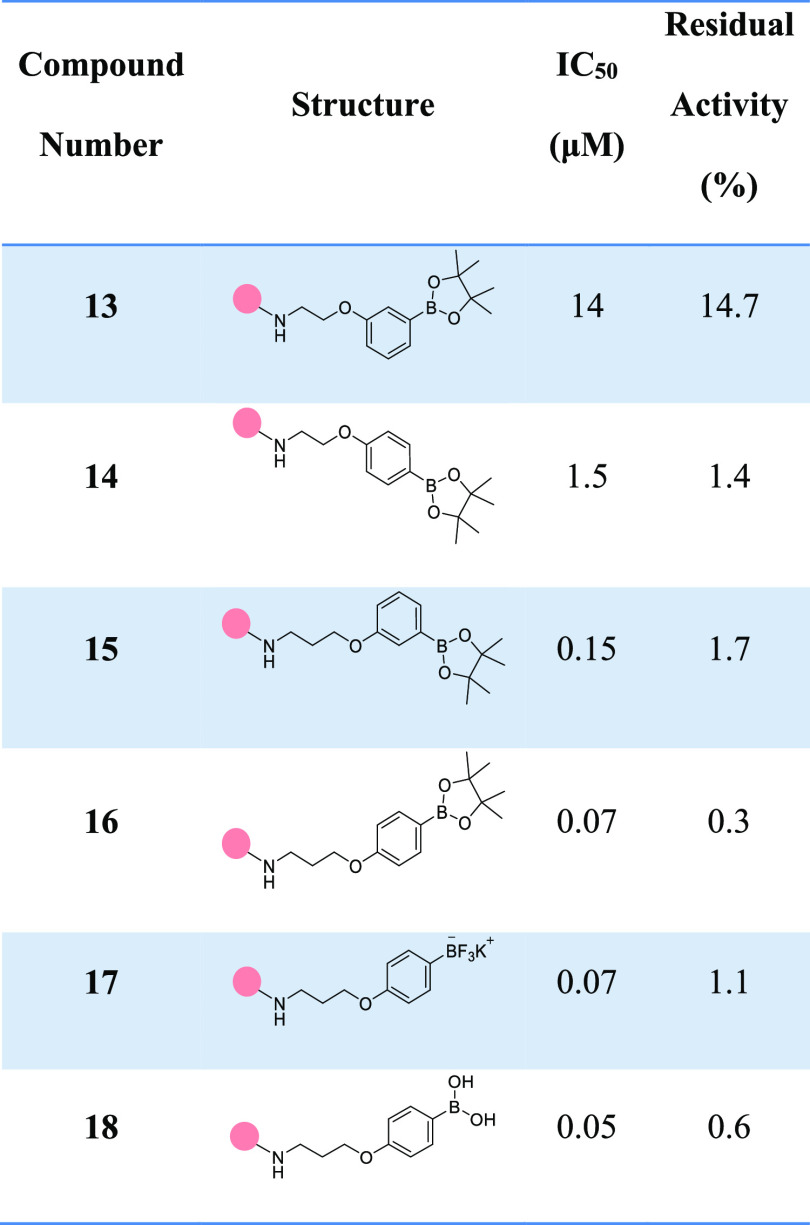
Evaluation of Linear Boron Analogues

The flexibility associated with linear compounds is largely unfavorable based on thermodynamic considerations; however, we reasoned that this could be easily rectified by introducing conformational restraint. With suitable warheads in hand, we next examined compound trajectory by restricting rotational freedom in the linker region while preserving the length. Incorporation of *N*-containing saturated heterocyclic cores (**19–21**, Table 4) combined with the pinacol borane warhead maintained, and piperidine **19** was selected for further optimization. The most significant result was obtained by accessing the corresponding trifluoroborate (**22**, IC_50_ = 0.05 μM) and boronic acid (**23**, IC_50_ = 0.03 μM) derivatives harnessing a piperidine core. Both analogues also demonstrate minimal residual activity, which reflects their efficacy as active site binders. Boronic acid **23** represented a 400-fold increase in activity in comparison to the progenitor steroid (**9**, IC_50_ = 9 μM) and is accompanied by a commensurate improvement in residual activity.

**Table 4:**
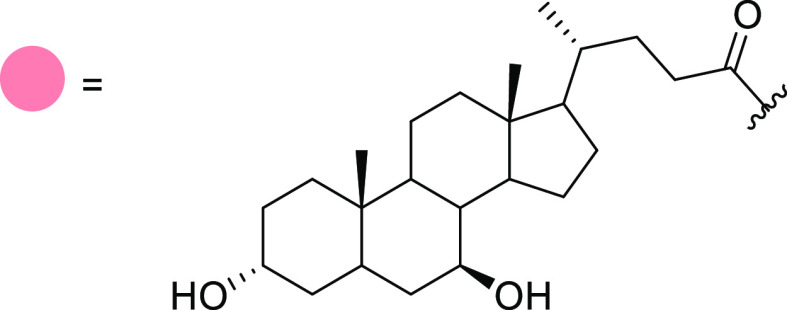

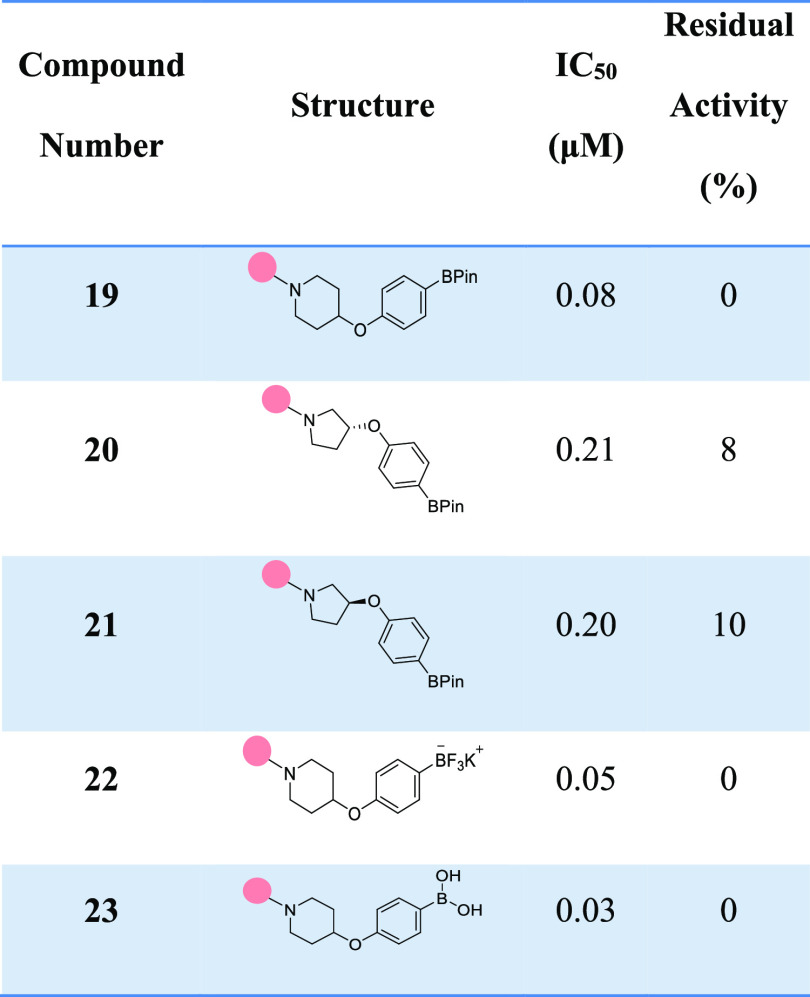
Evaluation of Cyclic Analogues

To gain a comprehensive insight into the binding mode of **22** and **23**, these analogues were co-crystallized with ATX and their crystal structures were determined by X-ray crystallography to 2.5 and 2.1 Å resolutions, respectively.

**Figure 4:**
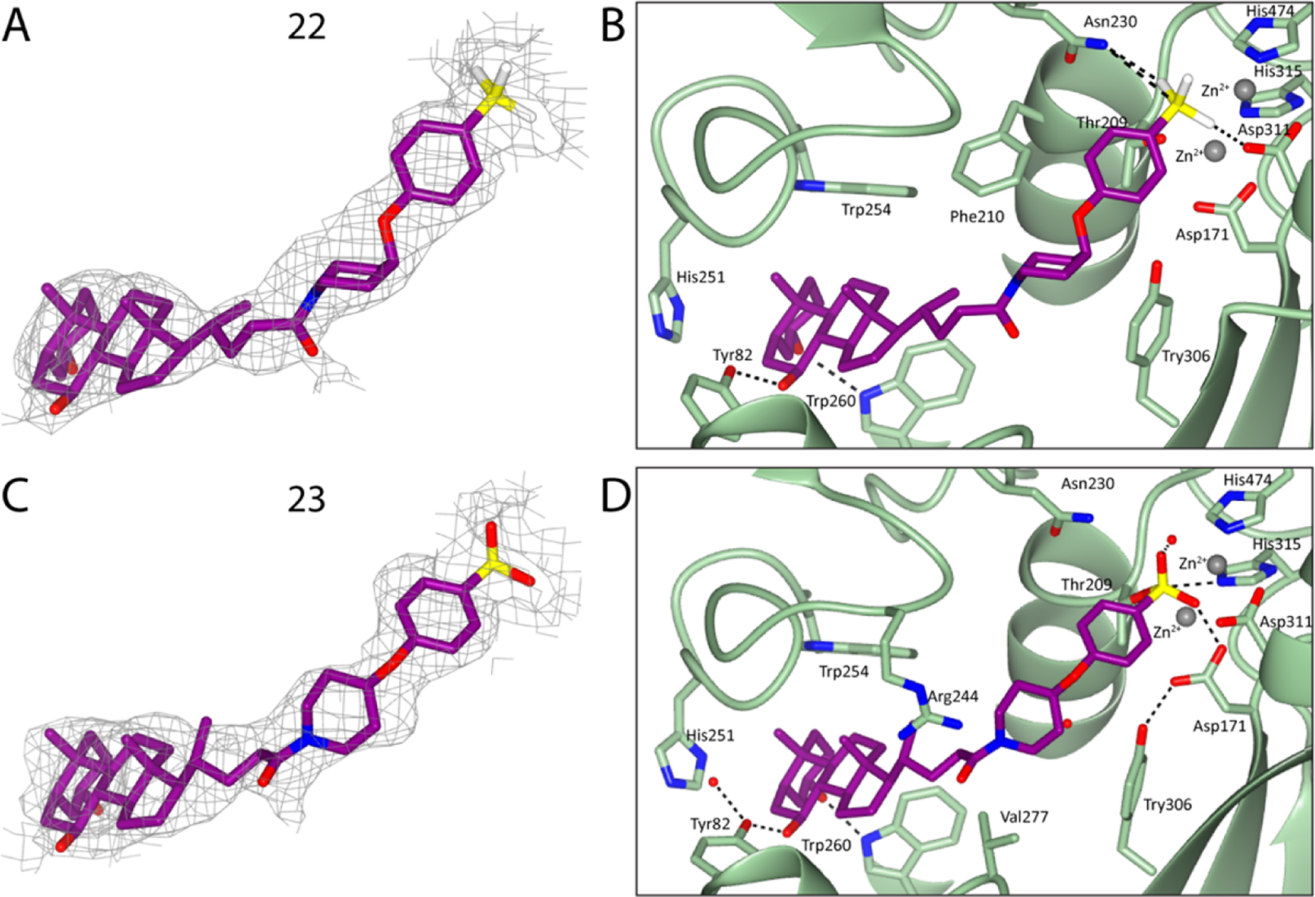
Crystal structures of ATX bound to **22** and **23** confirm their novel binding mode. Fit of **22** (A) and **23** (B) to electron density. Binding modes of **22** (C) and **23** (D) at the ATX tripartite site. Dashed lines indicate hydrogen bonds.

In all structures, the electron density following molecular replacement^27^ and automated refinement^28−30^ clearly confirmed the binding mode of all compounds in the tunnel and the active site (Figure 4A,C). Modeling the compounds and subsequent refinement allowed assignment of a clear, unique conformation for all compounds (Figure 4B,D) and resulted in structures of excellent quality.^31^ Crystallographic and refinement details are presented in Table 5 below.

**Table 5:** Crystallographic Details^a^

data collection	**22** (7Z0 M)	**23** (7Z0 N)
wavelength (Å)	1.0000	1.0000
resolution (Å)	2.00	2.40
space group	*P*12_1_1	*P*12_1_1
unit cell *a b c* (Å), β (deg)	62.3 89.0 76.5, 102.7	62.8 89.6 77.6, 102.8
Data Quality Statistics		
CC_1/2_	0.998 (0.834)	0.982 (0.760)
*R*_merge_	0.043 (0.484)	0.099 (0.621)
⟨*I*/σ*I*⟩	8.3 (1.2)	7.8 (1.7)
completeness (%)	99 (98)	99.9 (100)
redundancy	2.9 (2.9)	3.4 (3.5)
Refinement		
no. of atoms	6599	6877
protein	6241	6412
ligand/metal/glycan	148	186
water/iodine	210	279
TLS groups	1	1
*R*_work_/*R*_free_ (%)	22.6/27.9	19.8/26.3
Validation		
rsmsd/rms*Z* bond lengths (Å)	0.0111/0.718	0.0080/0.517
rsmsd/rms*Z* bond angles (deg)	1.536/0.898	1.484/0.865
Ramachandran preferred/outliers (%)	94.09/0.00	93.89/0.13
Ramachandran *Z* score	–2.30	–2.35
rotamers preferred (%)	91.00	90.26
MolProbity/clash score (percentile)	90/90	90/99

a High-resolution shell in parentheses.

**Figure 5:**
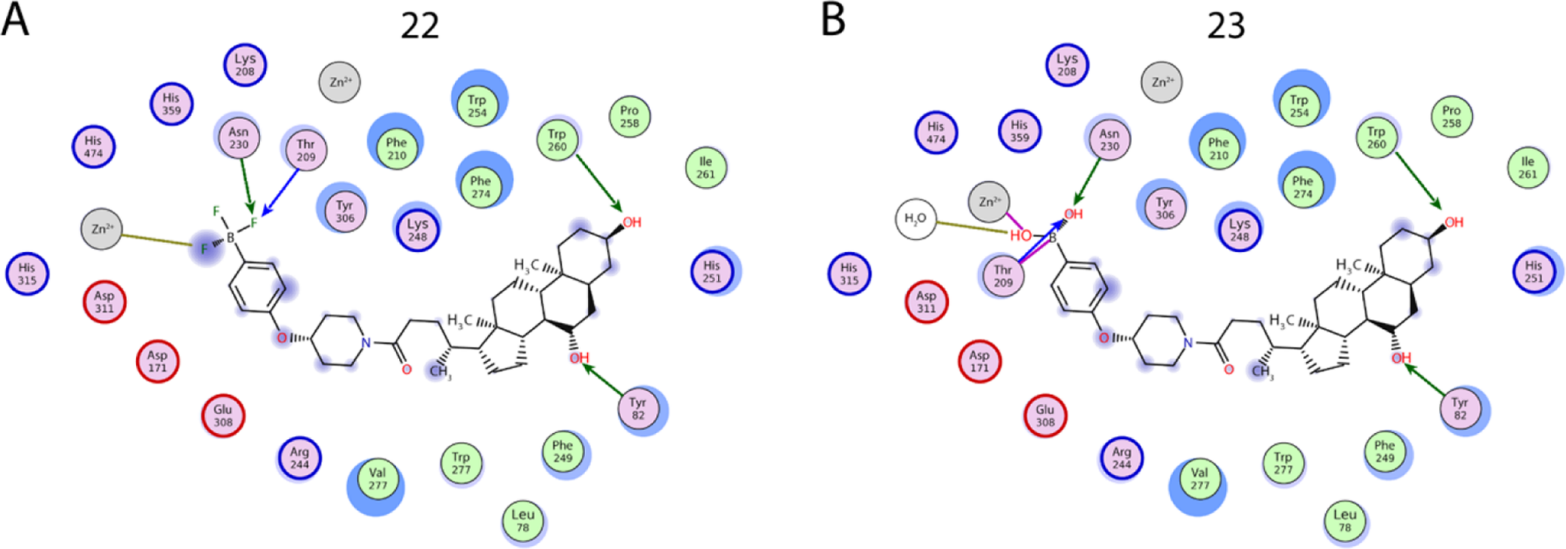
Two-dimensional depiction of the binding modes of the most potent type V compounds. Orientation of **22** (A) and **23** (B) bound at the ATX tunnel and active site. Side- and main-chain hydrogen bonds are indicated in green and blue arrows, respectively, hydrogen bonds with water molecules are represented in gold, coordination to a metal atom is indicated in gray, and the covalent bond of **23** with Thr209 is indicated in purple.

The trifluoroborate warhead of **22** at the active site bore resemblance to the binding mode of the phosphate group of LPA bound at the ATX active site (PDB 5DLW). Specifically, this yielded hydrogen bond interactions with Asn230 and Thr209 via one of the fluorine atoms; a second fluorine was shown to coordinate to one of the proximal zinc ion of the catalytic site (Figure 5A).

Conversely, boronic acid warheads have been well described in the literature based upon the crystallization of HA155 (Figure 2A).^32^ As was expected, the binding pose of **23** at the active site boronic acid warhead entirely resembled that of HA155, which indicates that appropriate orientation was achieved by the length and flexibility of the core region (Figures 4D and 5B). Specifically, the proximity of the boron atom resulted in a reversible covalent bond with the γ-OH group of Thr209. Such reversibility has been previously indicated as a key element in the success of potential ATX inhibitors targeting the active site using boronic acids.^33^ Equally relevant for its binding were the hydrogen bonds formed with Asp171 and Asp311 from one of the hydroxyl groups of the boronic acid, which also coordinated with the proximal zinc ion. The remaining hydroxyl hydrogen-bonded with the main chain of Thr209 as well as a water molecule. In summary, this intricate bond network facilitated the occlusion of the ATX active site and explains the high potency of **23**.

Closer inspection of the steroid moiety revealed similar interactions for both **22** and **23** (Figures 4 and 5). Within the tunnel, they both receive two hydrogen bonds from Trp260 to OH-3 and Tyr82 to OH-5. As expected, the binding mode of the steroid moiety in both compounds fully resembles both the related steroid derivative compound **4** (Figure 2B) and TUDCA (Figure 1C). Consequently, we can hypothesize that the preferred binding mode in the tunnel is where both steroid hydroxyl groups form a hydrogen bond with both Trp260 and Tyr82. Last, a number of hydrophobic interactions are formed by the compounds in the ATX tunnel, specifically with Leu78, Phe210, Leu243, Phe249, Trp254, Trp260, Phe274, and Trp275.

**Figure 6:**
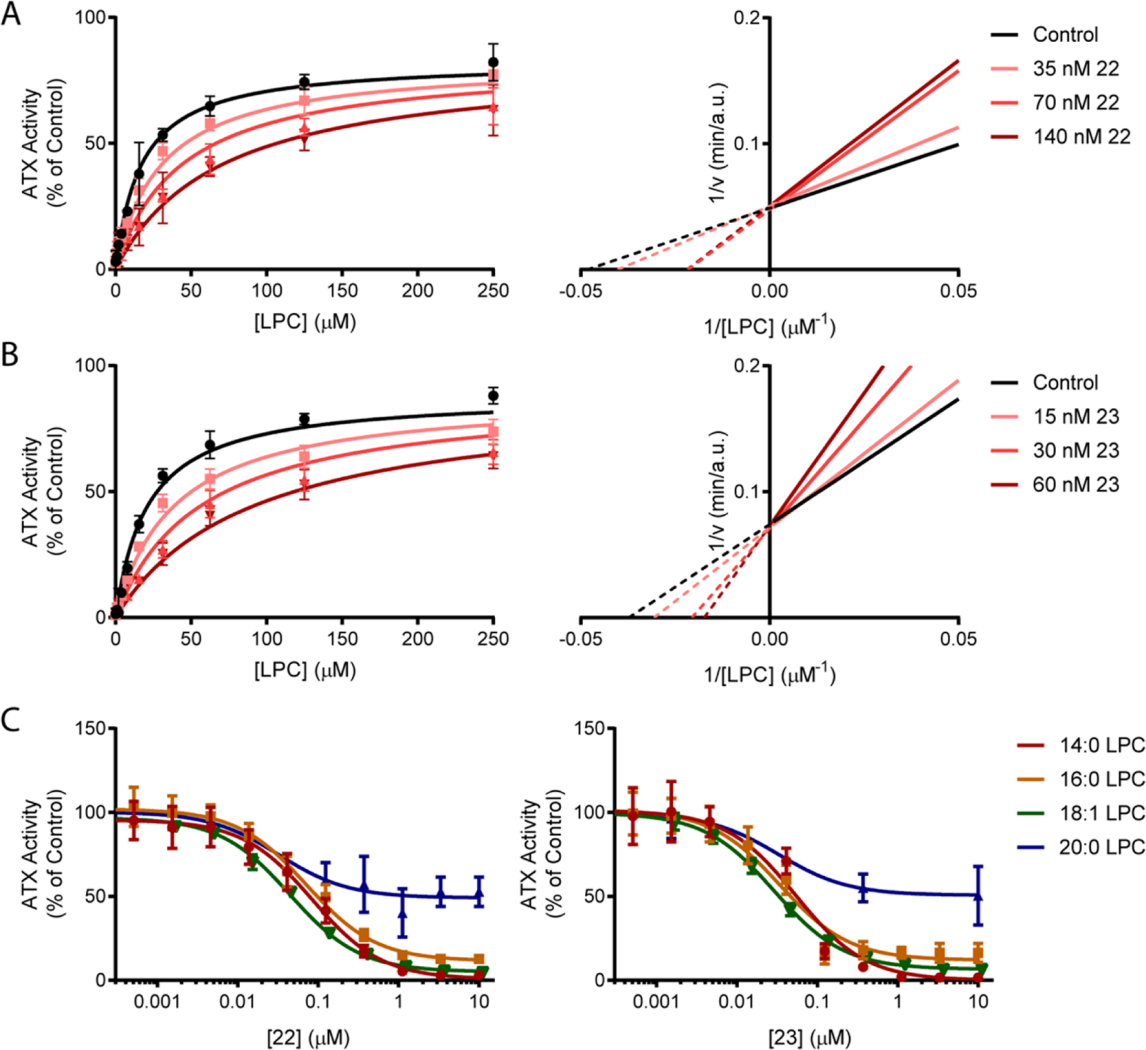
Determination of the mode of inhibition and substrate preference of **22** and **23**. The choline oxidase-coupled activity assay was used to detect inhibition of LPC hydrolysis. (A,B) LPC titration at increasing concentrations of **22** and **23**. In the left panels, nonlinear regression to the Michaelis–Menten equation was employed, from which the inhibition constants were derived, *Ki* = 24 ± 4 nM (A) and 9 ± 1 nM (B). In the right panels, Lineweaver–Burk linear regressions of the same data are provided. Crossing of the ordinate axis at the same 1/*v* value denotes competitive inhibition. The percentage of competitive versus non-competitive inhibition was 99% and α = 9.7 for **22** (A), and 99% and α = 8.5 for **23**. (B) For competitive inhibition. (C) Titration of **22** (left) and **23** (right) for IC_50_ determination of the indicated LPC species. All assays were performed with 150 μM LPC and 20 nM ATX. The mean of three independent experiments ±s.d. is plotted. Statistical analysis of the mode of inhibition was assessed by Akaike’s informative criteria.

Upon defining the structural mode of binding of **22** and **23**, we next wanted to corroborate their potency and mechanism of inhibition (Figure 6A,B). As anticipated, analysis of the inhibition by **22** and **23** confirmed the competitive nature of inhibition over a non-competitive mode (α = χ1, χ1) and showed *Ki* values of 24 ± 4 and 9 ± 1 nM, respectively. Accordingly, the results agreed with the mode of inhibition of the progenitor boronic acid HA155 but contrasts with the progenitor steroid UDCA, which acts as a weak non-competitive inhibitor of LPC hydrolysis. As anticipated previously, the mechanism of action in this emerging lead series has switched by targeting the active site.

To better understand the relevance of substrate preference to the potency of **22** and **23**, we next analyzed their inhibitory activity on the hydrolysis of LPC species with different acyl chain lengths. As noted in Figure 6C and Table 6, both compounds were equally active in the inhibition of 14:0, 16:0, and 18:1 LPC hydrolysis. Strikingly, inhibition of 20:0 LPC hydrolysis only resulted in ∼50% of the catalytic rate of the inhibitor-free control, signifying that both compounds behave as partial inhibitors of longer acyl chains.

**Table 6:** Potency of **22** and **23** in the Inhibition of the Hydrolysis of Different LPC Species

LPC	**22** IC_50_ (μM)	**22** residual activity (%)	**23** IC_50_ (μM)	**23** residual activity (%)
14:0	0.084	1	0.054	0
16:0	0.072	12	0.034	12
18:1	0.045	5	0.029	7
20:0	0.032	49	0.033	51

**Figure 7:**
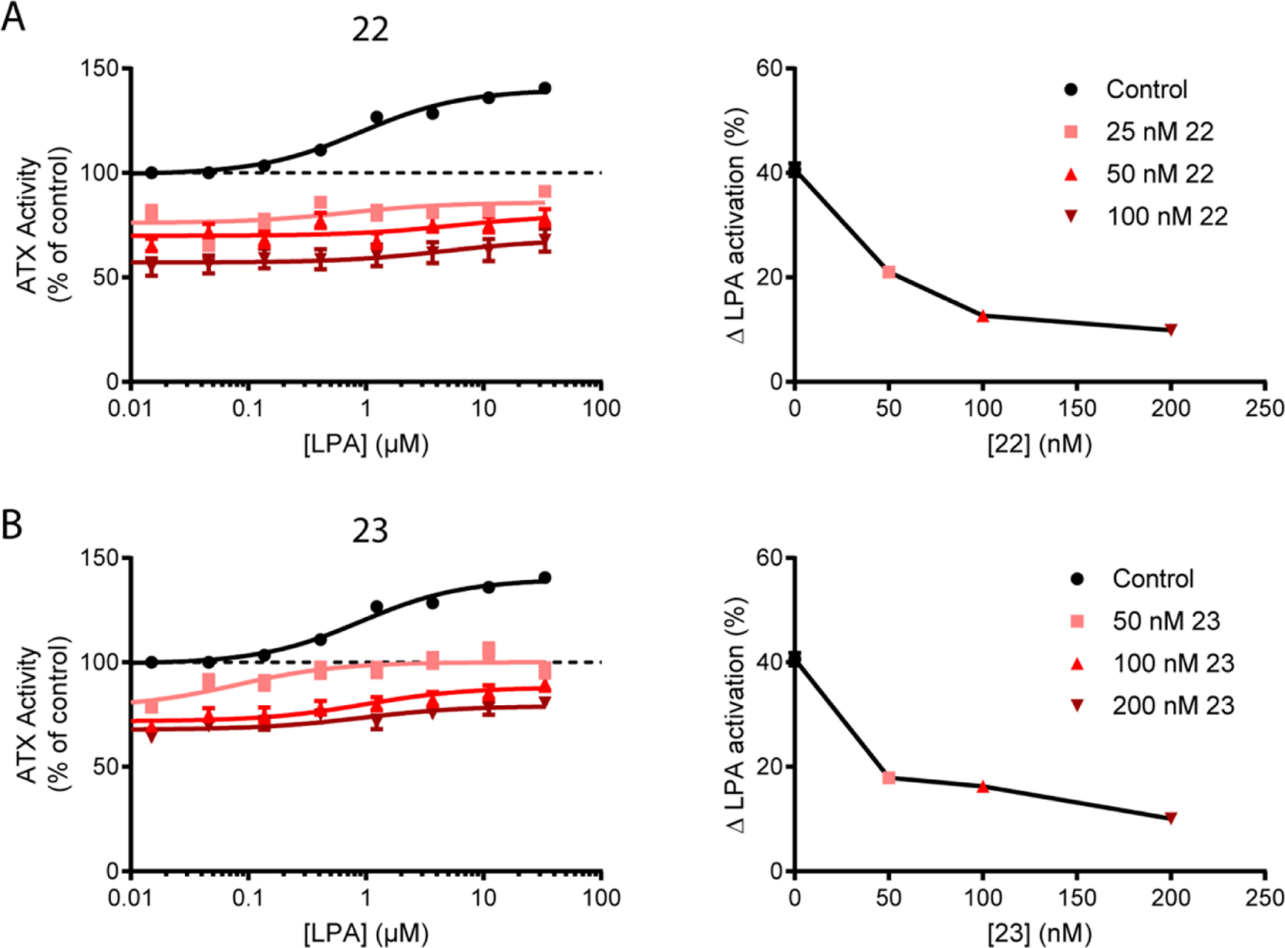
Type V compounds outcompete LPA in the ATX tunnel, abolishing allostery. (A,B) LPA activation in the absence or presence of **22** or **23**. (Left panels) Dose-dependent LPA-related increase in activity of 20 nM ATX pre-incubated with the compounds in a concentration that still allowed residual activity (>50% at the highest inhibitor concentration). (Right panels) Increase of ATX activity with respect to the compound-free control; the data displayed represent the mean value of triplicate measures ± standard error of the mean (SEM).

We have previously described that ATX activity for the hydrolysis of its natural LPC substrates follows a double catalytic cycle.^34^ At low LPA concentrations, ATX hydrolyses LPC at a lower rate, but as the concentration of the LPA product increases and binds to its secondary binding site, the ATX tunnel, the catalytic turnover augments by approximately 40%. This increase in activity can be represented as a function of LPA concentration, which provides an activation constant (AC_50_) of approximately 1 μM. When we examined **22** and **23** in this experimental setup, we observed that addition of an inhibitor at concentrations close to or lower than the IC_50_ value (to allow residual activity) resulted in a dose-dependent decrease of the observed LPA-mediated activation of ATX activity (Figure 7), suggestive of type IV compounds and in sharp contrast to type I compounds that do not ameliorate LPA-mediated activation. This experiment confirms the binding mode and also complements our data on the relevance of competing LPA binding in the tunnel to reduce its subsequent increase in catalytic rate.

**Figure 8:**
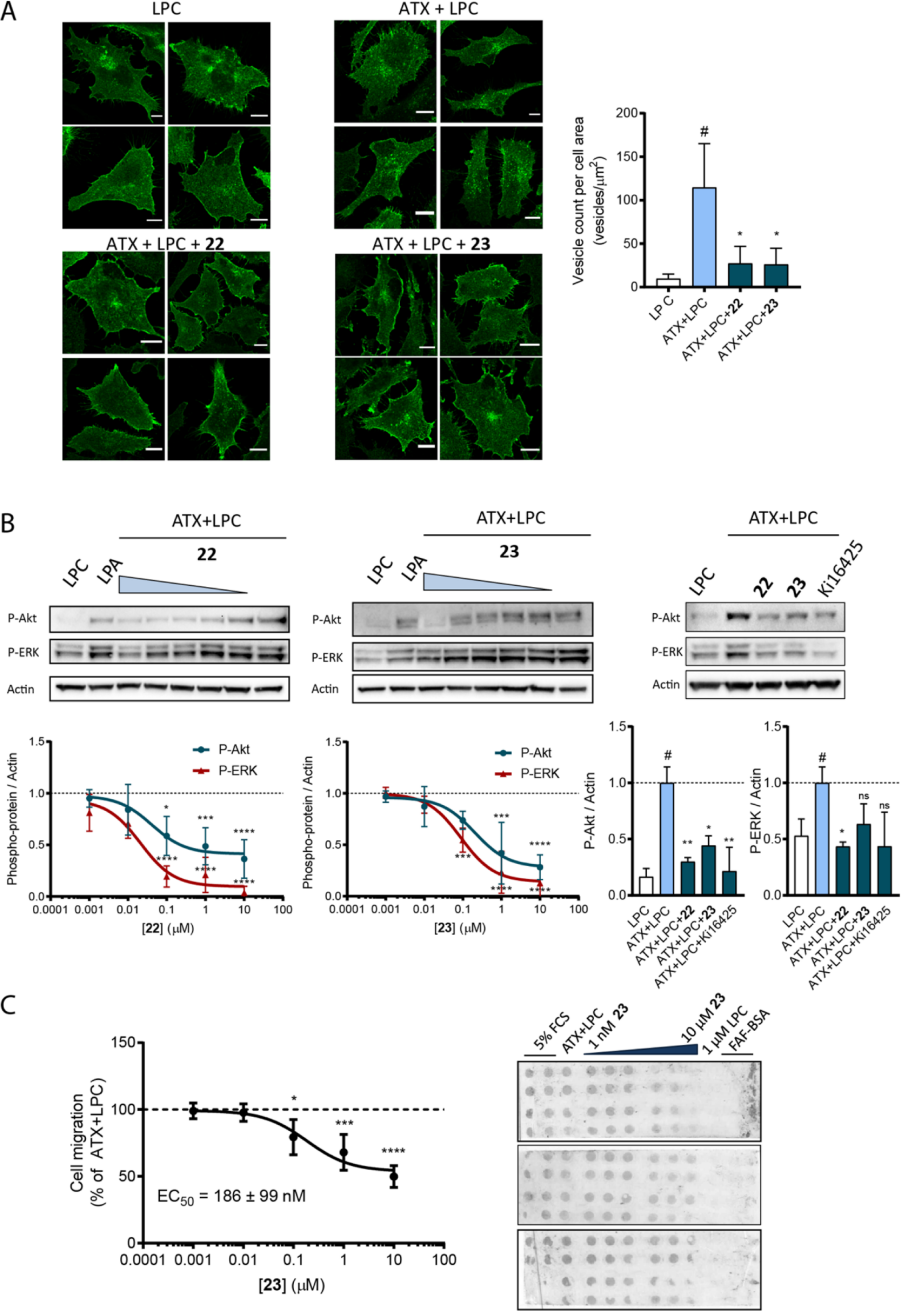
Type V compounds effectively counteract the activation of several hallmarks of the ATX–LPA signaling axis. (A) Left panel, representative confocal images of stained LPA_1_-HA (48 h expression) in HeLa-Flp-In cells, where it localizes mainly to the cell surface or intracellular vesicles; right panel, quantification of LPA_1_ intracellular vesicles. At least 20 cells from three independent preparations were segmented and analyzed by ImageJ to calculate the number of intracellular vesicles (median ± interquartile range); *P < 0.05 (one-way ANOVA). (B) BJeH fibroblasts were challenged with the indicated reagents for 15 min. Representative western blots of three independent experiments are shown, and the mean of the three independent experiments ± s.d. is plotted. (C) Breast cancer MDA-MB-231 cells were allowed to migrate toward the chemoattractant-containing solutions for 4 h. Median ± s.e.m. of the quantitated filters on the right was used for the analysis. For (B,C), the inhibitor concentrations were 10, 1, 0.1, 0.01, and 0.001 μM; for the remaining reagents, the next concentrations were used: 1 μM 18:1 LPC, 1 μM 18:1 LPA, 20 nM ATX, and 10 μM Ki16425.

The biochemical characterization of **22** and **23** helped to validate the novel type V compounds as potent inhibitors of ATX. However, an essential factor in their physiological success requires that they effectively decrease LPA receptor activation and downstream signaling cascades, which in the case of GPCRs can be followed by receptor internalization. For this purpose, we challenged inducible LPA_1_-HA stable HeLa-Flp-In cells with free ATX in the presence of 18:1 LPC and quantified LPA_1_ internalization by confocal imaging. As can be seen in Figure 8A, stimulation of HeLa cells with ATX inhibited by **22** or **23** resulted in a significant decrease of LPA_1_ internalization of approximately 75%. This can be understood as the indirect effect upon blocking LPA production, which hampered receptor activation and endocytosis.

To further corroborate the effect of **22** and **23**, we studied the context of downstream LPA receptor-dependent cellular responses. Among other signals, LPA_1_ activation results in Gα_i_- and Gα_12/13_-driven cascades via PI3K and RhoA activation, respectively. Downstream targets of this signaling pathway are the phosphorylation of the AKT and ERK proteins. We thus utilized human transformed skin fibroblasts (BJeH) which were stimulated with uninhibited or compound-bound ATX, showing that **22** and **23** reduce approximately 60% of the maximal effective cell response compared to positive controls (uninhibited ATX incubated with LPC), as measured by the levels of phosphorylated AKT and ERK (P-AKT and P-ERK), indicated in Figure 8B. The half-maximal effective concentration (EC_50_) that can be assigned to the emerging type V compounds was measured in the same assay and showed EC_50_ values of ∼100 and ∼60 nM for **22** and **23**, respectively, which is broadly consistent with the activity observed in the primary LPC hydrolysis assay.

It is noteworthy that fibroblasts, BJeH cells among these, usually co-express LPA_1_ and LPA_6_ at high levels, which can compound the efforts to decipher receptor-specific cellular responses.^35^ However, given the slight degree of disagreement in the literature as to which Gα subunits couple to LPA_6_, we made use of the nanomolar-affinity LPA_1/2/3_ antagonist Ki16425 to negate the contribution of LPA_6_.^36^ The results revealed that directly antagonizing LPA_1_ amounted to the effects of inhibiting ATX activity by **22** and **23**, which consequently prevents LPA_1_ activation and its Gα_i_- and PI3K-dependent signaling responses.

To further characterize the effect of **23** in efficiently attenuating LPA receptor activation, the chemotaxis induced by Gα_12/13_-driven was put to a test. To this end, we employed the Boyden chamber methodological approach^37^ and quantitated the amount of MDA-MB-231 cells that traversed through a fibronectin-coated filter toward a chemoattractant. As quantitated in Figure 8C, challenging cells with compound-bound or free ATX resulted in a reduction of ∼50% of cell migration, with an EC_50_ value in the range of 200 nM. Since this well-studied cell line mainly expresses LPA_1_, in addition to lower levels of LPA_2/3_, it can be inferred from the data that **23** efficiently diminished LPA production and signaling through these receptors.

## Chemistry

**Scheme 1:**
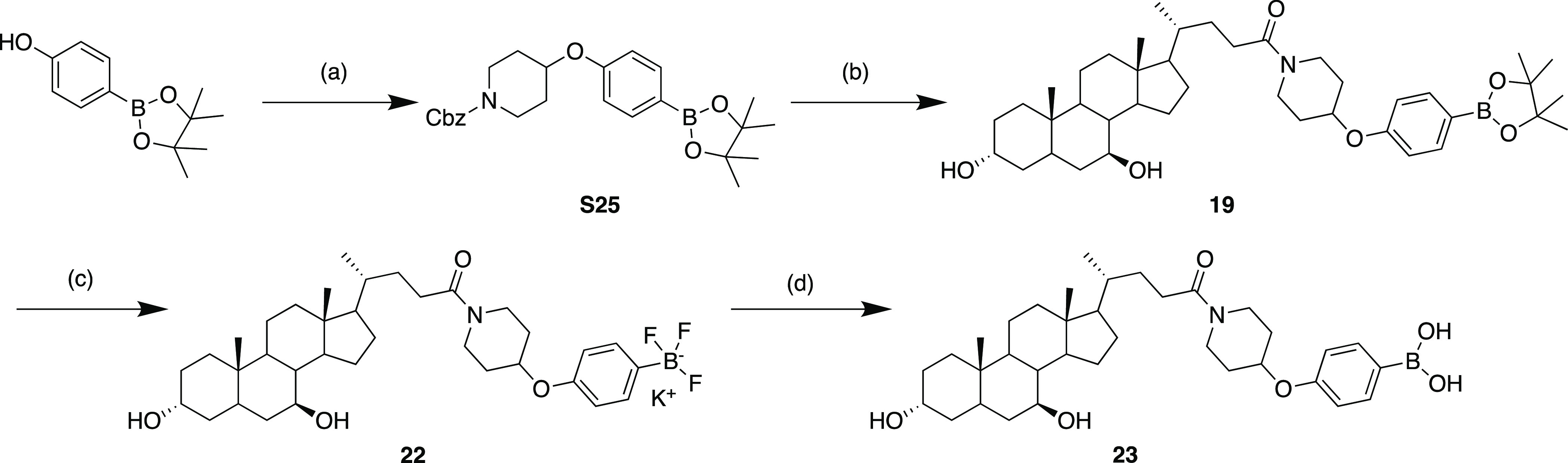
Conditions for cyclic boronates: (a) CMBP, toluene, 120 °C, 67%; (b) pearlman’s catalyst, H_2_, methanol, rt, then UDCA, DIPEA, HATU, DMF, rt, 37% over two steps; (c) KF (aq), *L*-(+)-tartaric acid, acetonitrile/H_2_O (1:1), rt, quant; (d) SiO_2_, H_2_O, rt, 45%.

Pinacol boronate intermediate S25 could be synthesized from the respective phenol via a CMBP-mediated Mitsunobu reaction with the corresponding secondary Cbz-protected amino alcohol in good yield (Scheme 1). Subsequent protecting group removal followed by 1-[bis(dimethylamino)methylene]-1*H*-1,2,3-triazolo[4,5-*b*]pyridinium 3-oxide hexafluorophosphate (HATU)-mediated amine coupling with UDCA led to the generation of pinacol boronate **19**. Compound **19** could be easily converted to the corresponding trifluoroborate **22** in quantitative yield. Sequential SiO_2_ hydrolysis of trifluoroborate **22** provided boronic acid **23** in moderate yield. All other compounds were synthesized in an analogous fashion with full experimental details in the Supporting Information.

## Conclusions

In summary, we successfully report a first-in-class ATX inhibitor of type V designation based on the endogenous steroid modulator, UDCA, which targets the hydrophobic tunnel and active site of the phosphodiesterase domain. Our design approach was constructed through careful consideration and consolidation of previous SAR analysis from both allosteric and orthosteric libraries of ATX compounds, which led to the identification of 23. A 400-fold enhancement in potency compared to the progenitor steroid is demonstrated in addition to a switch in mechanism of inhibition from non-competitive to competitive by tethering an active warhead to the weak endogenous steroid. Our hypothesis is substantiated by crystallographic data of compounds from the lead series bound to ATX. Biochemical and cell-based data for compounds **22** and **23** reveal a competitive mode of inhibition and excellent properties in inhibiting LPA-dependent pathways in cells, as assessed with a panel of relevant assays in different cell lines. These disease-relevant phenotypic studies provide a solid baseline to investigate further the impact of this type of ATX inhibitor in fibroproliferative diseases.

## Experimental Section

### Chemistry—General

All reagents and solvents were obtained from commercial suppliers and were used without further purification unless otherwise stated. Purification was carried out according to standard laboratory methods. Reactions were carried out using conventional glassware. The room temperature was generally 18 °C. Reactions were carried out at elevated temperatures using a temperature-regulated hot plate/stirrer. Thin-layer chromatography was carried out using Merck silica plates coated with fluorescent indicator UV254. These were analyzed under 254 nm UV light or developed using vanillin stain or potassium permanganate solution. Normal-phase flash chromatography was carried out using ZEOprep 60 HYD 40–63 μm silica gel. Fourier-transformed infrared spectra were obtained on a Shimadzu IRAffinity-1 machine. ^1^H and ^13^C NMR spectra were obtained on a Bruker AV 400 at 400 and 101 MHz, respectively, and a Bruker AVIIIHD500 at 500 and 126 MHz, respectively. Chemical shifts are reported in ppm, and coupling constants are reported in Hz with CDCl_3_ referenced at 7.26 (^1^H) and 77.1 ppm (^13^C) and DMSO-*d*_6_ referenced at 2.50 (^1^H) and 39.52 ppm (^13^C). Compound purity was determined by high-performance liquid chromatography and nuclear magnetic resonance (NMR) analysis, and all compounds were of ≥95% purity. High-resolution mass spectra were obtained through analysis at the EPSRC UK National Mass Spectrometry Facility at Swansea University.

### Synthesis of Intermediate S25

To a microwave vial were added 4-hydroxyphenyl boronic acid pinacol ester (300 mg, 1.36 mmol, 1.0 equiv) and benzyl-4-hydroxypiperidine-1-carboxylate (479 mg, 2.04 mmol, 1.5 equiv). The vial was sealed with a microwave cap and purged under N_2_, followed by addition of anhydrous toluene (6 mL). To the vial was added CMBP (715 μL, 2.73 mmol, 2.0 equiv.), and the reaction mixture was heated at 120 °C overnight. The reaction mixture was concentrated in vacuo to a residue which was purified by column chromatography (silica gel, 12% EtOAc in hexane) and then washed with saturated Na_2_CO_3_ (3 × 20 mL) to afford the desired product **S25** as a white solid (400 mg, 67%). ^1^H NMR (CDCl_3_, 400 MHz): 7.74 (d, 2H, *J* = 8.5 Hz), 7.37–7.31 (m, 5H), 6.89 (d, 2H, *J* = 8.5 Hz), 5.14 (s, 2H), 4.58–4.54 (m, 1H), 3.75–3.70 (m, 2H), 3.51–3.46 (m, 2H), 1.92 (brs, 2H), 1.80 (brs, 2H), 1.33 (s, 12H). ^13^C NMR (CDCl_3_, 101 MHz): 159.9, 155.5, 137.0,136.8, 128.7, 128.2, 128.0, 115.3, 83.8, 71.4, 67.3, 40.8, 25.0.2 × C not observed, 1 × C bearing B not observed. ^11^B NMR (CDCl_3_, 126 MHz): 31.9. ν_max_ (neat): 2977, 2941, 2873, 1690, 1603, 1454 cm^–1^. HRMS: exact mass calculated for [M + H]^+^ (C_25_H_33_BNO_5_) requires 438.2456 *m/z*; found, 438.2464 *m*/*z*.

### Synthesis of Compound **19**

To a round-bottomed flask charged with benzyl 4-(4-(4,4,5,5-tetramethyl-1,3,2-dioxaborolan-2-yl)phenoxy)piperidine-1-carboxylate **S25** (300 mg, 0.70 mmol, 1.0 equiv.) were added Pearlman’s catalyst (98 mg, 20 mol %) and methanol (6 mL). The reaction mixture was sparged with H_2_ (balloon) for 1 min and stirred under an atmosphere of H_2_ (balloon) for 5 h. The reaction mixture was filtered through Celite, eluting MeOH. The organics were concentrated in vacuo, and the crude amine intermediate was carried through to the next step without further purification. To a round-bottomed flask was added UDCA (263 mg, 0.67 mmol, 1.0 equiv.) in DMF (5 mL), DIPEA (350 μL, 3.0 mmol, 3.0 equiv), and then HATU (281 mg, 0.74 mmol, 1.1 equiv). After stirring at room temperature for 15 min, the crude amine intermediate (above) was added and stirred for 16 h at room temperature. The reaction mixture was diluted with H_2_O, and the precipitate was filtered by vacuum. The solid was collected and purified by column chromatography (silica gel, 0–5% MeOH in DCM) to afford the desired product as a white solid (167 mg, 37%). ^1^H NMR (CDCl_3_, 400 MHz): 7.75 (d, 2H, *J* = 8.2 Hz), 6.90 (d, 2H, *J* = 8.2 Hz), 4.61–4.60 (m, 1H), 3.74–3.66 (m, 3H), 3.61–3.55 (m, 2H), 3.42–3.40 (m, 1H), 2.42–2.37 (m, 1H), 2.26–2.22 (m, 1H), 2.01–1.99 (m, 1H), 1.96–1.88 (m, 3H), 1.83–1.76 (m 5H), 1.68–1.66 (m, 2H), 1.62–1.51 (m, 5H), 1.48–1.41 (m, 5H), 1.33 (s, 12H), 1.28–1.21 (m, 4H), 1.18–0.99 (m, 4H), 0.96–0.95 (m, 6H), 0.89–0.80 (m, 2H), 0.68 (s, 3H). ^13^C NMR (CDCl_3_, 126 MHz): 172.2, 159.8, 136.8, 115.3, 83.8, 71.6, 71.5, 71.3, 55.9, 55.2, 44.0, 42.6, 42.5, 40.3, 39.3, 38.4, 37.5, 37.0, 35.8, 35.1, 34.2, 31.7, 31.3, 30.6, 30.5, 30.3, 28.9, 27.1, 25.0, 23.5, 21.3, 18.8, 12.3. 1 × C bearing B not observed. ^11^B NMR (CDCl_3_, 160 MHz): 31.4. ν_max_ (neat): 3446, 2928, 2865, 1629, 1606 cm^–1^. HRMS: exact mass calculated for [M + H]^+^ (C_41_H_65_BNO_6_) requires, 678.4912 *m/z*; found, 678.4907 *m*/*z*.

### Synthesis of Compound **22**

To a round-bottomed flask were added **19** (122 mg, 0.18 mmol) and MeOH (0.75 mL), followed by MeCN (0.75 mL). Potassium fluoride (42 mg, 0.71 mmol, 4.0 equiv) in H_2_O was then added, and the reaction mixture was stirred at room temperature until complete dissolution of the boronic ester. L-(+)-tartaric acid (55 mg, 0.36 mmol, 2.05 equiv) was dissolved in tetrahydrofuran and added dropwise to the rapidly stirring biphasic mixture (1000 RPM) over a period of 5 min, and a white precipitate formed. The reaction mixture was stirred for further 2 min before being diluted again with MeCN and filtered. The flask and filter were rinsed with further portions of MeCN, and the combined filtrates were concentrated in vacuo. The resulting solid was washed with Et_2_O (3 × 5 mL) to provide the desired product **22** as a white solid which was isolated as the trifluoroborate salt (118 mg, quant.) without further purification. ^1^H NMR (DMSO-*d*_6_, 400 MHz): 7.22 (d, 2H, *J* = 8.0 Hz), 6.71 (d, 2H, *J* = 7.9 Hz), 4.50–4.47 (m, 1H), 4.43–4.42 (m, 1H), 3.87–3.82 (m, 2H), 3.71–3.68 (m, 1H), 3.23–3.20 (m, 1H), 2.37–2.32 (m, 1H), 2.27–2.18 (m, 1H), 1.96–1.83 (m, 4H), 1.80–1.72 (m, 1H), 1.69–1.65 (m, 3H), 1.59–1.54 (m, 1H), 1.53–1.46 (m, 4H), 1.42–1.30 (m, 7H), 1.25–1.05 (m, 12H), 0.92–0.88 (m, 6H), 0.63 (s, 3H). ^13^C NMR (DMSO-*d*_6_, 126 MHz): 170.9, 154.7, 132.3, 114.3, 71.5, 69.7, 69.4, 55.8, 54.7, 43.1, 43.0, 42.2, 38.7, 38.2, 37.7, 37.3, 35.0, 34.8, 33.7, 31.3, 31.2, 30.5, 30.2, 29.5, 28.2, 26.7, 23.3, 20.8, 18.5, 12.0. 1 × C bearing B not observed. ν_max_ (neat): 3399, 2930, 2865, 1605, 1605, 1454 cm^–1^. ^11^B NMR (DMSO-*d*_6_, 101 MHz): 3.38. ^19^F NMR (DMSO-*d*_6_, 471 MHz): −138.4. HRMS: exact mass calculated for [M – K]^−^ (C_35_H_52_BF_3_NO_4_) requires, 618.3953 *m/z*; found, 618.3953 *m*/*z*.

### Synthesis of Compound **23**

To a round-bottomed flask containing **22** (99 mg, 0.15 mmol) and excess SiO_2_ under N_2_ was added H_2_O (5 mL). The reaction mixture was stirred at room temperature for 1 h before being filtered under vacuum. The filter cake was washed thoroughly with EtOAc, and the filtrate was extracted with H_2_O. The organic phase was separated, and the aqueous phase was extracted twice with EtOAc. The organic phases were combined, washed with brine, dried over MgSO_4_, and concentrated in vacuo. The crude material was then purified by column chromatography (silica gel, 0–12% MeOH in DCM) to afford the desired product **23** as a white solid (45 mg, 50%). ^1^H NMR (DMSO-*d*_6_, 500 MHz): 7.82 (app. br. s., 2H), 7.72 (d, 2H, *J* = 8.3 Hz), 6.92 (d, 2H, *J* = 8.3 Hz), 4.69–4.62 (m, 1H), 4.48–4.42 (m, 1H), 3.87–3.85 (m, 2H), 3.75–3.68 (m, 1H), 3.16–3.32 (m, coincident with solvent), 2.36–2.32 (m, 1H), 2.23–2.18 (m, 1H), 1.95–1.82 (m, 5H), 1.75–1.57 (m, 6H), 1.48–1.42 (m, 4H), 1.41–1.29 (m, 6H), 1.23–1.03 (m, 8H), 0.91–0.87 (m, 6H), 0.62 (s, 3H). ^13^C NMR (DMSO-*d*_6,_ 101 MHz): 170.9, 158.7, 135.9, 114.7, 71.5, 69.7, 69.5, 55.9, 54.7, 43.1, 43.0, 42.2, 38.7, 38.2, 37.7, 37.3, 35.1, 34.8, 33.8, 31.2, 31.1, 30.3, 30.2, 29.5, 28.2, 26.8, 23.3, 20.8, 18.6, 12.0. 1 × C bearing B not observed. ^11^B NMR (MeOD, 128 MHz): 29.7. ν_max_ (neat): 3360, 2928, 2863, 1601 cm^–1^. HRMS: exact mass calculated for [M + ethylene glycol + H]^+^ (C_37_H_57_BNO_6_) requires, 622.4285 *m/z*; found, 622.4288 *m*/*z*.

### Protein Production and Crystallization

Rat ATX was over-expressed and purified as described previously.^38^ For the crystallization studies, ATX was incubated with each screened compound at a 1:10 (protein/compound) ratio for at least 30 min. Crystals were grown for at least 7 days in a 24-well optimization screen (18–20% PEG 3350, 0.1–0.4 M NaSCN, and 0.1–0.4 M NH_4_I). In all cases, the best diffracting crystals were obtained at room temperature (293 K) by mixing 1 μL of the protein/compound solution and 1 μL of the reservoir solution. All crystals were vitrified in a cryoprotectant, which consisted of the reservoir solution with the addition of 20% (v/v) glycerol. The other solvent/protein ratios tested per condition were 1:2, 2:1.

### Crystallographic Data and Methods

The X-ray diffraction data for the ATX–inhibitor complexes with **22** were collected at ESRF on beamline MASSIF1,^39^ and complexes with **23** were collected at SLS on beamline PXIII^40^ at 100 K and were recorded on a PILATUS 2M-F detector to resolutions of 2.00 and 2.40 Å, respectively. All data were processed and integrated with XDS.^40^ All compounds were processed on site using the SLS automated processing pipeline and scaled with AIMLESS.^41^ The structures were determined by molecular replacement using MOLREP^27^ with the structure of ATX (PDB 2XR9) as the search model. Model building and subsequent refinement were performed iteratively with COOT,^28^ REFMAC5,^29^ and PDB_REDO.^30^ Structure validation was carried out by MolProbity.^31^ The structure models and experimental diffraction data were deposited at the PDB under codes 7Z0 M and 7Z0 N for compounds **22** and **23**, respectively. Crystallographic data and refinement details are available in Table 5.

### Biochemical Assays and Modeling of Kinetic Data

The biochemical studies of ATX lysoPLD activity were performed with ATX. Activity was measured by a coupled reaction with 1 U mL^–1^ choline oxidase and 2 U mL^–1^ horseradish peroxidase (HRP) and 2 mM homovanillic acid (HVA) (all from Sigma-Aldrich). For the assays, 14:0, 16:0, 18:1, and 20:0 LPC (Avanti Polar Lipids Inc.) were incubated with 20 nM ATX, reaching a final volume of 100 μL of the buffer, which contained 50 mM Tris, 0.01%, 50 mM CaCl_2_, Triton X-100, pH 7.4. Steady-state choline release was measured at 37 °C by HVA fluorescence at λ_ex_/λ_em_ = 320/460 nm in Corning 96- or 384-well OptiPlate (Sigma-Aldrich) and with a Pherastar plate reader (BMG Labtech). To determine the IC_50_ for the different inhibitors on ATX activity, the velocity of the reaction was monitored for each compound as a function of time and the linear phase of the kinetics was taken from 60 min after the addition of ATX to the reaction buffer. The resulting fluorescence intensity signal over time was used to model all inhibitor concentrations simultaneously using the following formula, where *v*_max_ and *v*_min_ were fitted for the minimum and maximum relative velocities, respectively, and *c*_*i*_ corresponds to the inhibitor concentration for each assay^20^
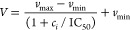
1

### Competition with LPA Allostery

The activation assays using LPA were performed in a similar way to those done for the inhibitors. In this case, LPA was dissolved in ethanol/H_2_0 (1:1) and 0.01% TX-100 and was added to the reaction buffer. The presence of ethanol was taken into account, and controls in the absence of ATX and/or LPC were employed to correct the kinetic data. ATX was incubated for 30 min with different concentrations of inhibitors and subsequently added to the reaction buffer containing 150 μM 18:1 LPC and different starting concentrations of 18:1 LPA. The slopes were calculated from at least 60 min after the addition of ATX. The percentage of LPA-driven activation was normalized to ATX in the absence of LPA and inhibitors, which represented 100% activity. Last, the activation constant or AC_50_ was obtained from the following equation
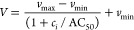
2

### Mechanistic Studies with ATX Inhibitors

For initial comparison between competitive and non-competitive inhibition, we performed assays measuring LPC hydrolysis in the presence of three inhibitor concentrations (0.5 IC_50_, IC_50_, and 2 IC_50_) or with uninhibited ATX, from which slopes taken from 60 min after the start of the reaction were fit in two nonlinear equations^20^
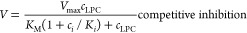
3
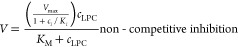
4where *V* is the observed velocity and *c*_LPC_ is the corresponding LPC concentration for each data point, *c*_*i*_ is the inhibitor concentration for each curve, and *K*_*i*_ is the inhibition constant. To statistically determine the chance of each type of inhibition, we calculated the α value in the partial mixed inhibition model (eq 5), where Part defines the partiality of the inhibition, and α > 1 or α = 1 correspond to competitive and non-competitive inhibition modes, respectively. Last, we utilized Akaike’s Information Criterion^42^ to assess the significance of the analysis
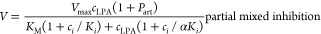
5

### AKT and ERK Phosphorylation by Western Blotting

100,000 BJeH cells were seeded in 12-well tissue culture plates and allowed to grow for 24 h in DMEM (GIBCO, Life Technologies) containing 10% FCS (Thermo Scientific) and 100 U mL^–1^ streptomycin/penicillin (GIBCO, Life Technologies). Next, they were washed twice with phosphate-buffered saline (PBS) and serum-starved O/N. ATX 20 nM was incubated with inhibitors for 30 min in a serum-free medium containing 0.05% (w/v) fatty acid-free BSA (total volume 1 mL). The medium from the 12-well plates was removed and replaced with 1 mL of the ATX–inhibitor mixture. Cells were stimulated for 15 min, the medium was removed, and plates were immediately frozen on dry ice and stored at −80 °C. For western blotting, cells were washed with cold PBS; lysed in RIPA buffer; supplemented with protease inhibitors (Pierce), 20 mM NaF and 1 mM orthovanadate; and spun down. The protein concentration was measured using a BCA protein assay kit (Pierce), and LDS sample buffer (NuPAGE, Invitrogen) and 1 mM dithiothreitol were added to the lysate. 20 μg of the total protein was loaded on sodium dodecyl sulfate–polyacrylamide gel electrophoresis pre-cast gradient gels (4–12% Nu-Page Bis-Tris, Invitrogen), followed by transfer to a nitrocellulose membrane. Non-specific protein binding was blocked by 5% BSA in PBS-Tween (0.1%); the primary antibodies were phospho-Akt (Ser473), catalogue number: D93, dilution: 1:1,000; phospho-ERK1/2 (Thr202/Tyr204), catalogue number: D13.14.4E, dilution: 1:2,000, from Cell Signaling Technology. They were incubated O/N at 4 °C in PBS–Tween with 5% BSA, containing 0.1% NaN_3_. Blots were then incubated for 1 h at room temperature with monoclonal anti-β-actin antibody, clone AC-15, dilution: 1:10,000, from Sigma, which was dissolved in PBS–Tween with 5% skimmed milk containing 0.1% NaN_3_. HRP-conjugated secondary antibodies [goat anti-mouse (Bio-Rad), catalogue number: 1721011; goat anti-rabbit (Pierce), catalogue number: 1858415] were incubated for 1 h at room temperature in PBS–Tween with 2.5% BSA and developed using an ECL Western blot reagent.

### Production of LPA_1_-HA-Expressing HeLa-Flp-In Cells

Human LPA_1_ cDNA was amplified by PCR to remove its stop codon and add the restriction sites for *BamH*I and *Xho*I, after which it was subcloned in an in-house produced pDNA5.1-HA vector. 0.2 μg of the resulting vector and 1.8 μg of pOG44 Flp-Recombinase Expression Vector (Invitrogen) were incubated with 6 μL of Fugene6 (Invitrogen) in 200 μL of OptiMEM (Gibco) for 30 min, after which the mix was added to previously produced HeLa-Flp-In cells.^43^ Their medium was refreshed 24 h later, and selection with 1 μg/mL puromycin was started and maintained with resistant cells.

### Confocal Microscopy for LPA_1_ Internalization

Serum-starved LPA_1_-HA-expressing HeLa-Flp-In cells cultured on 24 mm (#1,5) were treated with 1 μM LPC and 20 nM ATX in the presence or absence of **22** or **23** for 15 min in DMEM containing 0.05% fatty acid-free BSA. Subsequently, coverslips were washed and fixed with 4% PFA, permeabilized with 0.1% Triton X-100, and blocked with 2% BSA for 1 h. Incubation with anti-HA antibody (3F10 from Roche Diagnostics; 1:200) was done for 1 h, followed by incubation with donkey anti-rat Alexa Fluor 488-conjugated antibody (A-21208 from Invitrogen; 1:200) for 1 h at room temperature. For confocal microscopy, cells were washed with PBS, mounted with Immnuno-MountTM (Thermo Scientific), visualized on a LEICA TCS-SP5 confocal microscope (63× objective), and analyzed using ImageJ software.

### Cell Migration Assay

Migration of MDA-MB-231 cells was performed using 48-well chemotaxis chambers (Neuro Probe, Inc.) equipped with 8 mm pore polycarbonate membranes, which were coated with fibronectin (10 μg/mL) (F1141, Sigma-Aldrich). Cells (2 × 10^6^ cells/mL) were added to the upper chamber. 0.05% fatty acid-free BSA was used as a lysophospholipid carrier. Cells were allowed to migrate for 4 h at 37 °C in humidified air containing 5% CO_2_. Migrated cells were fixed in Diff-Quik Fix and stained using Diff-Quik II. Migration was quantified by color intensity measurements using Photoshop software.

## Supporting Information Available

Spectral data for all compounds, preparative details for all compounds, and crystallographic refinement data (PDF)
Molecular formula strings (CSV)

## Accession Codes

The PDB codes for the structures presented are 7Z0M (**22**) and 7Z0N (**23**). The authors will release the atomic coordinates and experimental data upon article publication.

## Abbreviations

AC_50_: half-maximal activation concentration
AKT: protein kinase B
ATX: autotaxin
Cbz: carboxybenzyl
CMBP: cyanomethylenetributylphosphorane
DIPEA: *N,N*-diisopropylethylamine
DMSO: dimethyl sulfoxide
DMF: *N,N*-dimethylformamide
ENPP: ectonucleotide pyrophosphatase/phosphodiesterase
ERK: extracellular signal-regulated kinase
FAF–BSA: fatty acid-free bovine serum albumin
FCS: fetal calf serum
HATU: (1-[bis(dimethylamino)methylene]-1*H*-1,2,3-triazolo[4,5-*b*]pyridinium 3-oxide hexafluorophosphate
HRP: horseradish peroxidase
HVA: homovanillic acid
LPA: lysophosphatidic acid
LPAR: lysophosphatidic acid receptor
LPC: lysophosphatidyl choline
NUC: nuclease
PDB: Protein Data Bank
PDE: phosphodiesterase
PI3K: phosphatidylinositol-3-kinase
SAR: structure–activity relationship
SEM: standard error of the mean
SMB: somatomedin β-like
TUDCA: tauroursodeoxycholic acid
UDCA: ursodeoxycholic acid
