# Supplementary material for: Structure-Based Design of a Novel Class of Autotaxin Inhibitors Based on Endogenous Allosteric Modulators
Source: J Med Chem. 2022 Apr 20;65(8):6338–51. doi: 10.1021/acs.jmedchem.2c00368 (PMC9059126; doi:10.1021/acs.jmedchem.2c00368)
Supplement: Supplementary file 1 — jm2c00368_si_001.pdf [file jm2c00368_si_001.pdf]

Supporting Information

Structure-Based Design of a Novel Class of Autotaxin Inhibitors Based on Endogenous Allosteric Modulators

Jennifer M. Clark,[a] Fernando Salgado-Polo,[b] Simon J. F. Macdonald,[c] Tim N. Barrett,[d] Anastassis Perrakis*[b] and Craig Jamieson*[a]

[a] Department of Pure and Applied Chemistry, University of Strathclyde, 295 Cathedral Street, Glasgow, G1 1XL, United Kingdom

[b] Oncode Institute and Division of Biochemistry, Netherlands Cancer Institute, Plesmanlaan 121, 1066CX, Amsterdam, The Netherlands

[c] Medicines Design, GlaxoSmithKline R&D, Stevenage, Hertfordshire, SG1 2NY, United Kingdom

***craig.jamieson@strath.ac.uk; *a.perrakis@nki.nl**

Contents

1. **Additional Figures** S2
2. **General Procedures** S4
3. **Experimental Details for all intermediates and final compounds** S8
4. **^{1}H NMR, ^{13}C NMR, ^{11}B NMR, ^{19}F NMR and HRMS Spectra** S24
5. **References** S76

1. Additional Figures

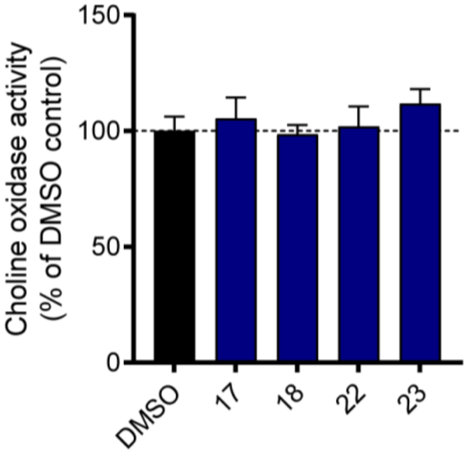

Figure S1. Internal control to assess the potential reactivity of different warheads with the choline oxidase-coupled activity assay. 50 μM choline chloride was incubated with all reaction components in the absence of ATX and LPC.

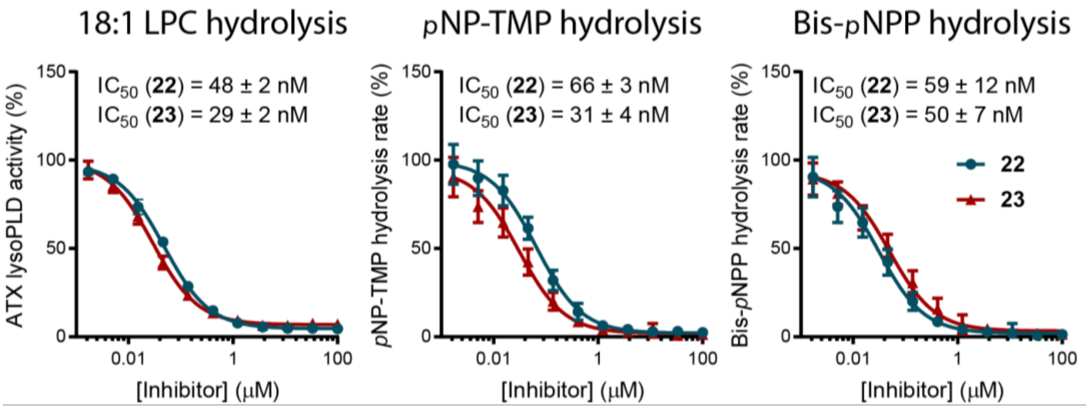

Figure S2. Determination of the potency of **22** and **23** in the hydrolysis of the physiological ATX substrate 18:1 LPC (150 μM), and the synthetic nucleotide substrates *p*NP-TMP (1 mM) and Bis-*p*NPP (1 mM). IC_{50} values are indicated for inhibition in the presence of 20 nM ATX in all cases. The mean of three independent experiments ± s.d. is plotted.

2. General Procedures

General Procedure A – Alkylation

For example, for the preparation of *tert*-butyl (3-(4-sulfamoylphenoxy)propyl)carbamate **S1**

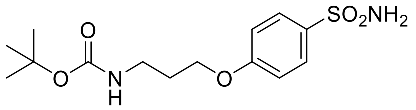

To a round-bottomed flask equipped with a reflux condenser was added 4-hydroxybenzenesulfonamide (200 mg, 1.15 mmol, 1.1 equiv), K_2CO_3 (158 mg, 1.15 mmol, 1.1 equiv) and DMF (5 mL). The reaction mixture was heated to 80 °C and stirred for 20 minutes before addition of *tert*-butyl (3-iodopropyl)carbamate (300 mg, 1.05 mmol, 1.0 equiv). Reaction was stirred overnight at 80 °C. The reaction mixture was diluted with H_2O (80 mL) and the organics were extracted with EtOAc (2 x 30 mL) and washed with brine (80 mL). The organics were dried with a hydrophobic frit and concentrated *in vacuo* and purified by column chromatography on silica (2% MeOH/DCM) to afford the desired product **S1** as a white solid (117 mg, 35%). ^{1}H NMR (DMSO-d_6, 500 MHz): 7.73 (d, *J* = 8.9 Hz, 2H), 7.18 (s, 2H), 7.06 (d, *J* = 8.9 Hz, 2H), 6.90-6.89 (m, 1H), 4.04 (t, *J* = 6.3 Hz, 2H), 3.08 (q, *J* = 6.6 Hz, 2H), 1.84 (quint., *J* = 6.5 Hz, 2H), 1.37 (s, 9H). ^{13}C NMR (DMSO-d_6, 101 MHz): 161.0, 155.6, 136.1, 127.6, 114.4, 77.5, 65.7, 36.8, 29.0, 28.2. ν_{max} (neat): 3381, 3332, 3239, 2930, 1660, 1601, 1630, 1500 cm^{-1}. HRMS: exact mass calculated for $[M+NH_4]^+$ ($C_{14}H_{26}N_3O_5S$) requires 348.1588 *m/z*, found 348.1590 *m/z*. Consistent with previously reported data.[1]

General Procedure B – Amidation

Boc deprotection for the preparation of compounds **10-16**: To a round-bottomed flask was added *tert*-butyl (3-(4-sulfamoylphenoxy)propyl)carbamate **S1** (100 mg, 0.32 mmol) and a mixture of trifluoroacetic acid (230 µL, 3.05 mmol) and DCM (3 mL). The reaction was then

stirred at room temperature until consumption of starting material was confirmed. The reaction was concentrated *in vacuo* yielding a gum which was telescoped through to the subsequent step without further purification.

Cbz deprotection for the preparation of compounds **19-21**: To a round-bottomed flask charged with benzyl 4-(4-(4,4,5,5-tetramethyl-1,3,2-dioxaborolan-2-yl)phenoxy)piperidine-1-carboxylate **S8** (300 mg, 0.80 mmol, 1.0 equiv.) was added Pearlman's catalyst (98 mg, 20 mol %) and methanol (6 mL). The reaction was sparged with H_2 (balloon) for 1 minute, and stirred under an atmosphere of H_2 (balloon) for 5 hours. The reaction was filtered through celite, eluting MeOH. The organics were concentrated *in vacuo* and the crude material was carried through to the next step without further purification.

For compounds **10**-**16** and **19**-**21**, for example, for the preparation of (4*R*)-4-((3*R*,7*S*,10*S*,13*R*)-3,7-dihydroxy-10,13-dimethylhexadecahydro-1*H*-cyclopenta[*a*]phenanthren-17-yl)-*N*-(3-(4-sulfamoylphenoxy)propyl)pentanamide **10**

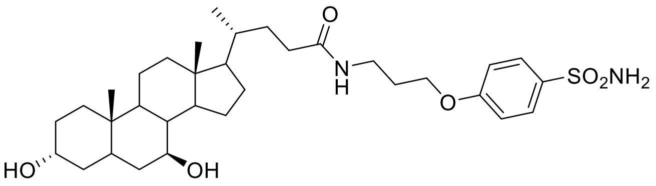

tert-butyl (3-(4-sulfamoylphenoxy)propyl)carbamate **S1** (100 mg, 0.44 mmol, 1.0 equiv.) was subjected to Boc deprotection conditions outlined above. To a round-bottomed flask was added ursodeoxycholic acid (UDCA, 85 mg, 0.22 mmol, 1.0 equiv) in dimethylformamide (DMF, 2 mL), *N,N*-diisopropylethylamine (DIPEA, 237 µL, 1.36 mmol, 5.0 equiv) and then 1-[bis(dimethylamino)methylene]-1*H*-1,2,3-triazolo[4,5-*b*]pyridinium 3-oxide hexafluorophosphate (HATU, 90 mg, 0.24 mmol, 1.1 equiv). After stirring at room temperature for 15 minutes, crude intermediate 2-(4-sulfamoylphenoxy)ethan-1-aminium 2,2,2-trifluoroacetate **S1a** was added and stirred for 16 hours at room temperature. The reaction

mixture was diluted with H_2O (30 mL) and the organics were extracted with EtOAc (2 x 20 mL) and washed with brine (80 mL). The organics were dried with a hydrophobic frit and concentrated *in vacuo* to a residue that was purified by column chromatography on silica (10% MeOH/DCM) to afford desired product **10** as an off-white solid (56 mg, 30% over two steps). ^{1}H NMR (DMSO-d_6, 500 MHz): 7.84 (t, J = 5.5 Hz, 1H), 7.74 (d, J = 8.8 Hz, 2H), 7.18 (s, 2H), 7.06 (d, J = 8.8 Hz, 2H), 4.42 (d, J = 4.5 Hz, 1H), 4.05 (t, J = 6.2 Hz, 2H), 3.85 (d, J = 6.8 Hz, 1H), 3.33-3.24 (m, coincident with solvent peak), 3.20-3.16 (m, 2H), 2.11-2.06 (m, 1H), 1.99-1.91 (m, 2H), 1.87-1.81 (m, 3H), 1.76-1.72 (m, 1H), 1.68-1.63 (m, 3H), 1.49-1.26 (m, 11H), 1.22-0.91 (m, 9H), 0.89-0.87 (m, 6H), 0.60 (s, 3H). ^{13}C NMR (DMSO-d_6, 101 MHz): 172.6, 160.9, 136.1, 127.6, 114.4, 69.7, 69.5, 65.7, 55.9, 54.7, 43.1, 43.0, 42.2, 38.7, 37.7, 37.3, 35.3, 34.9, 34.8, 33.7, 32.4, 31.7, 30.2, 28.8, 28.2, 26.7, 23.3, 20.8, 18.5, 12.0. 1 x C not observed. ν_{max} (neat): 3381, 2929, 2864, 1633, 1597, 1547 cm^{-1}. HRMS: exact mass calculated for [M-H]$^-$ ($C_{33}H_{51}N_2O_6S$) requires 603.3473 *m/z*, found 603.3473 *m/z*.

General Procedure C – CMBP-Mediated Mitsunobu

For example, for the preparation of *tert*-butyl (2-(3-(4,4,5,5-tetramethyl-1,3,2-dioxaborolan-2-yl)phenoxy)ethyl)carbamate **S4**

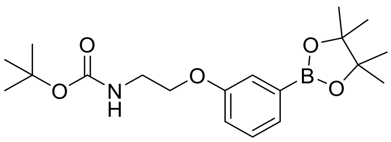

To a microwave vial was added 3-hydroxyphenyl boronic acid pinacol ester (200 mg, 0.90 mmol, 1.0 equiv.) and *N*-Boc ethanolamine (210 µL, 1.36 mmol, 1.3 equiv.). The vial was sealed with a microwave cap, purged under N_2 followed by addition of anhydrous toluene (4 mL). To the vial was added CMBP (471 µL, 1.09 mmol, 2.0 equiv.) and the reaction heated at 120 °C overnight. Reaction mixture was concentrated *in vacuo* to a residue that was purified

by column chromatography on silica (7% EtOAc in hexane) then washed with saturated Na_2CO_3 (3 x 20 mL) to afford the desired product **S4** as a clear oil (183 mg, 56%). ^{1}H NMR ($CDCl_3$, 500 MHz): 7.42 (d, *J* = 7.2 Hz, 1H), 7.32-7.28 (m, 2H), 7.01-6.98 (m, 1H), 4.98 (br. s, 1H), 4.05 (t, *J* = 5.0 Hz, 2H), 3.54-3.53 (m, 2H), 1.45 (s, 9H), 1.34 (s, 12H). ^{13}C NMR ($CDCl_3$, 126 MHz): 159.3, 158.2, 129.2, 127.7, 120.0, 118.2, 84.0, 79.4, 67.3, 40.3, 28.6, 25.0. 1 x C bearing B not observed. ^{11}B NMR ($CDCl_3$, 160 MHz): 30.9. ν_{max} (neat): 3370, 2975, 2930, 1687, 1536 cm^{-1}. HRMS: exact mass calculated for $[M+H]^+$ ($C_{19}H_{31}BNO_5$) requires 364.2290 *m/z*, found 364.2289 *m/z.*

3. Experimental Details for intermediates and final compounds

Preparation of *tert*-butyl (2-(4-sulfamoylphenoxy)ethyl)carbamate **S2**

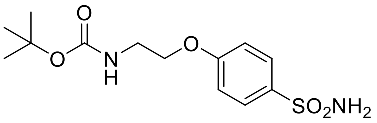

Prepared according to General Procedure A, using 4-hydroxybenzenesulfonamide (200 mg, 1.15 mmol, 1.1 equiv.), *tert*-butyl (2-bromoethyl)carbamate (235 mg, 1.04 mmol, 1.0 equiv.) K_2CO_3 (158 mg, 1.15 mmol, 1.1 equiv.) and DMF (5 mL). The crude material was subjected to the purification outlined in General Procedure A (silica gel, 2% MeOH/DCM) to afford desired product **S2** as a white solid (117 mg, 35%). ^{1}H NMR (DMSO-d_6, 500 MHz): 7.73 (d, J = 8.9 Hz, 2H), 7.19 (s, 2H), 7.07 (d, J = 8.9 Hz, 2H), 7.02 (t, J = 5.5 Hz, 1H), 4.03 (t, J = 5.5 Hz, 2H), 3.33-3.28 (m, coincident with solvent), 1.38 (s, 9H). ^{13}C NMR (DMSO-d_6, 126 MHz): 160.8, 155.7, 136.3, 127.6, 114.5, 77.8, 66.8, 28.2. 1 x C not observed. ν_{max} (neat): 3398, 3317, 3218, 3112, 2969, 1667, 1597, 1521, 1597, 1521, 1502 cm^{-1}. HRMS: exact mass calculated for $[M+Na]^+$ ($C_{13}H_{20}N_2O_5SNa$) requires 339.0985 *m/z*, found 339.0989 *m/z*.

Preparation of *tert*-butyl (4-(4-sulfamoylphenoxy)butyl)carbamate **S3**

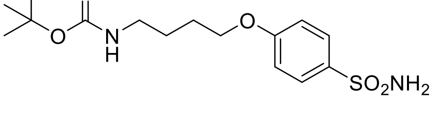

Prepared according to General Procedure A, using 4-hydroxybenzenesulfonamide (191 mg, 1.10 mmol, 1.1 equiv.), *tert*-butyl (4-iodobutyl)carbamate (300 mg, 1.00 mmol, 1.0 equiv.), K_2CO_3 (152 mg, 1.10 mmol 1.1 equiv.) and DMF (5 mL). The crude material was subjected to the purification outlined in General Procedure A (silica gel, 60% EtOAc in petroleum ether 40-60) to afford the desired product **S3** as an off-white solid (148 mg, 39%). ^{1}H NMR (DMSO-d_6, 500 MHz): 7.73 (d, J = 8.9 Hz, 2H), 7.18 (s, 2H), 7.06 (d, J = 8.9 Hz, 2H), 6.85-6.83 (m, 1H),

4.04 (t, J = 6.5 Hz, 2H), 2.97 (app. q, J = 6.6 Hz, 2H), 1.74-1.68 (m, 2H), 1.55-1.49 (m, 2H), 1.37 (s, 9H). ^{13}C NMR (DMSO-d_6, 101 MHz): 161.0, 155.6, 136.0, 127.6, 114.4, 77.4, 67.6, 28.2, 26.0, 25.9. 1 x C not observed. ν_{max} (neat) 3363, 3281, 2952, 2926, 2865, 1682, 1599, 1526 cm^{-1}. HRMS: exact mass calculated for $[M+Na]^+$ ($C_{15}H_{24}N_2O_5SNa$) requires 367.1298 *m/z*, found 367.1299 *m/z*.

Preparation of *tert*-butyl (2-(4-(4,4,5,5-tetramethyl-1,3,2-dioxaborolan-2-yl)phenoxy)ethyl)carbamate **S5**

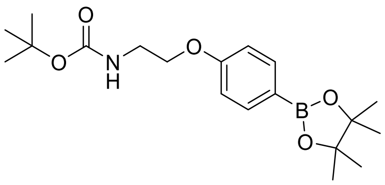

Prepared according to General Procedure C, using 4-hydroxyphenyl boronic acid pinacol ester (200 mg, 0.90 mmol, 1.0 equiv.), *N*-Boc ethanolamine (210 μL, 1.36 mmol, 1.5 equiv.), CMBP (471 μL, 1.09 mmol , 2.0 equiv.) in anhydrous toluene (4 mL). The crude material was subjected to the purification outlined in General Procedure C (silica gel, 0-10% EtOAc in hexanes) then washed with saturated Na_2CO_3 (3 x 20 mL) to afford the desired product **S5** as a clear oil (250 mg, 77%). ^{1}H NMR ($CDCl_3$, 500 MHz): 7.74 (d, J = 8.4 Hz, 2H), 6.88 (d, J = 8.4 Hz, 2H), 4.97 (br. s, 1H), 4.04 (t, J = 5.0 Hz, 2H), 3.54-3.53 (m, 2H), 1.45 (s, 9H), 1.33 (s, 12H). ^{13}C NMR ($CDCl_3$, 126 MHz): 161.3, 156.0, 136.7, 114.0, 83.7, 79.7, 67.2, 40.3, 28.5, 25.0. 1 x C bearing B not observed. ^{11}B NMR ($CDCl_3$, 160 MHz): 30.9. ν_{max} (neat): 3360, 2977, 2932, 1700, 1606, 1513 cm^{-1}. HRMS: exact mass calculated for $[M+H]^+$ ($C_{19}H_{31}BNO_5$) requires 364.2290 *m/z*, found 364.2285 *m/z*.

Preparation of *tert*-butyl (3-(3-(4,4,5,5-tetramethyl-1,3,2-dioxaborolan-2-yl)phenoxy)propyl)carbamate **S6**

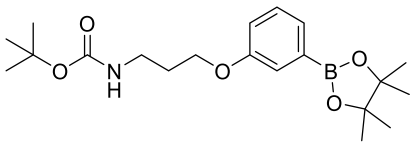

Prepared according to General Procedure C, using 3-hydroxyphenyl boronic acid pinacol ester (200 mg, 0.90 mmol, 1.0 equiv.), *tert*-butyl (3-hydroxypropyl)carbamate (238 mg, 1.36 mmol, 1.5 equiv.), CMBP (471 μL, 1.09 mmol , 2.0 equiv.) in anhydrous toluene (4 mL). The crude material was subjected to the purification outlined in General Procedure C (silica gel, 0-7% EtOAc in hexane) then washed with saturated Na_2CO_3 (3 x 20 mL) to afford the desired product **S6** as a clear oil (298 mg, 88%). ^{1}H NMR ($CDCl_3$, 500 MHz): 7.40-7.39 (m, 1H), 7.32-7.31 (m, 1H), 7.28 (m, coincident with solvent peak), 7.00-6.98 (m, 1H), 4.77 (br. s., 1H), 4.05 (t, 2H, J = 5.9 Hz), 3.33-3.32 (m, 2H), 1.99-1.94 (m, 2H), 1.44 (s, 9H), 1.34 (s, 12H). ^{13}C NMR ($CDCl_3$, 126 MHz): 158.6, 156.1, 129.1, 127.5, 119.8, 118.4, 84.0, 66.0, 38.3, 29.6, 28.6, 25.0. 1 x C bearing B not observed. ^{11}B NMR ($CDCl_3$, 128 MHz): 30.9. ν_{max} (neat): 3375, 2980, 2932, 1688, 1534 cm^{-1}. HRMS: exact mass calculated for $[M+H]^+$ ($C_{20}H_{33}BNO_5$) requires 378.2446 *m/z*, found 378.2441 *m/z*

Preparation of *tert*-butyl (3-(4-(4,4,5,5-tetramethyl-1,3,2-dioxaborolan-2-yl)phenoxy)propyl)carbamate **S7**

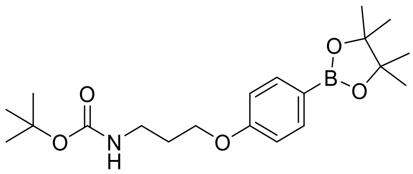

Prepared according to General Procedure C, using 4-hydroxyphenyl boronic acid pinacol ester (250 mg, 1.13 mmol, 1.0 equiv.), *tert*-butyl (3-hydroxypropyl)carbamate (257 mg, 1.47 mmol, 1.3 equiv.), CMBP (471 μL, 1.09 mmol, 2.0 equiv.) in anhydrous toluene (4 mL). The crude material was subjected to the purification outlined in General Procedure C (silica gel, 0-7% EtOAc in hexane) then washed with saturated Na_2CO_3 (3 x 20 mL) to afford the desired product

S7 as a clear oil (250 mg, 74%). ^{1}H NMR ($CDCl_3$, 500 MHz): 7.74 (d, *J* = 8.5 Hz, 2H), 6.88 (d, *J* = 8.5 Hz, 2H), 4.76 (br. s, 1H), 4.04 (t, *J* = 6.0 Hz, 2H), 3.33-3.31 (m, 2H), 2.00-1.95 (m, 2H) 1.44 (s, 9H), 1.33 (s, 12H). ^{13}C NMR ($CDCl_3$, 101 MHz): 161.5, 156.1, 136.7, 114.0, 83.7, 79.4, 65.7, 38.1, 29.6, 28.5, 25.0. 1 x C bearing B not observed. ^{11}B NMR ($CDCl_3$, 128 MHz): 31.7. ν_{max} (neat): 3357, 2977, 2932, 1694, 1606, 1517 cm^{-1}. HRMS: exact mass calculated for $[M+H]^+$ ($C_{20}H_{33}BNO_5$) requires 378.2446 *m/z*, found 378.2441 *m/z*.

Preparation of benzyl (*R*)-3-(4-(4,4,5,5-tetramethyl-1,3,2-dioxaborolan-2-yl)phenoxy)pyrrolidine-1-carboxylate **S9**

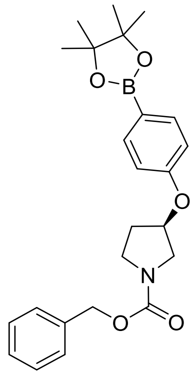

Prepared according to General Procedure C, using (*S*)-benzyl 3-hydroxypyrrolidine-1-carboxylate (301 mg, 1.36 mmol, 1.5 equiv.) 4-hydroxyphenol boronic acid pinacol ester (200 mg, 0.91 mmol, 1.0 equiv.), CMBP (477 µL, 1.82 mmol, 2.0 equiv.) in anhydrous toluene (5 mL). The crude material was subjected to the purification outlined in General Procedure C (silica gel, 0-12% EtOAc in hexanes) to afford the desired product **S9** as a yellow gum (298 mg, 79 %). ^{1}H NMR ($CDCl_3$, 500 MHz, 300 K): 7.75 (d, 2H, *J* = 8.0 Hz), 7.39-7.30 (m, 5H), 6.85 (d, 2H, *J* = 8.0 Hz), 5.19-5.09 (m, 2H), 4.95 (br. s, 1H), 3.72-3.56 (m, 4H), 2.23-2.20 (m, 1H), 2.15-2.06 (m, 1H), 1.33 (s, 12H). Rotameric mixture observed. ^{1}H NMR Variable Temperature (DMSO-d_6, 400 MHz, 373 K): 7.62 (d, 2H, *J* = 8.5 Hz), 7.35-7.28 (m, 5H), 6.93 (d, 2H, *J* = 8.5 Hz), 5.09-5.04 (m, 3H), 3.69-3.65 (m, 1H), 3.57-3.47 (m, 3H), 2.25-2.16 (m, 1H), 2.11-2.05 (m, 1H), 1.30 (s, 12H). ^{13}C NMR ($CDCl_3$, 126 MHz, 300 K): 159.8, 159.7,

154.98, 154.96, 137.0, 136.9, 136.8, 128.6, 128.08, 128.06, 114.9, 83.7, 76.1, 75.3, 67.0, 52.1, 51.6, 44.5, 44.1, 31.6, 30.8, 25.0. 1 x C bearing B not observed. Rotameric mixture observed. ^{13}C NMR Variable Temperature (DMSO-d_6, 101 MHz, 373 K): 159.2, 153.6, 136.7, 135.7, 127.8, 127.1, 126.8, 114.7, 82.9, 75.5, 65.4, 50.9, 43.4, 30.1, 24.1. 1 x C bearing B not observed. ^{11}B NMR ($CDCl_3$, 128 MHz): 30.6. ν_{max} (neat): 2979, 2887, 1700, 1605, 1415 cm^{-1}. HRMS: exact mass calculated for $[M+H]^+$ ($C_{24}H_{31}BNO_5$) requires 424.2290 *m/z*, found 424.2278 *m/z*.

Preparation of (*S*)-benzyl 3-(4-(4, 4, 5, 5-tetramethyl-1, 3, 2-dioxaborolan-2-yl)phenoxy)pyrrolidine-1-carboxylate **S10**

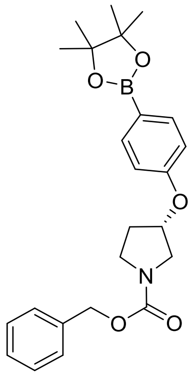

Prepared according to General Procedure C, using (*R*)-(-)-1-Cbz-3-hydroxypyrrolidine (301 mg, 1.36 mmol, 1.5 equiv.) 4-hydroxyphenol boronic acid pinacol ester (200 mg, 0.91 mmol, 1.0 equiv.), CMBP (477 µL, 1.82 mmol, 2.0 equiv.) in anhydrous toluene (5 mL). The crude material was subjected to the purification outlined in General Procedure C (silica gel, 0-12% EtOAc in hexane) to afford the desired product **S10** as a pale yellow gum (307 mg, 77%).^{1}H NMR Variable Temperature (DMSO-d_6, 400 MHz, 373 K): 7.63 (d, 2H, J = 8.3 Hz), 7.37-7.30 (m, 5H), 6.94 (d, 2H, J = 8.3 Hz), 5.10 (br. s, 2H), 5.07 (br. s, 1H), 3.69-3.66 (m, 1H), 3.56-3.45 (m, 3H), 2.25-2.17 (m, 1H), 2.10-2.07 (m, 1H), 1.30 (s, 12H). ^{13}C NMR Variable Temperature (DMSO-d_6, 101 MHz, 373 K): 159.2, 153.6, 136.7, 135.7, 127.8, 127.1, 126.8,

114.7, 82.9, 75.4, 65.4, 50.9, 43.4, 30.1, 24.1. 1 x C bearing B not observed. ^{11}B NMR ($CDCl_3$, 128 MHz): 30.9. ν_{max} (neat): 2977, 2866, 1705, 1606, 1415 cm^{-1}. HRMS: exact mass calculated for $[M+H]^+$ ($C_{24}H_{31}BNO_5$) requires 424.2290 *m/z*, found 424.2284 *m/z*.

Preparation of (4*R*)-4-((3*R*,7*S*,10*S*,13*R*)-3,7-dihydroxy-10,13-dimethylhexadecahydro-1*H*-cyclopenta[*a*]phenanthren-17-yl)-*N*-(2-(4-sulfamoylphenoxy)ethyl)pentanamide **11**

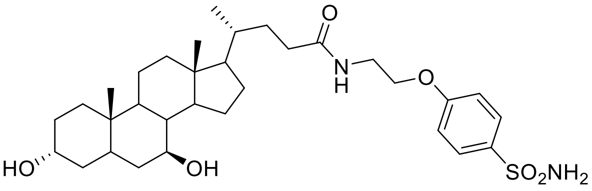

tert-butyl (2-(4-sulfamoylphenoxy)ethyl)carbamate **S2** was subjected to Boc deprotection conditions outlined in General Procedure B. Compound **11** was then prepared according to amidation conditions outlined in General Procedure B, using UDCA (106 mg, 0.27 mmol, 1.0 equiv.), DIPEA (237 µL, 1.36 mmol, 4.0 equiv.) and HATU (114 mg, 0.30 mmol, 1.1 equiv) in DMF (2 mL). The crude material was subjected to the purification outlined in General Procedure B (silica gel, 0-10% MeOH in DCM) to afford the desired product **11** as an off-white solid (56 mg, 30% over two steps). ^{1}H NMR (DMSO-d_6, 500 MHz): 8.04 (t, J = 5.6 Hz, 1H), 7.73 (d, J = 8.9 Hz, 2H), 7.19 (s, 2H), 7.07 (d, J = 8.9 Hz, 2H), 4.42 (d, J = 4.6 Hz, 1H), 4.04 (t, J = 5.6 Hz, 2H), 3.86 (d, J = 6.8 Hz, 1H), 3.41 (q, J = 5.5 Hz, 2H), 3.30-3.24 (m, coincident with solvent peak), 3.17 (d, J = 5.3 Hz, 2H), 2.14-2.09 (m, 1H), 2.03-1.97 (m, 1H), 1.92-1.90 (m, 1H), 1.85-1.79 (m, 1H), 1.77-1.72 (m, 1H), 1.68-1.63 (m, 3H), 1.49-1.43 (m, 3H), 1.38-1.27 (m, 6H), 1.21-1.07 (m, 6H), 1.02-0.91 (m, 2H), 0.88-0.87 (m, 6H), 0.58 (s, 3H). ^{13}C NMR (DMSO-d_6, 126 MHz): 172.9, 160.8, 136.3, 127.7, 114.5, 69.7, 69.5, 66.8, 55.9, 54.7, 43.1, 43.0, 42.2, 38.7, 38.0, 37.7, 37.3, 34.9, 34.8, 33.7, 32.3, 31.6, 30.2, 28.2, 26.7, 23.3, 20.8, 18.4, 12.0. 1 x C not observed. ν_{max} (neat): 3634, 3566, 3396, 3313, 2928, 2863, 1649,

1630 cm^{-1}. HRMS: exact mass calculated for $[M+H]^+$ ($C_{32}H_{51}N_2O_6S$) requires 591.3462 *m/z*, found 591.3462 *m/z*.

Preparation of (4*R*)-4-((3*R*,7*S*,10*S*,13*R*)-3,7-dihydroxy-10,13-dimethylhexadecahydro-1*H*-cyclopenta[*a*]phenanthren-17-yl)-*N*-(4-(4-sulfamoylphenoxy)butyl)pentanamide **12**

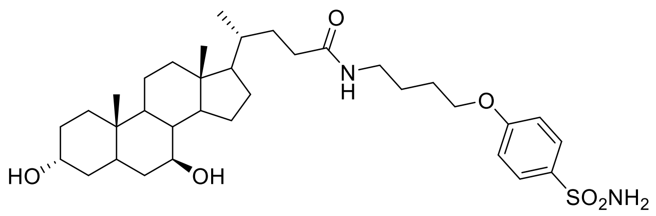

tert-butyl (2-(4-sulfamoylphenoxy)ethyl)carbamate **S3** (150 mg, 0.44 mmol, 1.0 equiv.) was subjected to Boc deprotection conditions outlined in General Procedure B. Compound **12** was then prepared according to amidation conditions outlined in General Procedure B, using UDCA (114 mg, 0.22 mmol, 1.0 equiv.), DMF (3 mL), DIPEA (250 μL, 1.46 mmol, 5.0 equiv.) and HATU (122 mg, 0.32 mmol, 1.1 equiv). The crude material was subjected to the purification outlined in General Procedure B (silica gel, 0-10% MeOH in DCM) to afford the desired product **37** as a clear solid (96 mg, 36% over two steps).^{1}H NMR (DMSO-d_6, 500 MHz): 7.77 (t, *J* = 5.4 Hz, 1H), 7.73 (d, *J* = 8.8 Hz, 2H), 7.18 (s, 2H), 7.06 (d, *J* = 8.8 Hz, 2H), 4.42 (d, *J* = 4.5 Hz, 1H), 4.04 (t, *J* = 6.4 Hz, 2H), 3.85 (d, *J* = 6.8 Hz, 1H), 3.29-3.25 (m, coincident with solvent peak), 3.10-3.06 (m, 2H), 2.11-2.05 (m, 1H), 1.99-1.92 (m, 2H), 1.87-1.81 (m, 1H), 1.76-1.63 (m, 6H), 1.55-1.44 (m, 5H), 1.41-1.28 (m, 7H), 1.23-1.08 (m, 6H), 1.03-0.96 (m, 1H), 0.89-0.87 (m, 6H), 0.60 (s, 3H). ^{13}C NMR (DMSO-d_6, 101 MHz): 172.4, 161.0, 136.0, 127.6, 114.4, 69.7, 69.4, 67.6, 55.9, 54.9, 43.1, 43.0, 42.1, 38.7, 37.9, 37.7, 37.3, 34.9, 34.8, 33.7, 32.4, 31.7, 30.2, 28.1, 26.7, 26.0, 25.7, 23.3, 20.8, 18.5, 12.0. 1 x C not observed. ν_{max} (neat): 3293, 3078, 2927, 2862, 1644, 1549, 1501, 1452 cm^{-1}. HRMS: exact mass calculated for $[M-H]^+$ ($C_{34}H_{53}N_2O_6S$) requires 617.3630 *m/z*, found 617.3627 *m/z*.

Preparation of (4*R*)-4-((3*R*,7*S*,10*S*,13*R*)-3,7-dihydroxy-10,13-dimethylhexadecahydro-1*H*-cyclopenta[*a*]phenanthren-17-yl)-*N*-(2-(3-(4,4,5,5-tetramethyl-1,3,2-dioxaborolan-2-yl)phenoxy)ethyl)pentanamide **13**

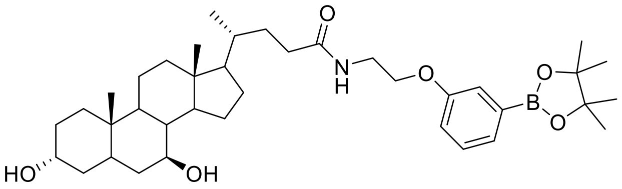

tert-Butyl (2-(3-(4,4,5,5-tetramethyl-1,3,2-dioxaborolan-2-yl)phenoxy)ethyl)carbamate **S4** (183 mg, 0.50 mmol, 1.0 equiv.) was subjected to Boc deprotection conditions outlined in General Procedure B. Compound **13** was then prepared according to amidation conditions outlined in General Procedure B, using UDCA (196 mg, 0.50 mmol, 1.0 equiv.), DIPEA (350 μL, 2.00 mmol, 4.0 equiv.) and HATU (209 mg, 0.55 mmol, 1.1 equiv) in DMF (5 mL). The crude material was subjected to the purification outlined in General Procedure B (silica gel, 0-4% MeOH in DCM) to afford the desired product **13** as a clear solid (130 mg, 44% over two steps). ^{1}H NMR ($CDCl_3$, 500 MHz): 7.41 (d, *J* = 7.2 Hz, 1H), 7.32-7.27 (m, 2H), 6.99-6.97 (m, 1H), 6.01 (br. s, 1H), 4.06 (t, *J* = 5.0 Hz, 2H), 3.66-3.63 (m, 2H), 3.59-3.55 (m, 2H), 2.28-2.22 (m, 1H), 2.11-2.05 (m, 1H), 1.98-1.96 (m, 1H), 1.90-1.85 (m, 1H), 1.79-1.77 (m, 5H), 1.67-1.64 (m, 2H), 1.60-1.56 (m, 2H), 1.49-1.38 (m, 6H), 1.33 (s, 12H), 1.27-1.12 (m, 6H), 1.12-0.97 (m, 3H), 0.93-0.91 (m, 6H), 0.64 (s, 3H). ^{13}C NMR ($CDCl_3$, 126 MHz): 173.8, 158.1, 129.2, 127.8, 119.9, 118.1, 84.0, 71.6, 71.5, 67.0, 55.9, 55.1, 44.0, 43.9, 42.6, 40.3, 39.3, 39.1, 37.5, 37.0, 35.5, 35.1, 34.2, 33.7, 31.9, 30.5, 28.8, 27.0, 25.0, 23.5, 21.3, 18.6, 12.3. 1 x C bearing B not observed. ^{11}B NMR ($CDCl_3$, 128 MHz): 30.8. ν_{max} (neat): 3330, 2926, 2863, 1651, 1547, 1428 cm^{-1}. HRMS: exact mass calculated for $[M+H]^+$ ($C_{38}H_{61}BNO_6$) requires 638.4593 *m/z*, found 638.4580 *m/z.*

Preparation of (4*R*)-4-((3*R*,7*S*,10*S*,13*R*)-3,7-dihydroxy-10, 13-dimethylhexadecahydro-1*H*-cyclopenta[*a*]phenanthren-17-yl)-*N*-(2-(4-(4,4,5,5-tetramethyl-1,3,2-dioxaborolan-2-yl)phenoxy)ethyl)pentanamide **14**

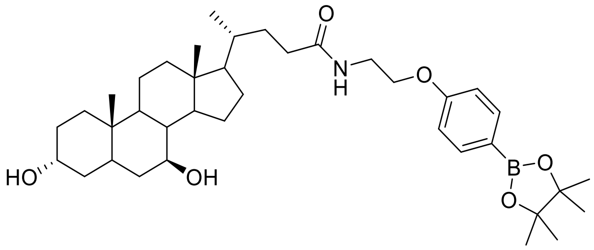

tert-Butyl (2-(4-(4,4,5,5-tetramethyl-1,3,2-dioxaborolan-2-yl)phenoxy)ethyl)carbamate **S5** (207 mg, 0.57 mmol, 1.0 equiv.) was subjected to Boc deprotection conditions outlined in General Procedure B. Compound **14** was prepared according to amidation conditions outlined in General Procedure B, using ursodeoxycholic acid (247 mg, 0.63 mmol, 1.0 equiv.), DIPEA (436 µL, 2.50 mmol, 4.0 equiv.) and HATU (262 mg, 0.69 mmol, 1.1 equiv) in DMF (5 mL). The crude material was subjected to the purification outlined in General Procedure B (silica gel, 0-3% MeOH in DCM) to afford the desired product **14** as a clear solid (131 mg, 33% over two steps). ^{1}H NMR ($CDCl_3$, 500 MHz): 7.74 (d, *J* = 8.2 Hz, 2H), 6.87 (d, *J* = 8.3 Hz, 2H), 5.96-5.94 (m, 1H), 4.06-4.04 (m, 2H), 3.68-3.64 (m, 2H), 3.59-3.54 (m, 2H), 2.29-2.23 (m, 1H), 2.12-2.06 (m, 1H), 1.98-1.96 (m, 1H), 1.92-1.84 (m, 1H), 1.82-1.75 (m, 4H), 1.67-1.65 (m, 2H), 1.60-1.56 (m, 2H), 1.49-1.38 (m, 8H), 1.33 (s, 12H), 1.27-1.23 (m, 3H), 1.15-1.00 (m, 4H), 0.93-0.91 (m, 6H), 0.64 (s, 3H). ^{13}C NMR ($CDCl_3$, 126 MHz): 173.8, 161.2, 136.8, 113.9, 83.8, 71.6, 71.5, 66.9, 55.9, 55.1, 43.9, 42.6, 40.3, 39.3, 39.0, 37.5, 37.0, 35.5, 35.1, 34.2, 33.7, 31.9, 30.5, 28.8, 27.0, 25.0, 23.5, 21.3, 18.6, 12.3. 1 x C bearing B not observed. ^{11}B NMR ($CDCl_3$, 128 MHz): 31.2. ν_{max} (neat): 3421, 2928, 2861, 1651, 1604, 1547, 1454 cm^{-1}. HRMS: exact mass calculated for $[M+H]^+$ ($C_{38}H_{61}BNO_6$) requires 638.4593 *m/z*, found 638.4582 *m/z.*

Preparation of (4*R*)-4-((3*R*,7*S*,10*S*,13*R*)-3,7-dihydroxy-10,13-dimethylhexadecahydro-1*H*-cyclopenta[*a*]phenanthren-17-yl)-*N*-(3-(4-(4,4,5,5-tetramethyl-1,3,2-dioxaborolan-2-yl)phenoxy)propyl)pentanamide **15**

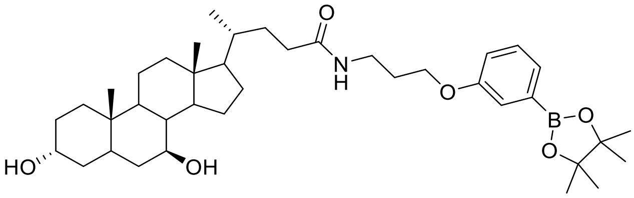

tert-Butyl (3-(3-(4,4,5,5-tetramethyl-1,3,2-dioxaborolan-2-yl)phenoxy)propyl)carbamate **S6** (300 mg, 0.80 mmol, 1.0 equiv.) was subjected to Boc deprotection conditions outlined in General Procedure B. Compound **15** was prepared according to amidation conditions outlined in General Procedure B, using UDCA (345 mg, 0.88 mmol, 1.0 equiv.), DIPEA (614 µL, 3.52 mmol, 4.0 equiv.) and HATU (368 mg, 0.97 mmol, 1.1 equiv) in DMF (4 mL). The crude material was subjected to the purification outlined in General Procedure B (silica gel, 0-4% MeOH in DCM) to afford the desired product **15** as a clear solid (190 mg, 33% over two steps). ^{1}H NMR ($CDCl_3$, 500 MHz): 7.42 (d, *J* = 7.2 Hz, 1H), 7.32-7.28 (m, 2H), 6.99 (dd, *J* = 8.1, 2.4 Hz, 1H), 5.84 (br. s, 1H), 4.08 (t, *J* = 5.6 Hz, 2H), 3.61-3.56 (m, 2H), 3.48-3.45 (m, 2H), 2.27-2.21 (m, 1H), 2.09-1.97 (m, 4H), 1.93-1.85 (m, 1H), 1.82-1.76 (m, 4H), 1.68-1.66 (m, 2H), 1.61-1.57 (m, 2H), 1.51-1.40 (m, 7H), 1.34 (s, 12H), 1.30-1.20 (m, 5H), 1.18-1.01 (m, 4H), 0.94-0.93 (m, 6H), 0.67 (s, 3H). ^{13}C NMR ($CDCl_3$,126 MHz): 173.6, 158.1, 129.1, 127.5, 119.60, 118.2, 83.9, 71.5, 71.4, 66.5, 55.7, 54.9, 43.8, 43.8, 42.5, 40.2, 39.2, 37.6, 37.3, 36.8, 35.4, 35.0, 34.1, 33.7, 31.9, 30.4, 28.9, 28.7, 26.9, 24.9, 23.4, 21.2, 18.5, 12.2. 1 x C bearing B not observed. ^{11}B NMR ($CDCl_3$, 160 MHz): 31.0. ν_{max} (neat): 3811, 2928, 2863, 1573, 1548, 1649, 1428 cm^{-1}. HRMS: exact mass calculated for $[M+H]^+$ ($C_{39}H_{63}BNO_6$) requires 652.4750 *m/z*, found 652.4737 *m/z*.

Preparation of (4*R*)-4-((3*R*,7*S*,10*S*,13*R*)-3,7-dihydroxy-10,13-dimethylhexadecahydro-1*H*-cyclopenta[*a*]phenanthren-17-yl)-*N*-(3-(4-(4,4,5,5-tetramethyl-1,3,2-dioxaborolan-2-yl)phenoxy)propyl)pentanamide **16**

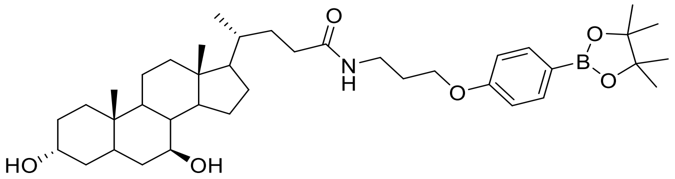

tert-Butyl (3-(4-(4,4,5,5-tetramethyl-1,3,2-dioxaborolan-2-yl)phenoxy)propyl)carbamate **S7** (231 mg, 0.61 mmol, 1.0 equiv.) was subjected to Boc deprotection conditions outlined in General Procedure B. Compound **16** was prepared according to amidation conditions outlined in General Procedure B, using ursodeoxycholic acid (239 mg, 0.61 mmol, 1.0 equiv.), DIPEA (424 µL, 2.43 mmol, 4.0 equiv.) and HATU (254 mg, 0.67 mmol, 1.1 equiv.) in DMF (3 mL). The crude material was subjected to the purification outlined in General Procedure B (silica gel, 0-3% MeOH in DCM) to afford the desired product **16** as a clear solid (247 mg, 60% over two steps). ^{1}H NMR ($CDCl_3$, 500 MHz): 7.75 (d, *J* = 8.3 Hz, 2H), 6.88 (d, *J* = 8.4 Hz, 2H), 5.81 (t, *J* = 5.3 Hz, 1H), 4.07 (t, *J* = 5.8 Hz, 2H), 3.63-3.54 (m, 2H), 3.45 (app. q, *J* = 6.2 Hz, 2H), 2.20-2.26 (m, 1H), 1.98-2.09 (m, 4H), 1.85-1.92 (m, 1H), 1.76-1.82 (m, 5H), 1.66-1.68 (m, 2H), 1.57-1.68 (m, 2H), 1.57-1.61 (m, 3H), 1.40-1.51 (m, 9H), 1.33 (s, 12 H), 0.99-1.17 (m, 4H), 0.94 (s, 3H), 0.92-0.93 (m, 3H), 0.67 (s, 3H). ^{13}C NMR ($CDCl_3$, 101 MHz): 173.7, 161.3, 136.8, 113.9, 83.8, 71.6, 71.5, 66.3, 55.9, 55.1, 43.9, 43.9, 42.6, 40.3, 39.3, 37.5, 37.5, 37.0, 35.5, 35.1, 34.2, 33.8, 32.0, 30.5, 29.1, 28.8, 27.0, 25.0, 23.5, 21.3, 18.7, 12.3. 1 x C bearing B not observed. ^{11}B NMR ($CDCl_3$, 160 MHz): 31.7. ν_{max} (neat): 3324, 2926, 2863, 1651, 1604, 1547, 1450 cm^{-1}. HRMS: exact mass calculated for $[M+H]^+$ ($C_{39}H_{63}BNO_6$) requires 652.4755 *m/z*, found 652.4756 *m/z*.

Preparation of potassium (4-(3-((4*R*)-4-((3*R*,7*S*,10*S*,13*R*)-3,7-dihydroxy-10,13-dimethylhexadecahydro-1*H*-cyclopenta[*a*]phenanthren-17-yl)pentanamido)propoxy)phenyl)trifluoroborate **17**

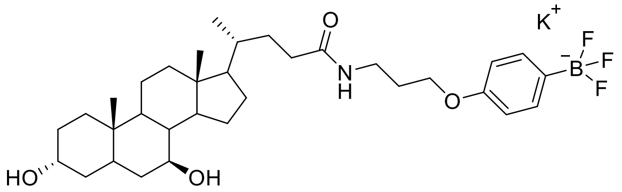

Procedure adapted from Lennox and Lloyd-Jones.[2] To a round-bottomed flask was added *tert*-Butyl (3-(4-(4,4,5,5-tetramethyl-1,3,2-dioxaborolan-2-yl)phenoxy)propyl)carbamate **16** (146 mg, 0.21 mmol, 1.0 equiv.) and MeOH (1 mL) followed by MeCN (1 mL). Potassium fluoride (52 mg, 0.89 mmol, 4.0 equiv) in H_2O (500 μL) was then added and the reaction mixture was stirred until complete dissolution of the boronic ester. *L*-(+)-tartaric acid (69 mg, 0.46 mmol, 2.05 equiv) was dissolved in THF (1 mL) and added dropwise to the rapidly (≈ 1000 RPM) stirring biphasic mixture over a period of 5 minutes, as a white precipitate formed. The reaction was stirred for 2 minutes, diluted with MeCN (3 mL) and stirred for a further 2 minutes before being diluted again with MeCN (1 mL) and filtered. The flask and filter were rinsed with further portions of acetonitrile (3 x 5 mL) and the combined filtrates were concentrated *in vacuo* to provide a mixture of the corresponding potassium organotrifluoroborate salt and pinacol. The filter cake was then washed with Et_2O (3 x 5 mL) to remove the excess pinacol leaving behind the product **17** as a white solid which was telescoped through to the next step as the trifluoroborate salt without further purification. ^{1}H NMR (DMSO-d_6, 500 MHz): 7.82 (t, *J* = 5.3 Hz, 1H), 7.20 (d, *J* = 8.1 Hz, 2H), 6.64 (d, *J* = 8.1 Hz, 2H), 4.45-4.37 (m, 1H), 3.89-3.84 (m, 3H), 3.17-3.14 (m, 2H), 2.11-2.05 (m, 1H), 1.99-1.92 (m, 2H), 1.87-1.73 (m, 4H), 1.71-1.63 (m, 3H), 1.51-1.46 (m, 3H), 1.41-1.27 (m, 8H), 1.21-1.07 (m, 7H), 1.03-0.91 (m, 2H), 0.89-0.87 (m, 6H), 0.61 (s, 3H). ^{13}C NMR (DMSO-d_6, 126 MHz): 172.5, 156.4, 132.2, 112.5,

69.7, 69.5, 64.7, 55.9, 54.7, 43.1, 43.0, 42.2, 38.7, 37.7, 37.3, 35.5, 34.9, 34.8, 33.8, 32.4, 31.7, 30.2, 29.1, 28.2, 26.7, 23.3, 20.8, 18.5, 12.0. 1 x C bearing B not observed, 1 x C not observed. ^{11}B NMR (DMSO-*d*$_6$, 160 MHz): 3.63. ^{19}F NMR (DMSO-*d*$_6$, 471 MHz): -138.3. ν_{max} (neat): 3364, 2930, 2865, 1646, 1605 cm^{-1}. HRMS: exact mass calculated for [M-K]$^-$ ($C_{33}H_{50}BF_3NO_4$) requires 592.3796 *m/z*, found 592.3796 *m/z.*

Preparation of (4-(3-((4*R*)-4-((3*R*,7*S*,10*S*,13*R*)-3,7-dihydroxy-10,13-dimethylhexadecahydro-1*H*-cyclopenta[*a*]phenanthren-17-yl)pentanamido)propoxy)phenyl)boronic acid **18**

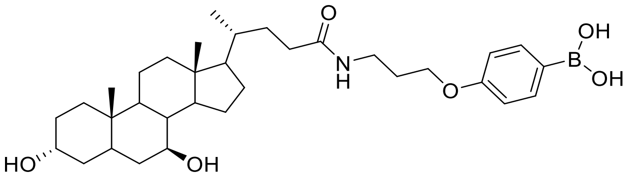

To a round-bottomed flask containing **17** (60 mg, 0.10 mmol, 1.0 equiv.) and excess SiO_2 under argon was added H_2O (3 mL). The reaction mixture was stirred at rt for 1 h before being was then filtered, and the filter cake was thoroughly washed with EtOAc. The organic phases were separated, and the aqueous phase was extracted with EtOAc (2x 10 mL). The combined organic phases were combined, washed with brine (10 mL), dried over $MgSO_4$, and concentrated *in vacuo* to afford title compound **18** as a white solid (32.1 mg, 43 %). ^{1}H NMR (DMSO-*d*$_6$, 400 MHz): 7.85-7.81 (m, 2H), 7.73-7.69 (m, 2H), 6.88-6.86 (m, 2H), 4.44-4.43 (m, 1H), 4.03-3.95 (m, 2H), 3.86-3.85 (m, 1H), 3.23-3.14 (m, 2H), 2.12-2.04 (m, 1H), 2.01-1.91 (m, 2H), 1.88-1.80 (m, 3H), 1.78-1.62 (m, 4H), 1.52-1.42 (m, 3H), 1.39-1.27 (m, 6H), 1.19-1.06 (m, 6H), 1.05-0.88 (m, 9H), 0.61 (s, 3H). ^{13}C NMR (DMSO-*d*$_6$, 101 MHz): 172.6, 160.3, 135.8, 113.4, 69.7, 69.5, 64.9, 55.9, 54.7, 43.1, 43.0, 42.2, 38.7, 37.7, 37.3, 35.4, 34.9, 34.8, 33.7, 32.4, 31.7, 30.2, 28.9, 28.2, 26.7, 23.3, 20.8, 18.5, 12.0. 1 x C bearing B not observed, 1 x C not observed. ^{11}B NMR (MeOD, 160 MHz): 28.4. ν_{max} (neat): 3322, 2924, 2854, 1651, 1602, 1513, 1454,

1411 cm^{-1}. HRMS: exact mass calculated for $[M+H]^+$ ($C_{33}H_{53}BNO_6$) requires 570.3966 *m/z*, found 570.3959 *m/z*.

Preparation of (4*R*)-4-((3*R*,7*S*,10*S*,13*R*)-3,7-dihydroxy-10,13-dimethylhexadecahydro-1*H*-cyclopenta[*a*]phenanthren-17-yl)-1-((*R*)-3-(4-(4,4,5,5-tetramethyl-1,3,2-dioxaborolan-2-yl)phenoxy)pyrrolidin-1-yl)pentan-1-one **20**

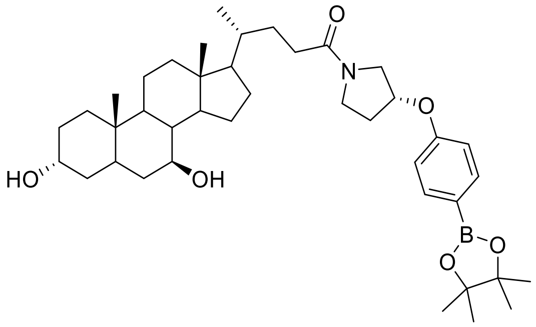

Benzyl (*R*)-3-(4-(4,4,5,5-tetramethyl-1,3,2-dioxaborolan-2-yl)phenoxy)pyrrolidine-1-carboxylate **S9** (269 mg, 0.63 mmol, 1.0 equiv.) was subjected to Cbz deprotection conditions in accordance with General Procedure B to generate intermediate **S9a**. Compound **20** was prepared according to amidation conditions outlined in General Procedure B using UDCA (302 mg, 0.77 mmol, 1.0 equiv.), **S9a** (231 mg, 0.77 mmol, 1.0 equiv), DMF (5 mL), DIPEA (405 µL, 2.32 mmol, 3.0 equiv.) and HATU (323 mg, 0.85 mmol, 1.1 equiv.). The crude material was subjected to the purification outlined in General Procedure B (silica gel, 0-6% MeOH in DCM) to afford the desired product **20** as a white solid (151 mg, 30%). ^{1}H NMR ($CDCl_3$, 500 MHz): 7.76-7.72 (m, 2H), 6.87-6.84 (m, 2H), 5.03-4.95 (m, 1H), 3.82-3.56 (m, 6H), 2.37-2.19 (m, 3H), 2.16-2.07 (m, 1H), 2.01-1.90 (m, 2H), 1.83-1.76 (m, 5H), 1.68-1.54 (m, 6H), 1.50-1.41 (m, 7H), 1.33 (s, 12H), 1.25-1.15 (m, 4H), 1.10-1.01 (m, 2H), 0.94-0.91 (m, 6H), 0.68-0.67 (m, 3H). Rotameric mixture observed. ^{13}C NMR ($CDCl_3$, 126 MHz): 172.6, 172.5, 159.7, 159.6, 136.82, 136.77, 114.94, 114.87, 83.82, 83.76, 76.3, 74.8, 71.6, 71.51, 71.48, 55.9, 55.8, 55.2, 55.1, 52.2, 51.6, 44.8, 43.9, 43.9, 42.6, 40.30, 40.27, 39.3, 37.5, 37.0, 35.6, 35.1, 34.2,

32.0, 31.8, 31.7, 31.1, 31.0, 30.5, 30.2, 28.8, 27.1, 25.0, 23.5, 21.3, 18.80, 18.75, 12.3. 1 x C bearing B not observed. Rotameric mixture observed. ^{11}B NMR ($CDCl_3$, 128 MHz): 31.1. ν_{max} (neat): 3407, 2930, 2867, 1629, 1606 cm^{-1}. HRMS: exact mass calculated for $[M+H]^+$ ($C_{40}H_{62}BNO_6Na$) requires 686.4569 *m/z*, found 686.4558 *m/z*.

Preparation of (4*R*)-4-((3*R*,7*S*,10*S*,13*R*)-3,7-dihydroxy-10,13-dimethylhexadecahydro-1*H*-cyclopenta[*a*]phenanthren-17-yl)-1-((*S*)-3-(4-(4,4,5,5-tetramethyl-1,3,2-dioxaborolan-2-yl)phenoxy)pyrrolidin-1-yl)pentan-1-one **21**

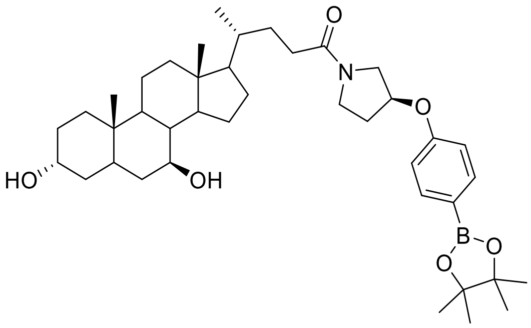

(*S*)-benzyl 3-(4-(4, 4, 5, 5-tetramethyl-1, 3, 2-dioxaborolan-2-yl)phenoxy)pyrrolidine-1-carboxylate **S10** (269 mg, 0.63 mmol, 1.0 equiv.) was subjected to Cbz deprotection conditions as outlined in General Procedure B to generate crude intermediate **S10a**. Compound **21** was prepared according to amidation conditions outlined in General Procedure B, using UDCA (255 mg, 0.65 mmol, 1.0 equiv.), **S10a** (194 mg, 0.65 mmol, 1.0 equiv), DMF (5 mL), DIPEA (340 μL, 1.95 mmol, 3.0 equiv.) and HATU (273 mg, 0.72 mmol, 1.1 equiv). The crude material was subjected to the purification outlined in General Procedure B (silica gel, 0-6% MeOH in DCM) to afford the desired product **21** as a white solid (194 mg, 46 %). ^{1}H NMR ($CDCl_3$, 500 MHz): 7.77-7.73 (m, 2H), 6.87-6.84 (m, 2H), 5.03-4.95 (m, 1H), 3.83-3.56 (m, 6H), 2.37-2.07 (m, 4H), 2.02-1.97 (m, 2H), 1.83-1.76 (m, 4H), 1.68-1.57 (m, 5H), 1.51-1.40 (m, 9H), 1.33 (s, 12H), 1.28-1.20 (m, 4H), 0.96-0.91 (m, 6H), 0.68-0.66 (m, 3H). Rotameric mixture observed. ^{13}C NMR ($CDCl_3$, 126 MHz): 172.6, 172.5, 159.7, 159.6, 136.83, 136.78,

114.94, 114.88, 83.83, 83.77, 76.3, 74.8, 71.6, 71.5, 55.9, 55.2, 52.3, 51.6, 44.8, 44.0, 43.94, 43.86, 42.6, 40.3, 39.3, 37.5, 36.9, 35.6, 35.5, 35.1, 34.2, 32.0, 31.9, 31.7, 31.01, 30.99, 30.5, 30.2, 28.81, 28.77, 27.1, 25.0, 23.5, 21.3, 18.8, 12.3. 1 x C bearing B not observed. Rotameric mixture observed. ^{11}B NMR ($CDCl_3$, 160 Hz): 31.1. ν_{max} (neat): 3418, 2930, 2867, 1606 cm^{-1}. HRMS: exact mass calculated for $[M+H]^+$ ($C_{40}H_{62}BNO_6Na$) requires 686.4569 *m/z*, found 686.4562 *m/z*.

4. ^{1}H NMR, ^{13}C NMR, ^{11}B NMR, ^{19}F NMR and HRMS Spectra

Spectra for intermediate **S1**

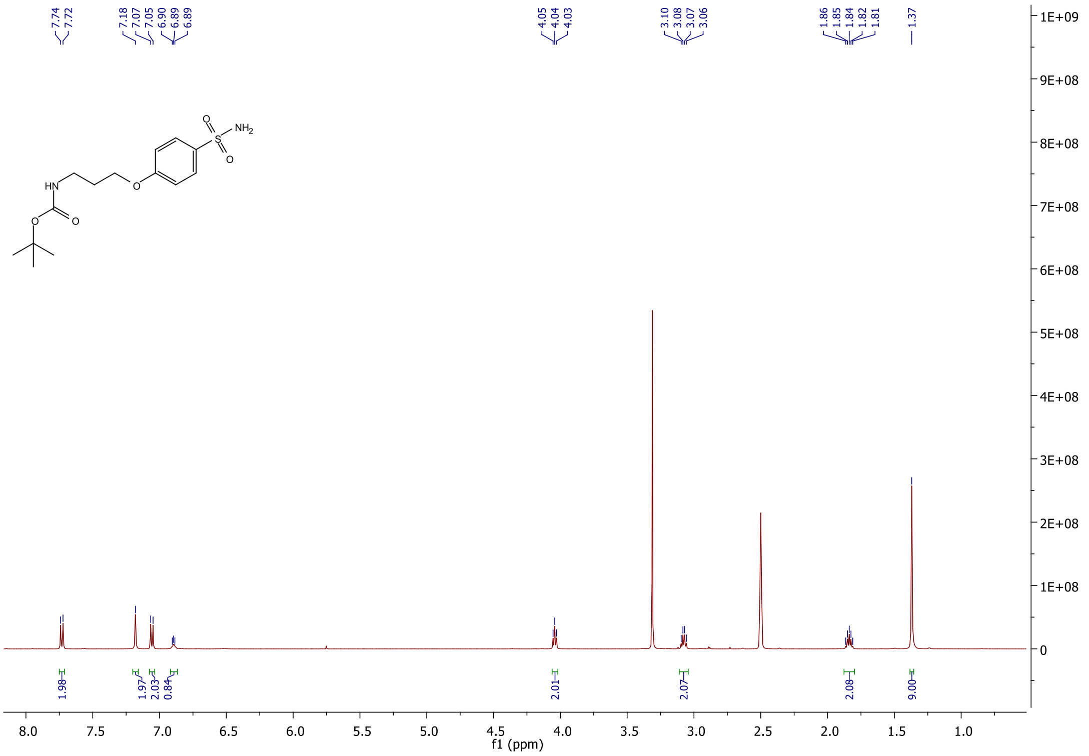

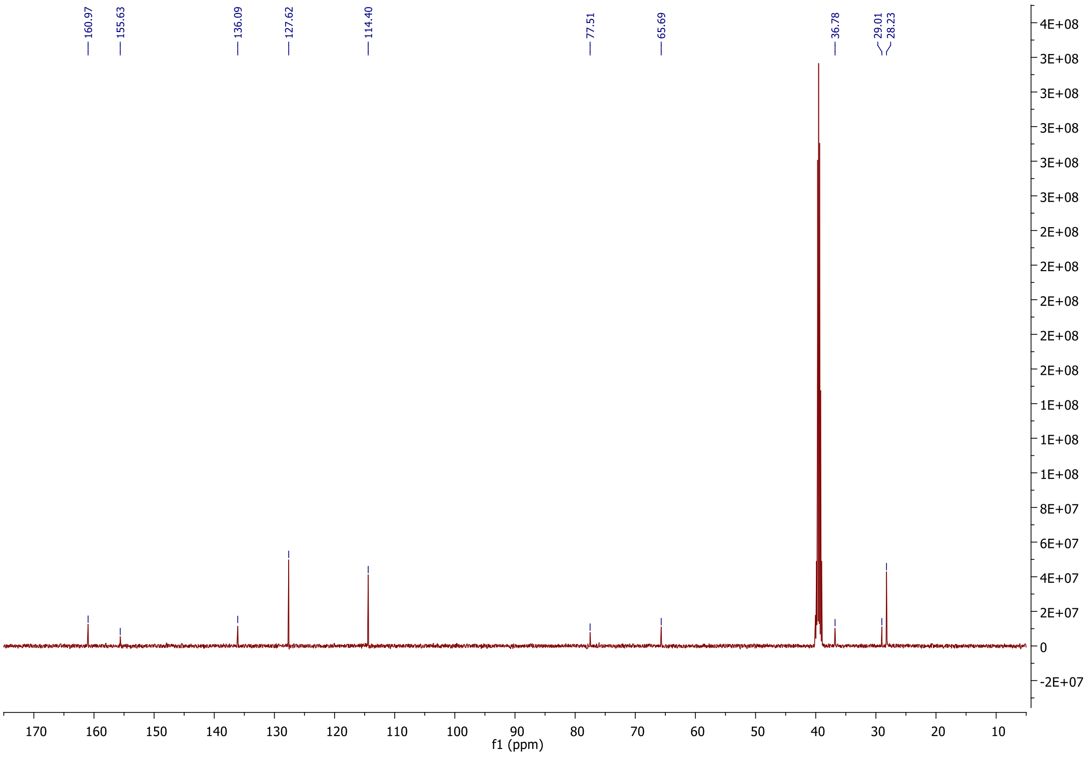

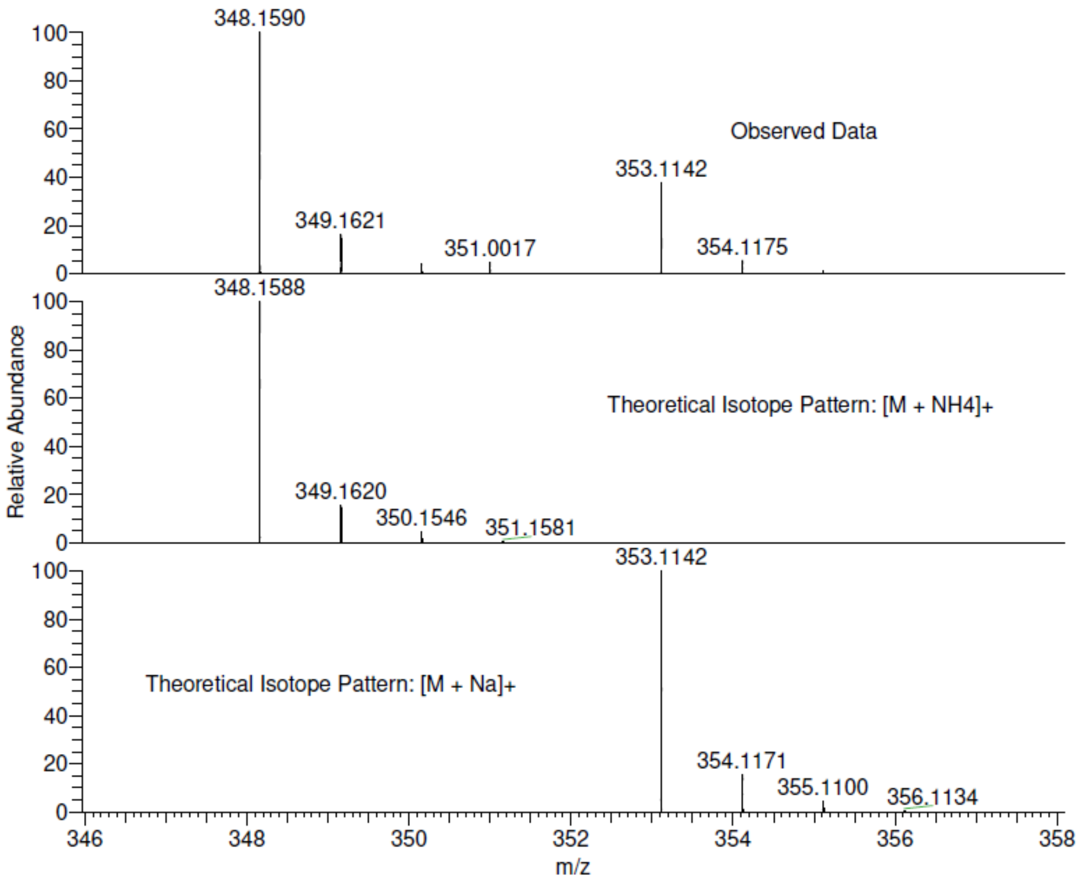

Spectra for intermediate **S2**

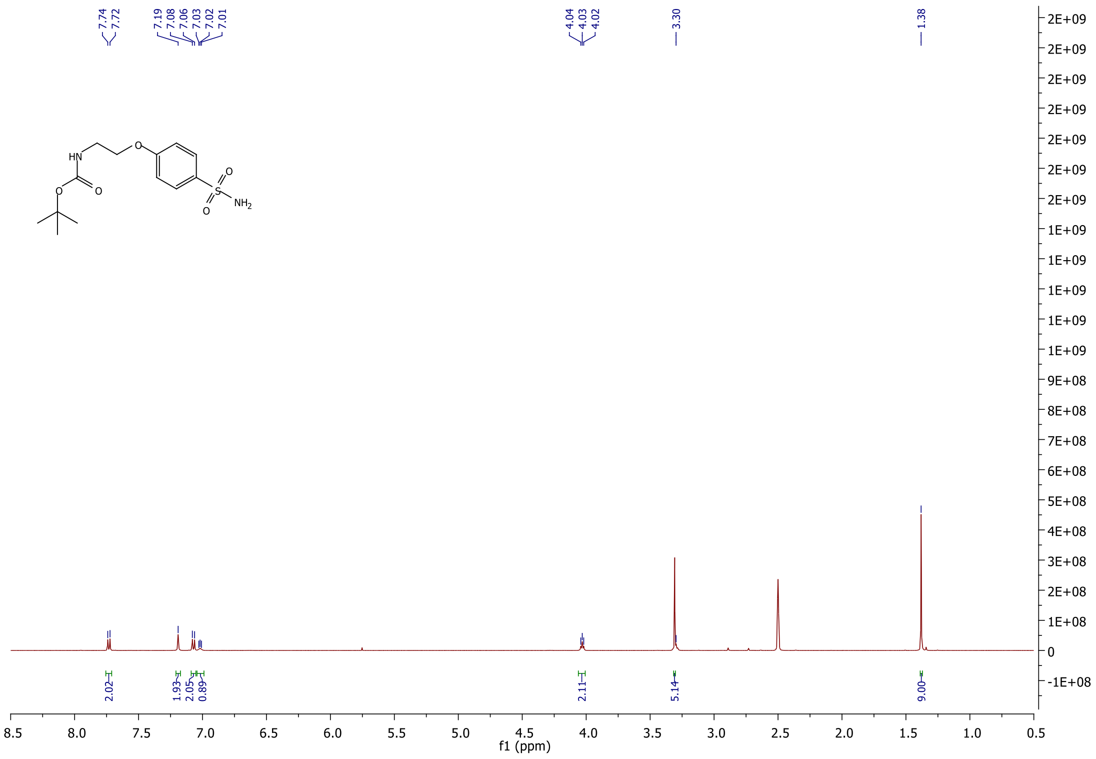

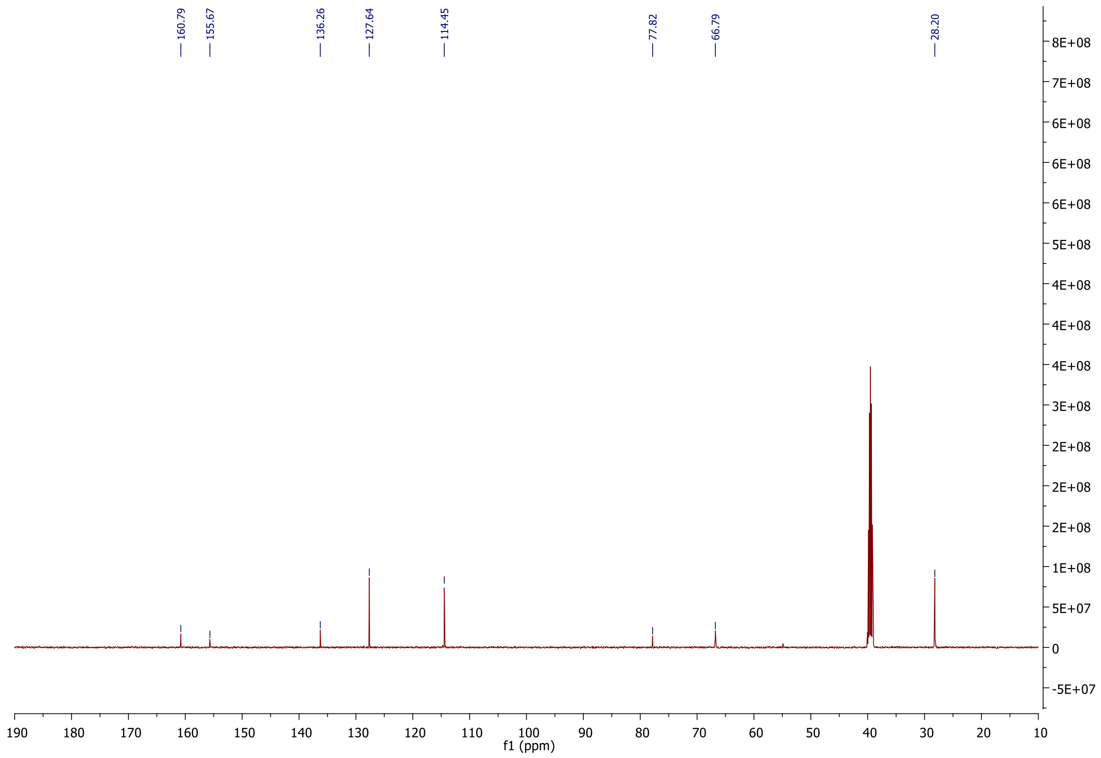

160.79
155.67
136.26
127.64
114.45
77.82
66.79
28.20
f1 (ppm)

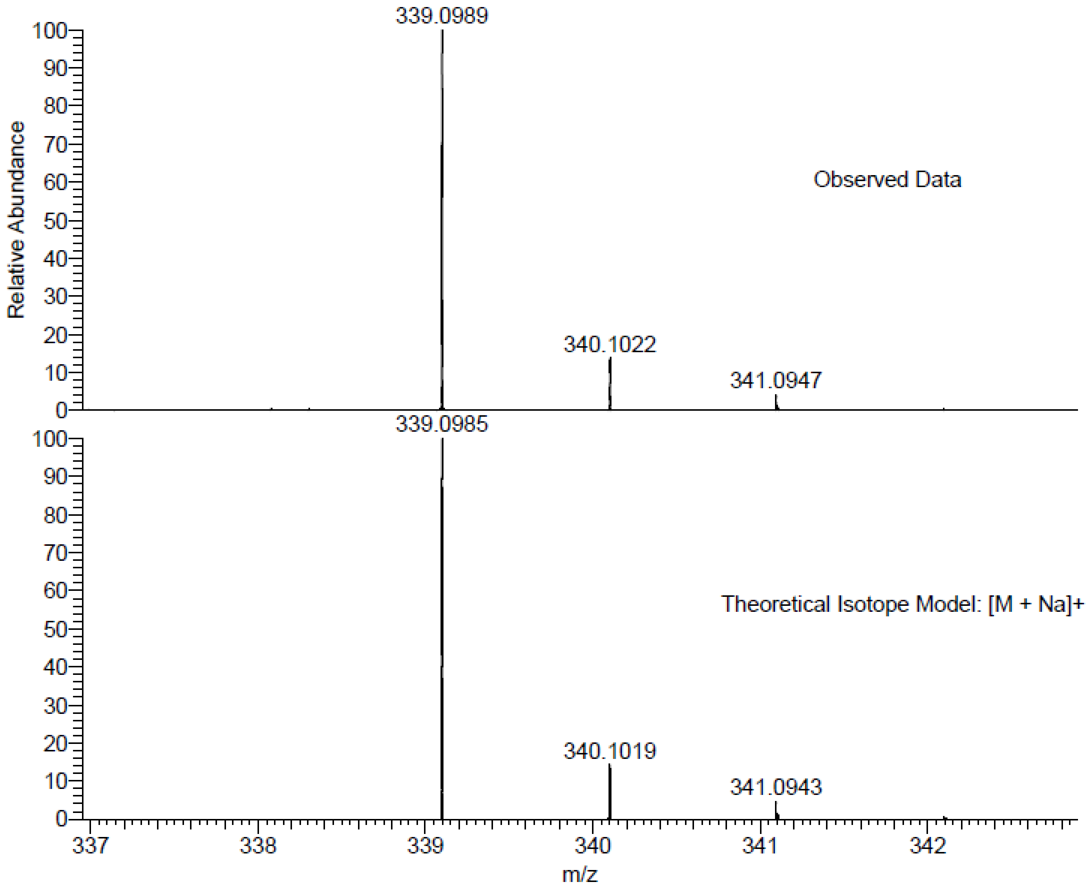

339.0989
Relative Abundance
Observed Data
340.1022
341.0947
339.0985
Theoretical Isotope Model: [M + Na]+
340.1019
341.0943
m/z

Spectra for intermediate **S3**

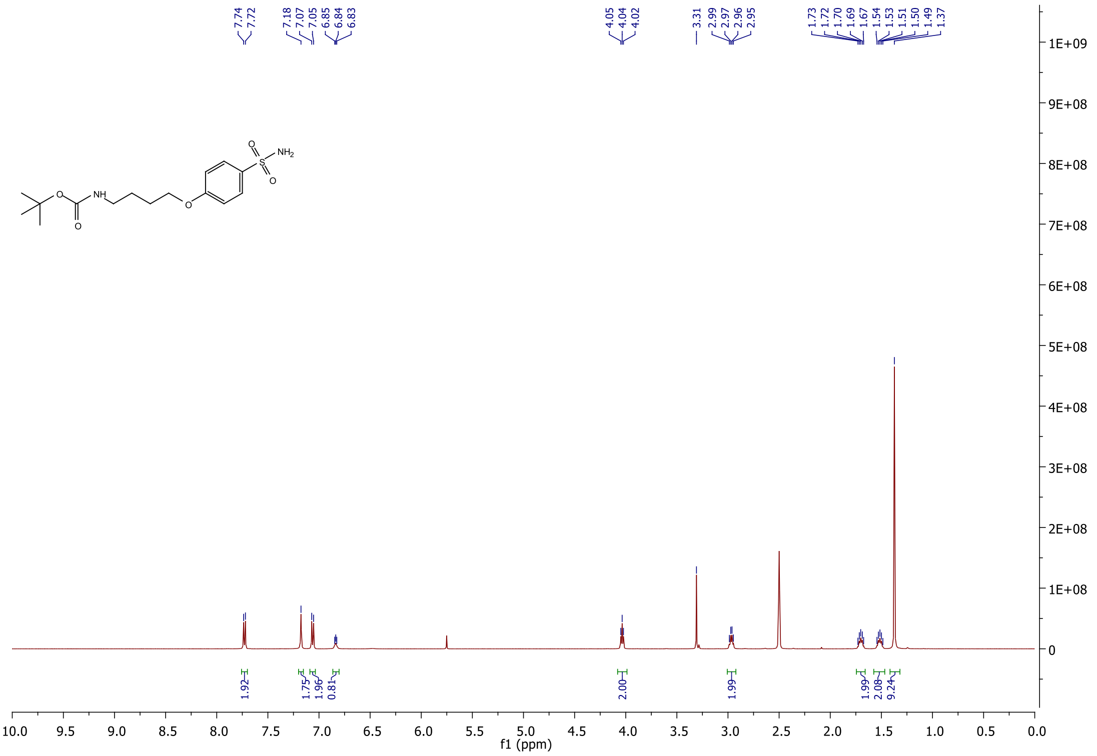

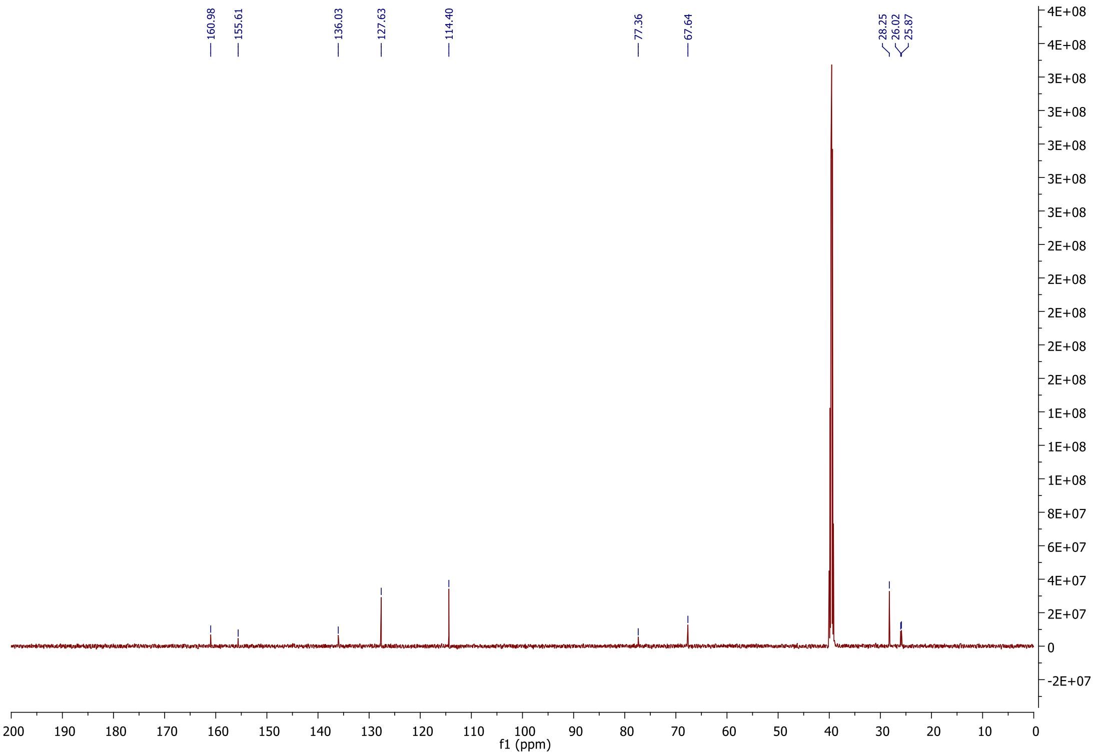

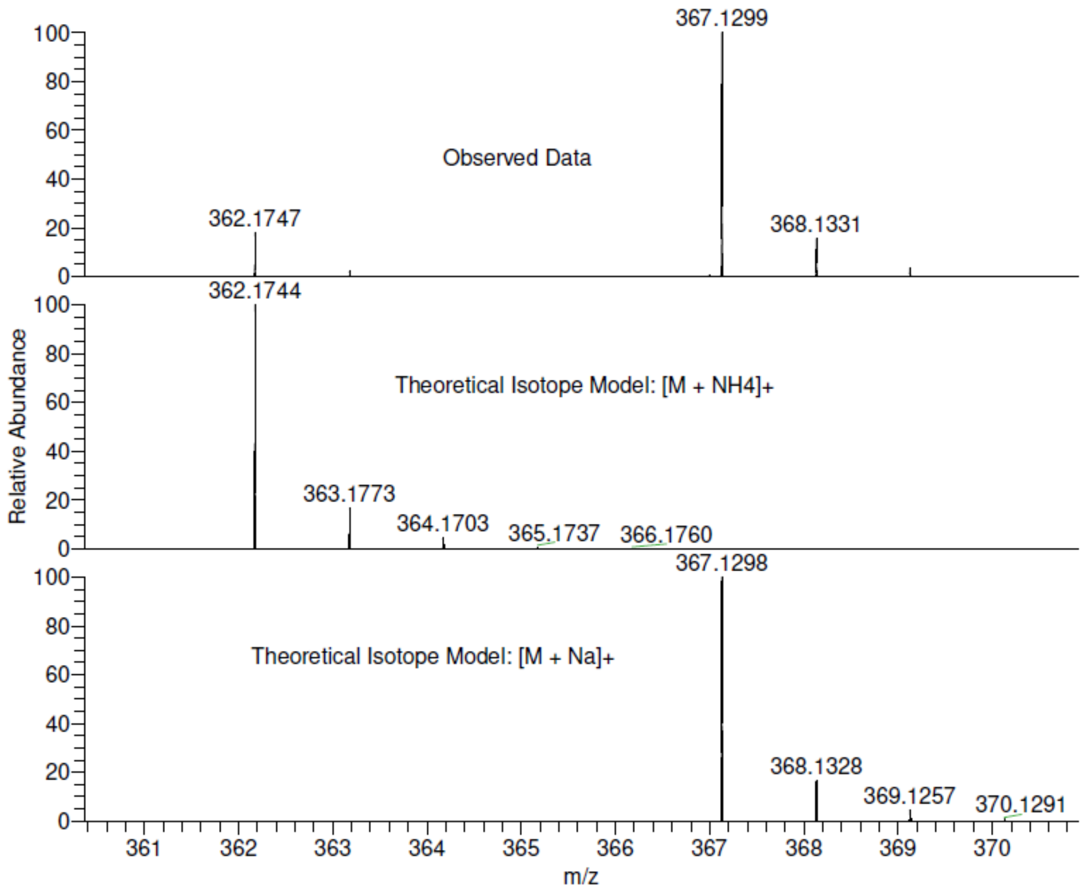

<u>Spectra for intermediate **S4**</u>

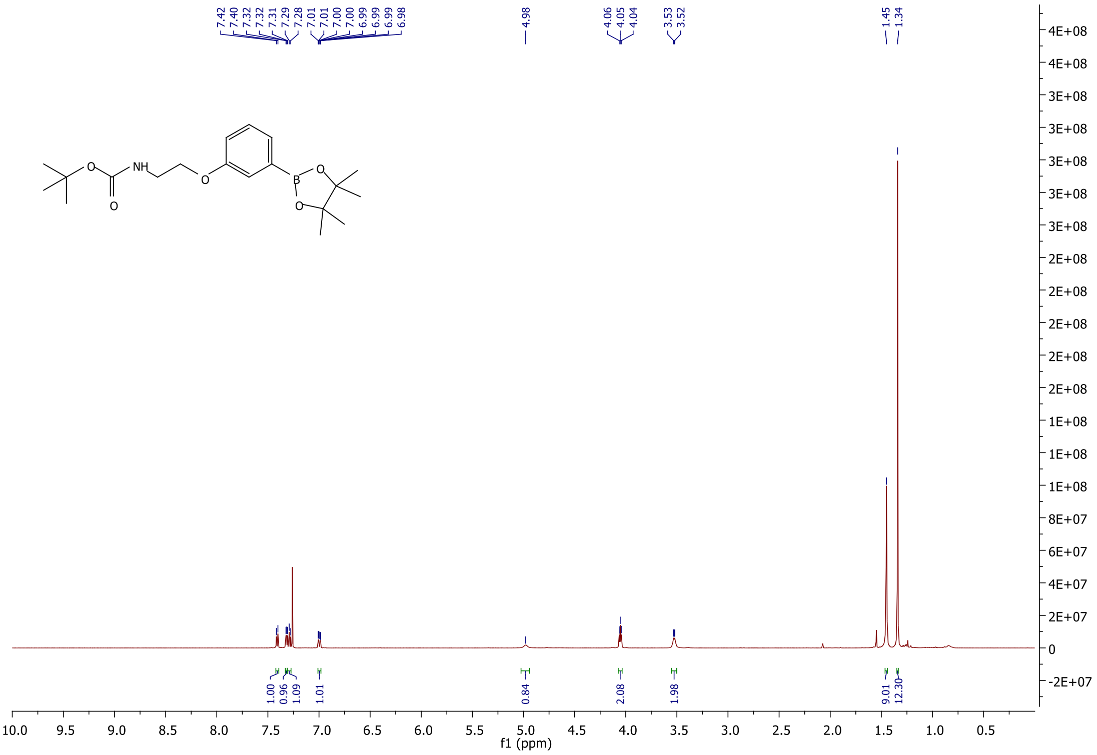

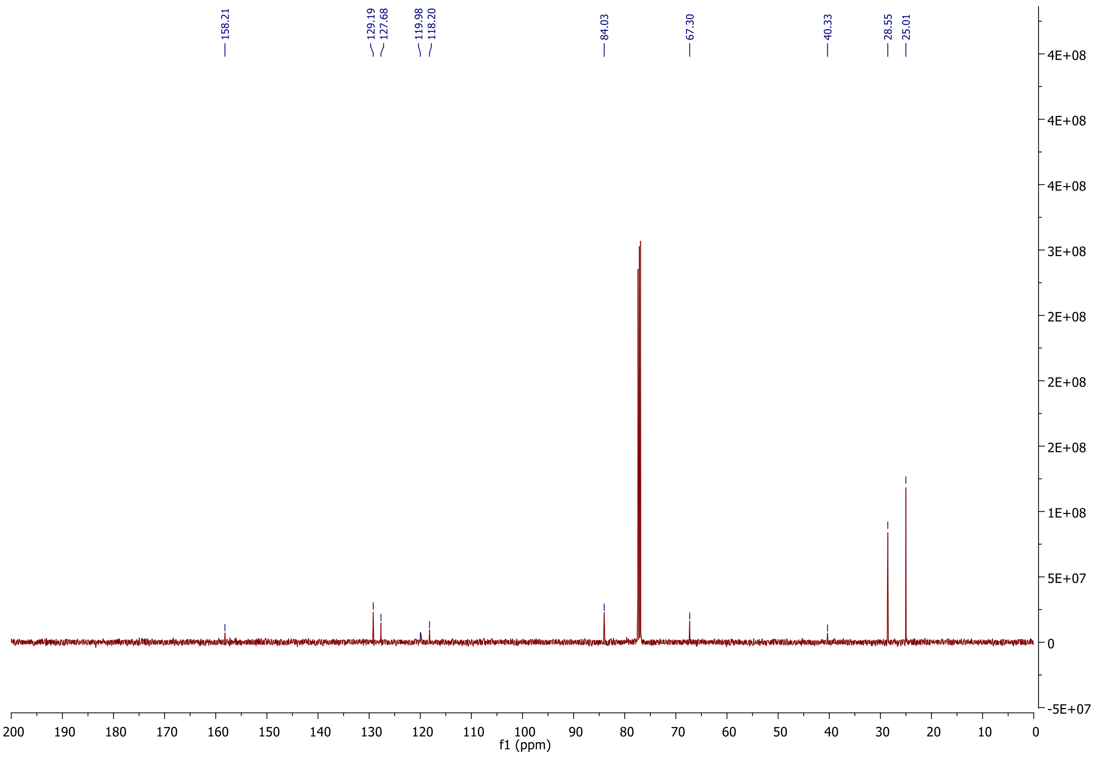
158.21
129.19
127.68
119.98
118.20
84.03
67.30
40.33
28.55
25.01
f1 (ppm)

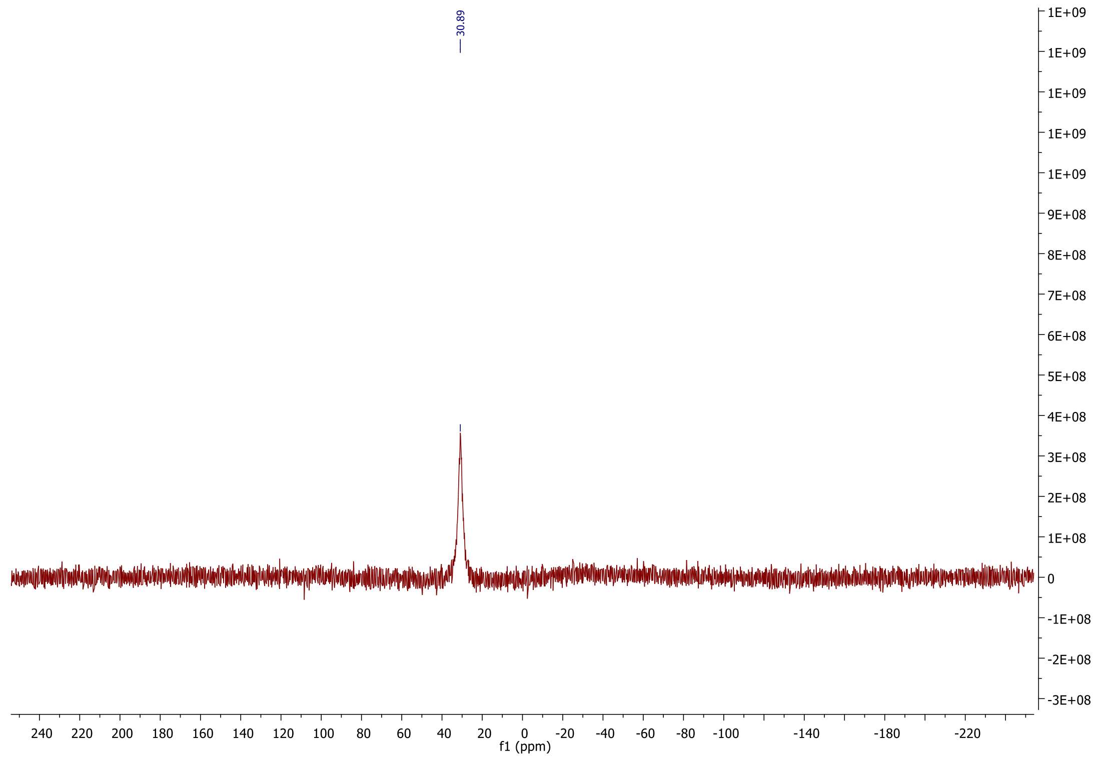
30.89
f1 (ppm)

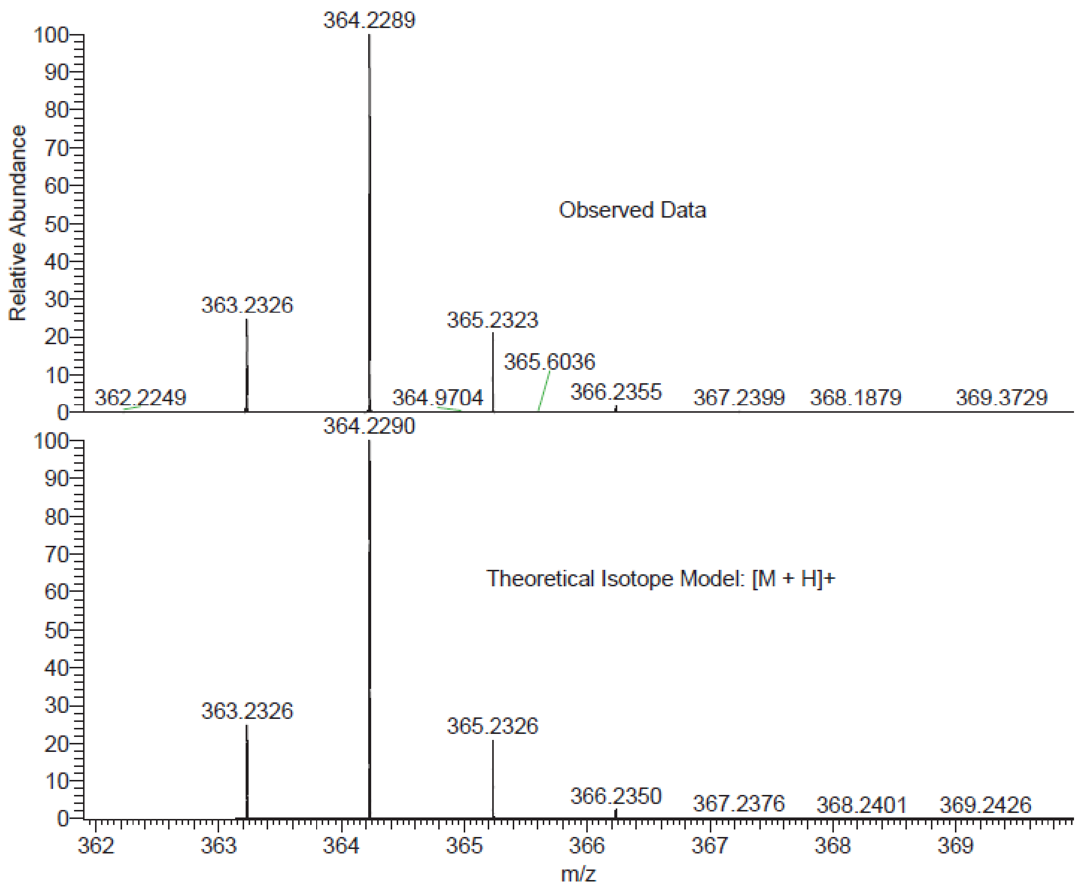

Spectra for intermediate **S5**

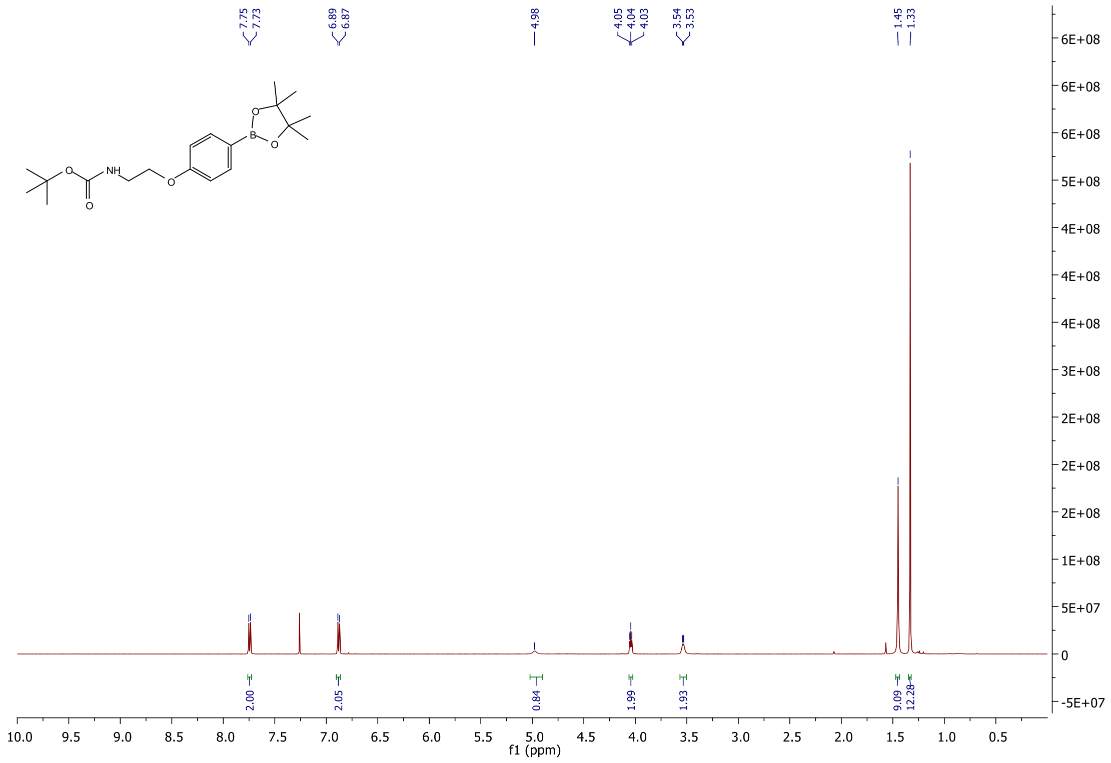

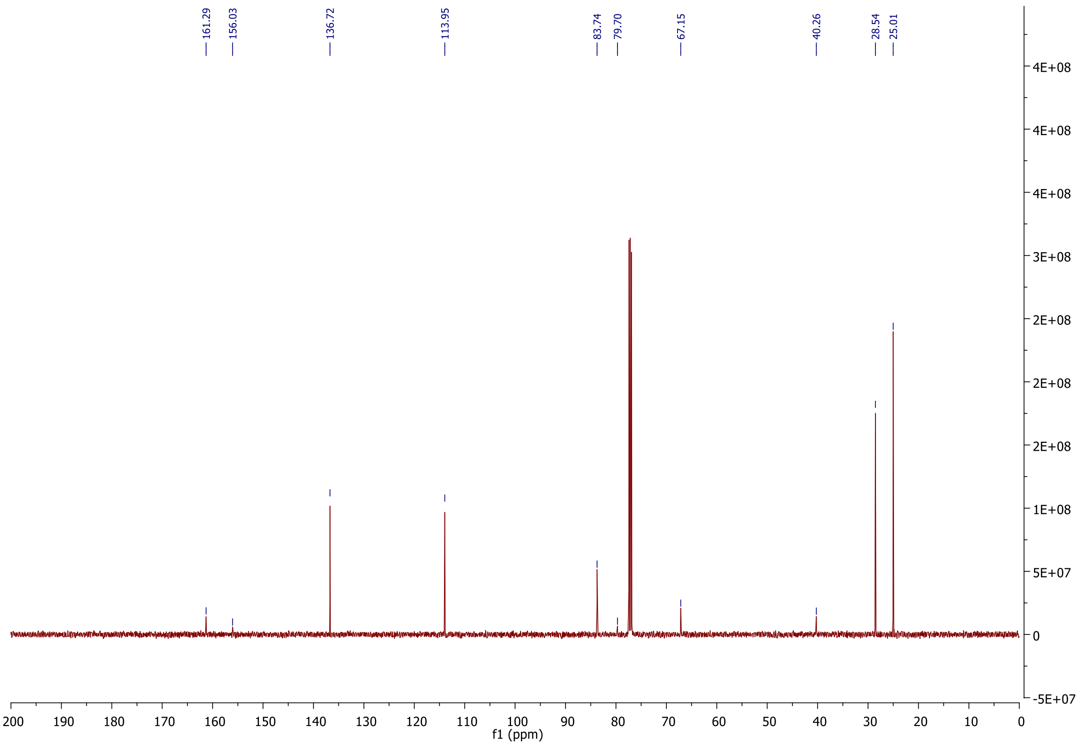

161.29
156.03
136.72
113.95
83.74
79.70
67.15
40.26
28.54
25.01
f1 (ppm)

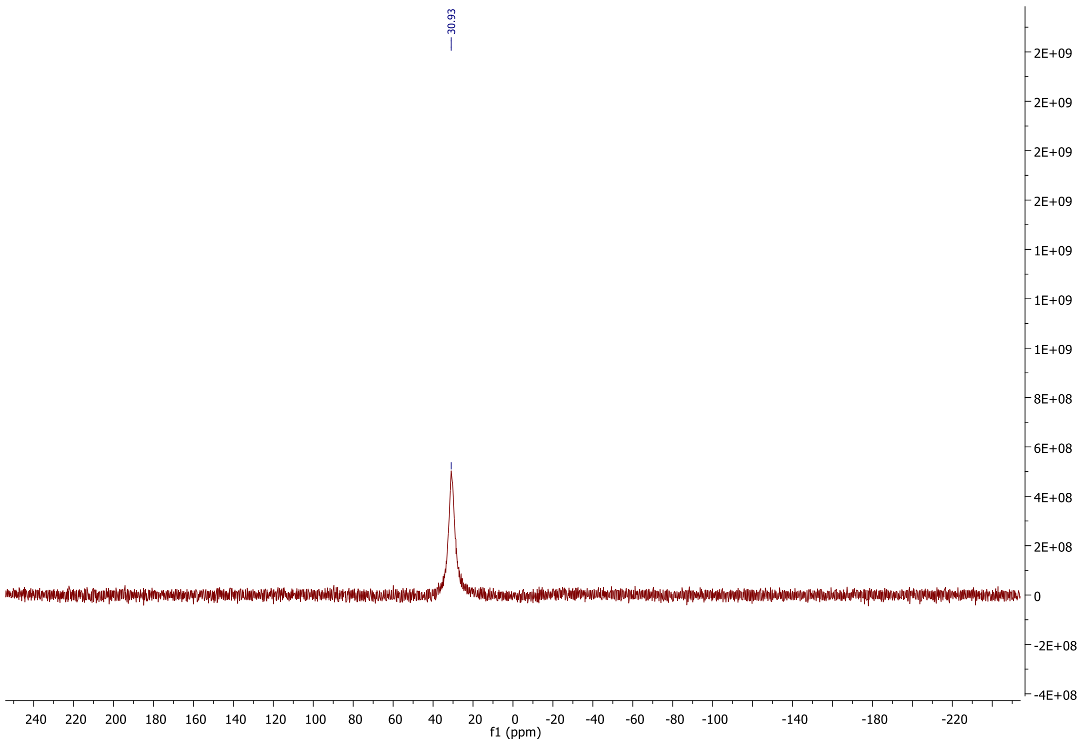

30.93
f1 (ppm)

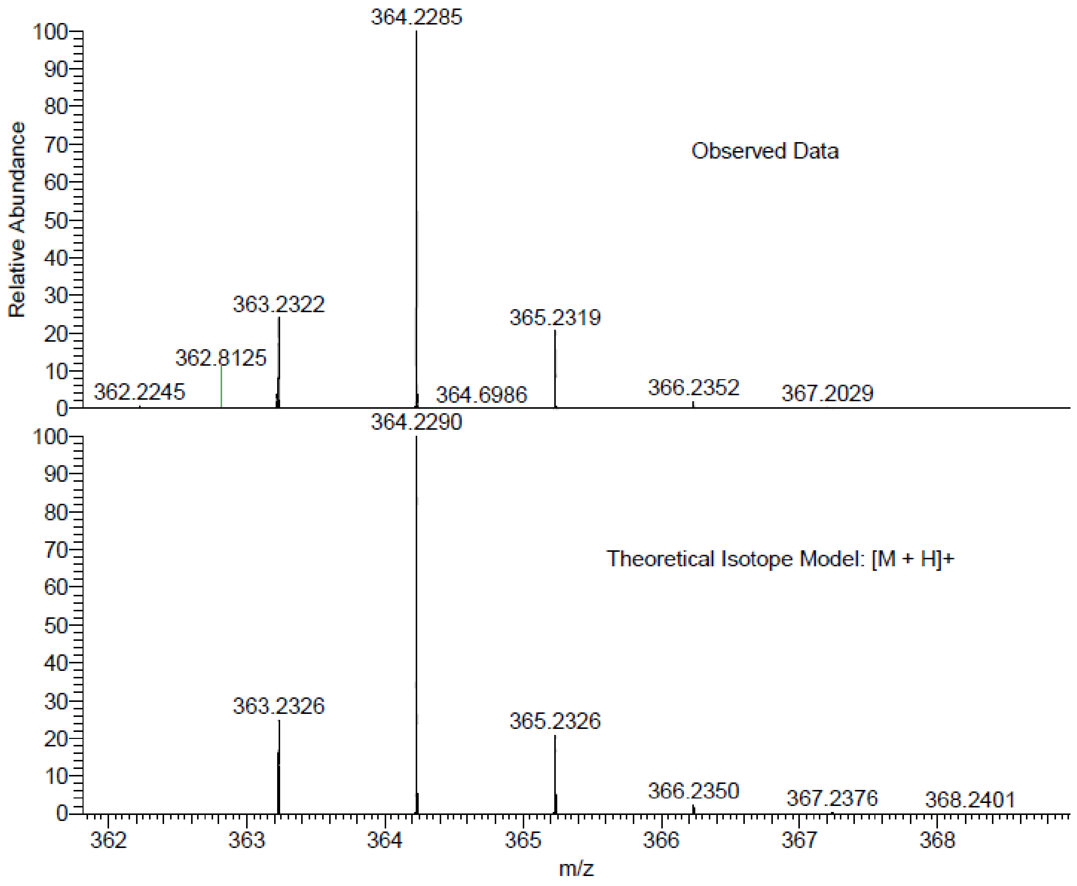

Spectra for intermediate **S6**

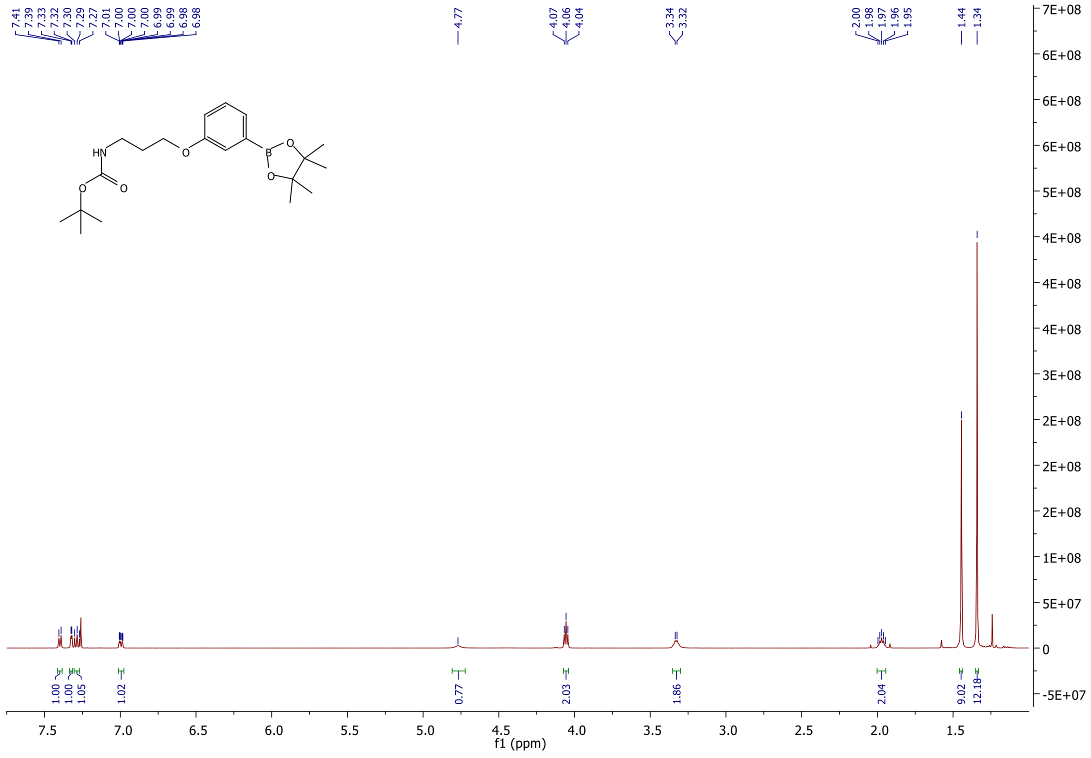

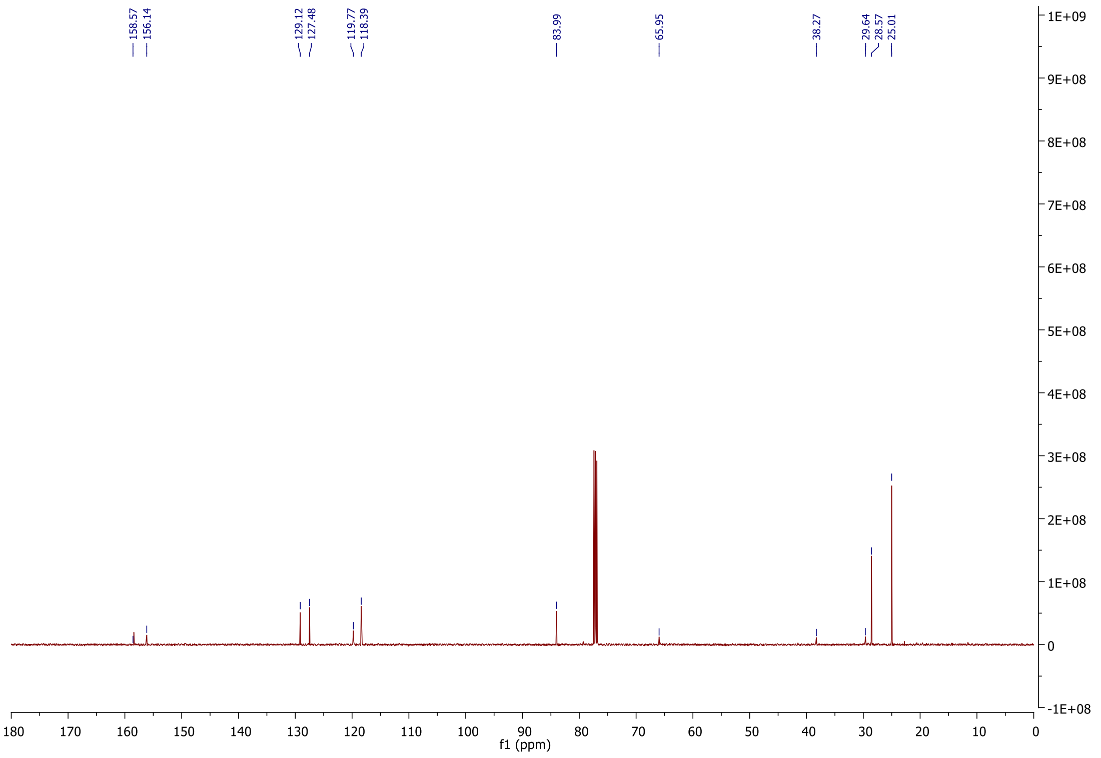
158.57
156.14
129.12
127.48
119.77
118.39
83.99
65.95
38.27
29.64
28.57
25.01
f1 (ppm)

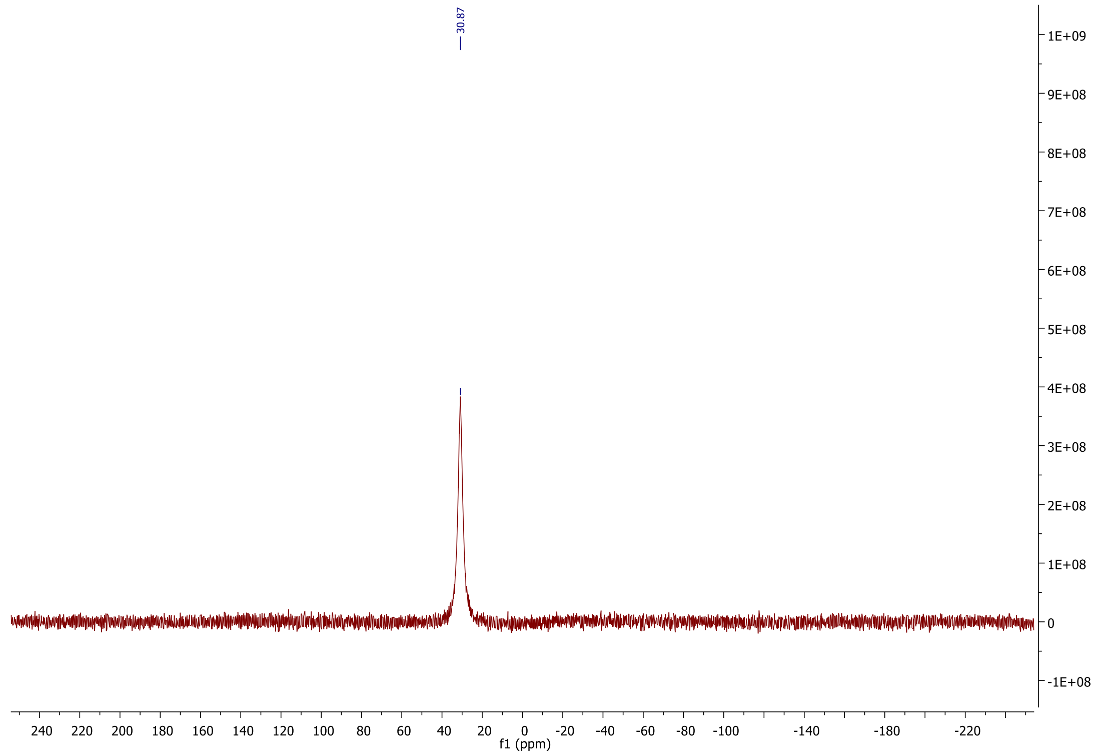
30.87
f1 (ppm)

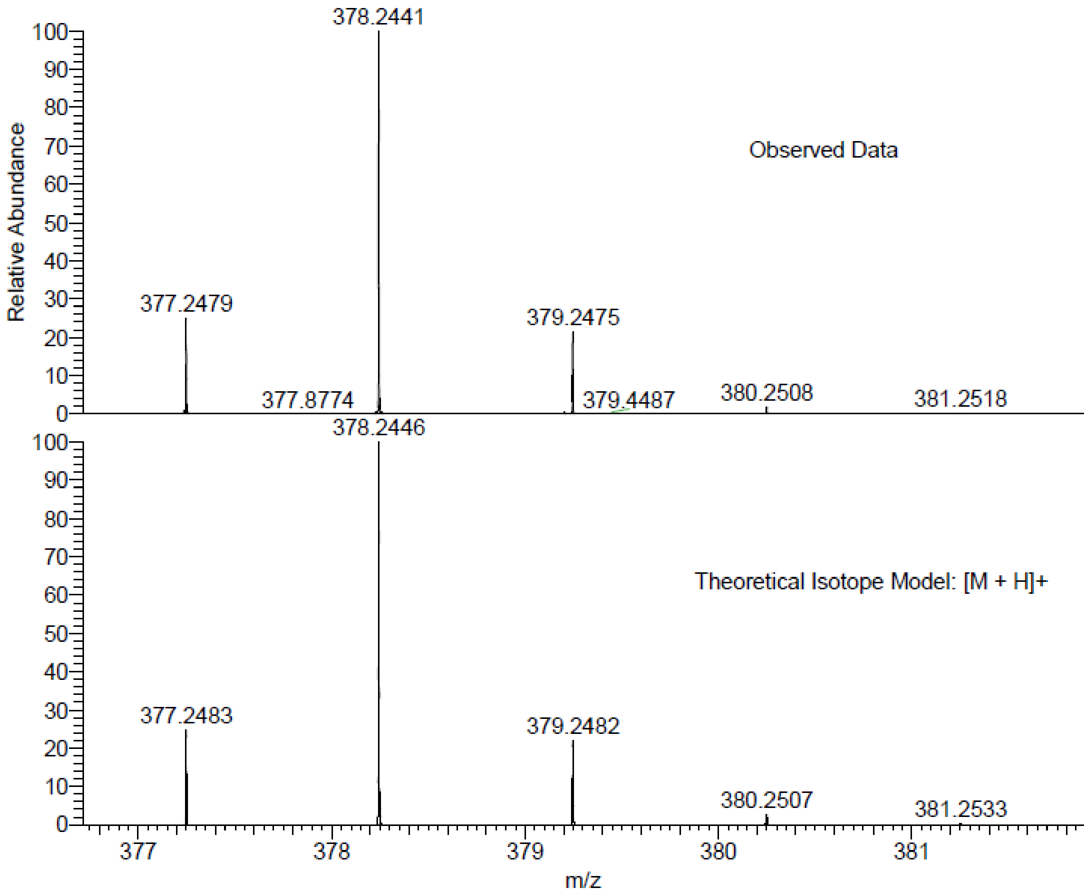

378.2441
Observed Data
377.2479
379.2475
377.8774
379.4487
380.2508
381.2518
378.2446
Theoretical Isotope Model: [M + H]+
377.2483
379.2482
380.2507
381.2533
Relative Abundance
m/z

Spectra for intermediate **S7**

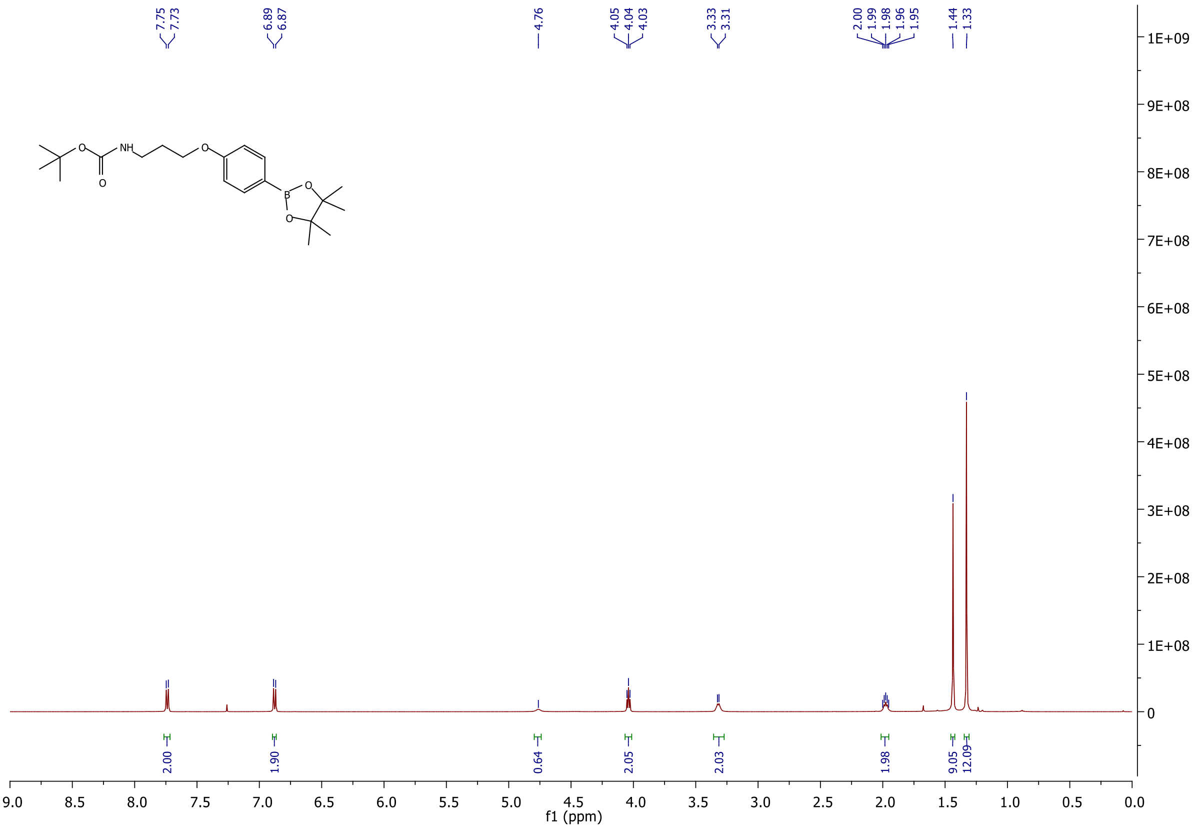

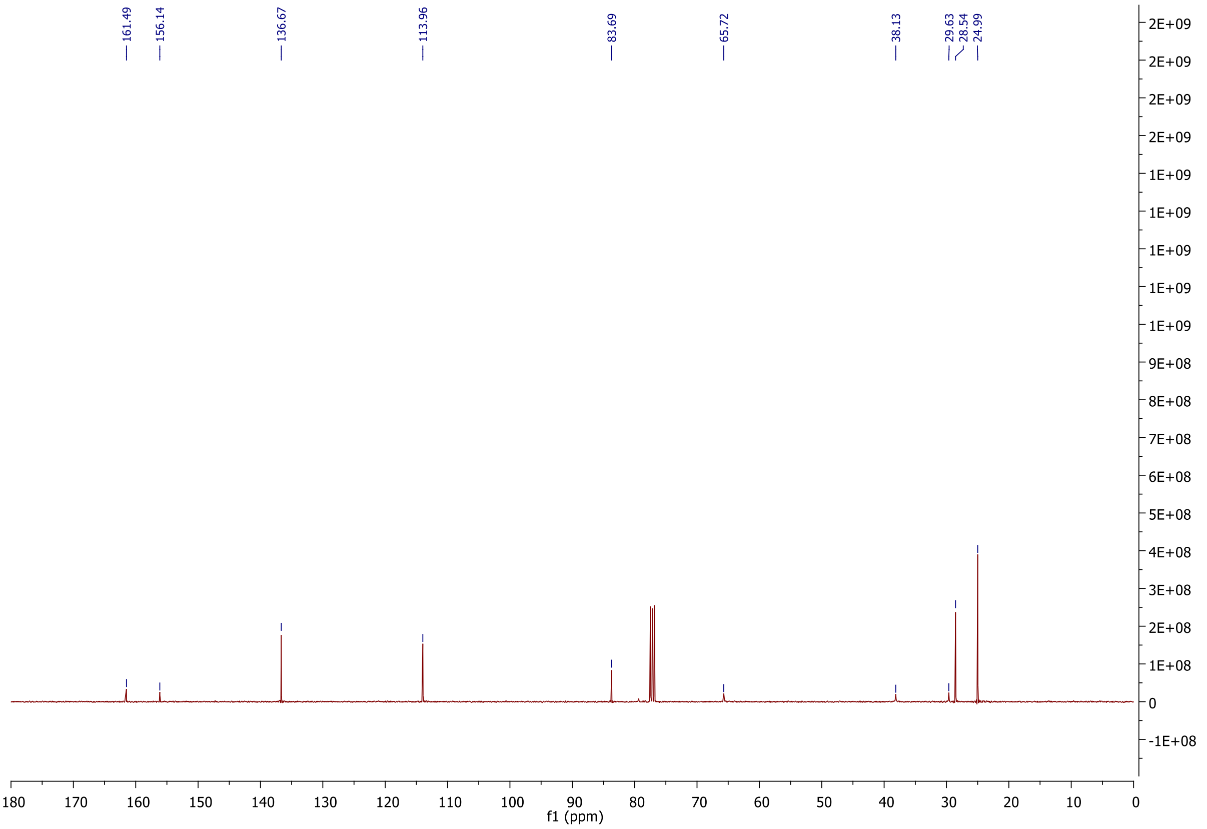

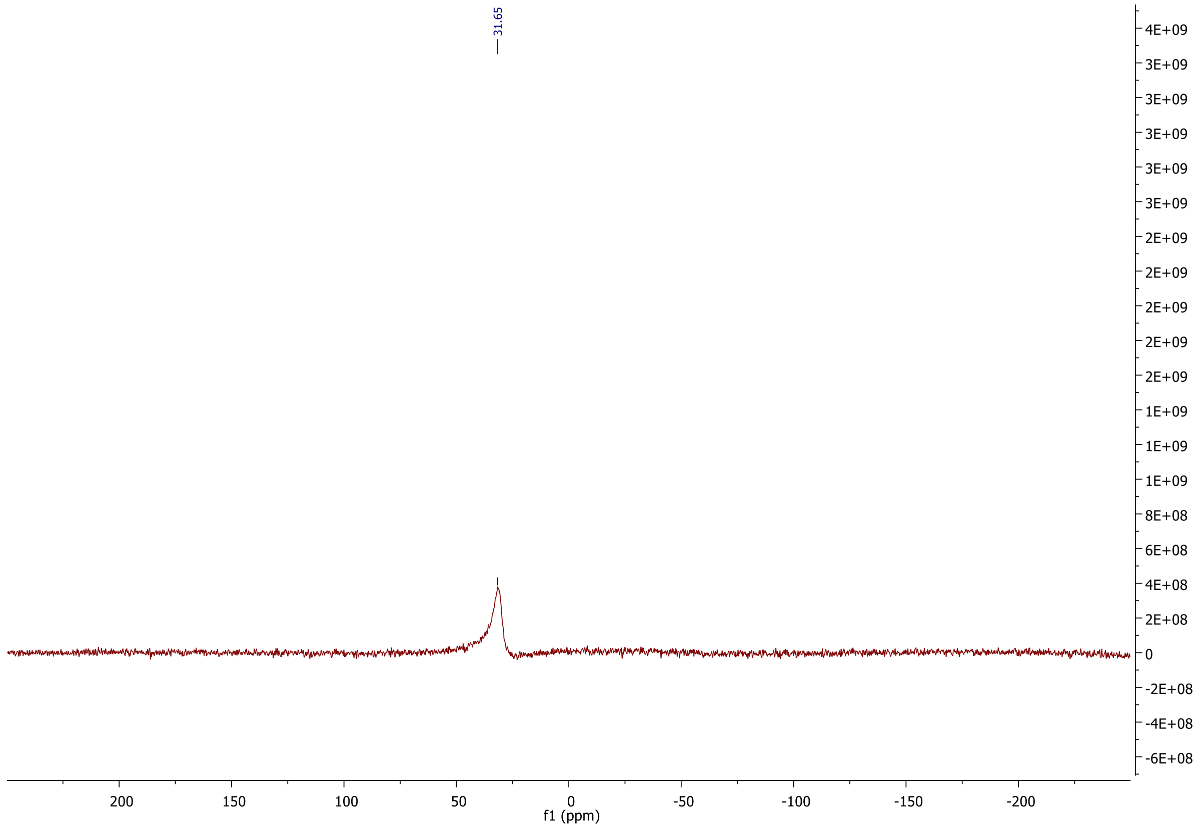
31.65
4E+09
3E+09
3E+09
3E+09
3E+09
3E+09
2E+09
2E+09
2E+09
2E+09
2E+09
1E+09
1E+09
1E+09
8E+08
6E+08
4E+08
2E+08
0
-2E+08
-4E+08
-6E+08
200
150
100
50
0
-50
-100
-150
-200
f1 (ppm)

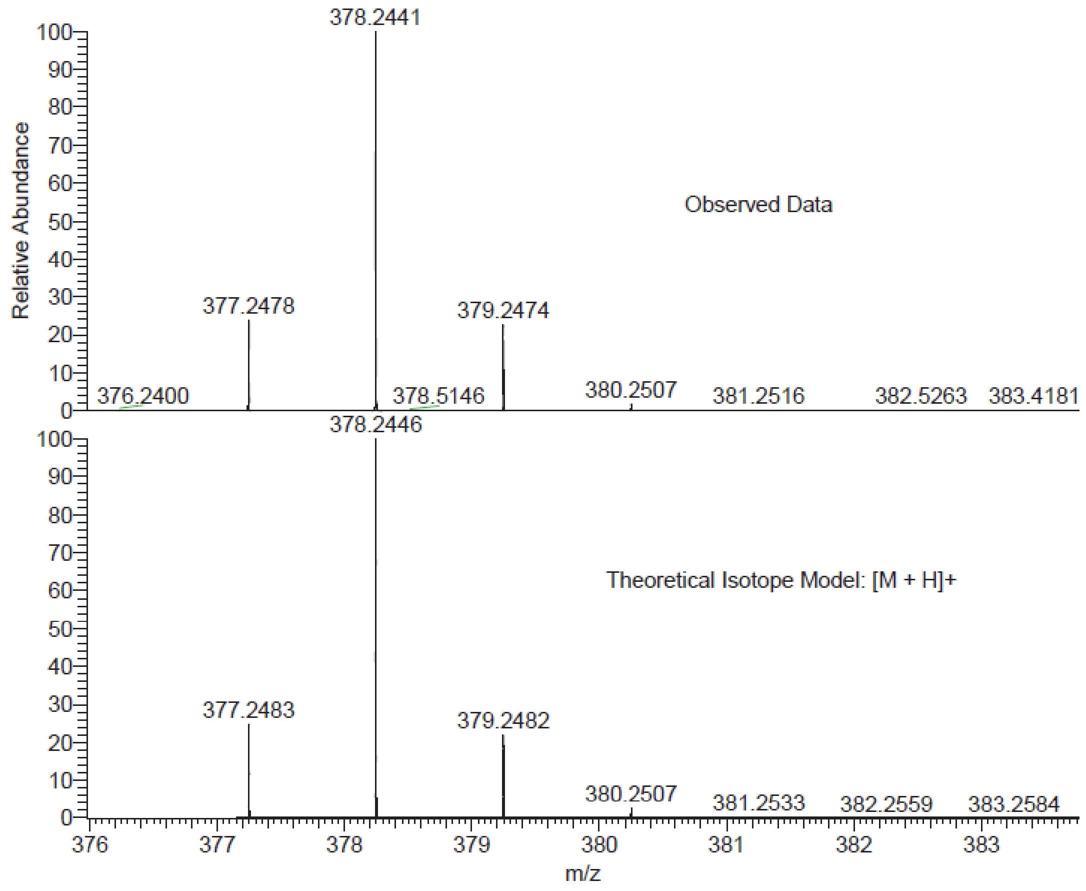

Spectra for intermediate **S8**

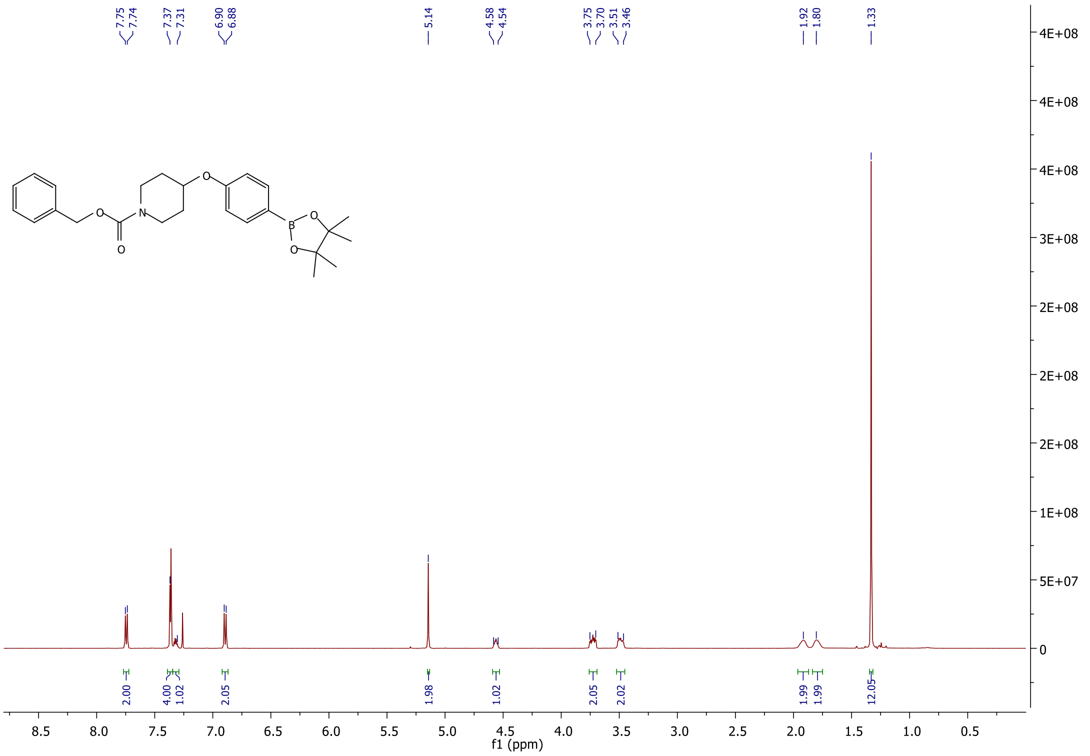

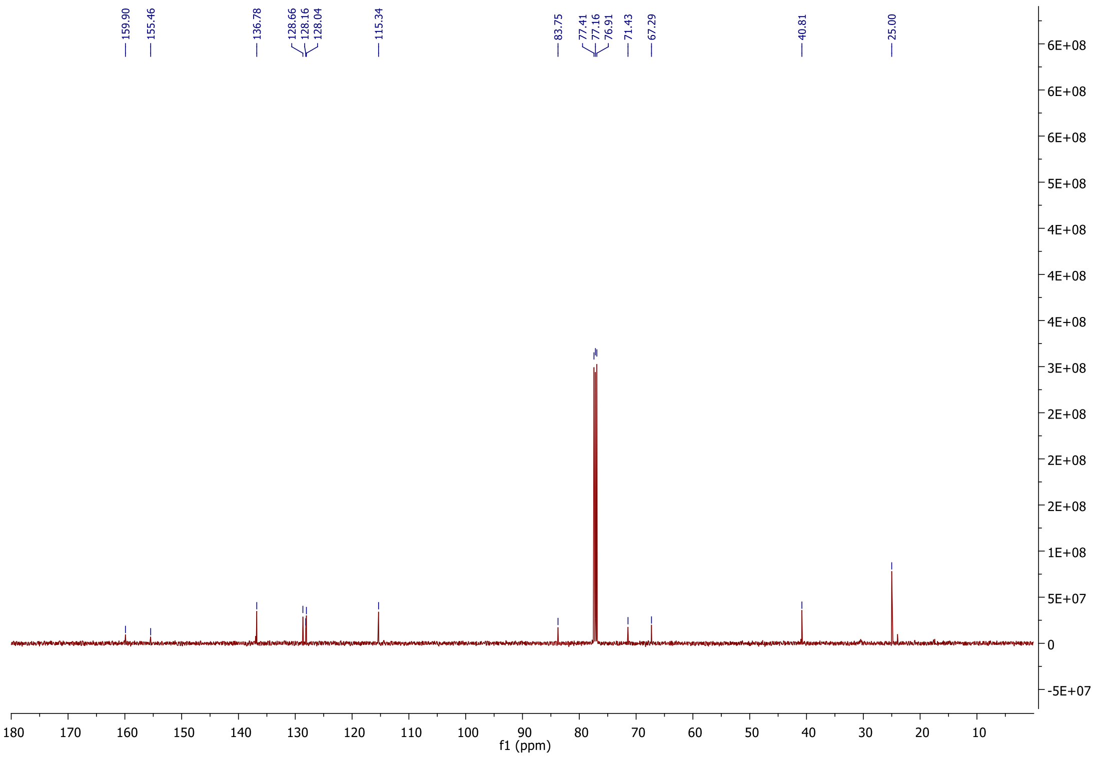
159.90
155.46
136.78
128.66
128.16
128.04
115.34
83.75
77.41
77.16
76.91
71.43
67.29
40.81
25.00
f1 (ppm)

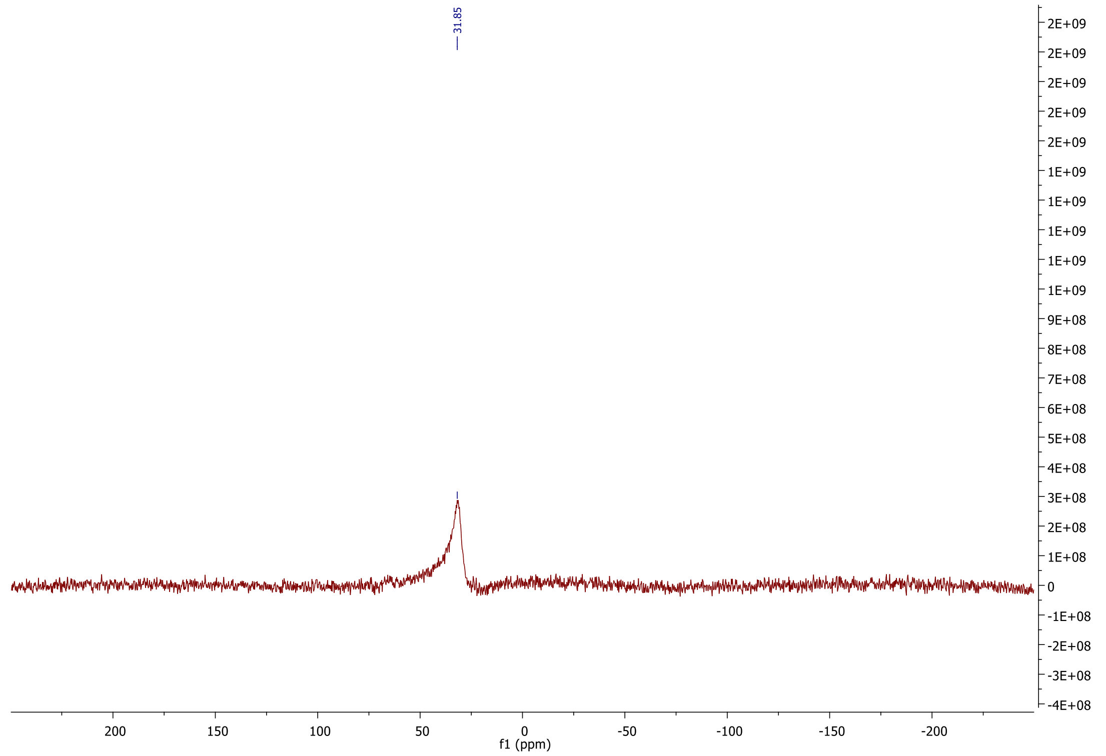
31.85
f1 (ppm)

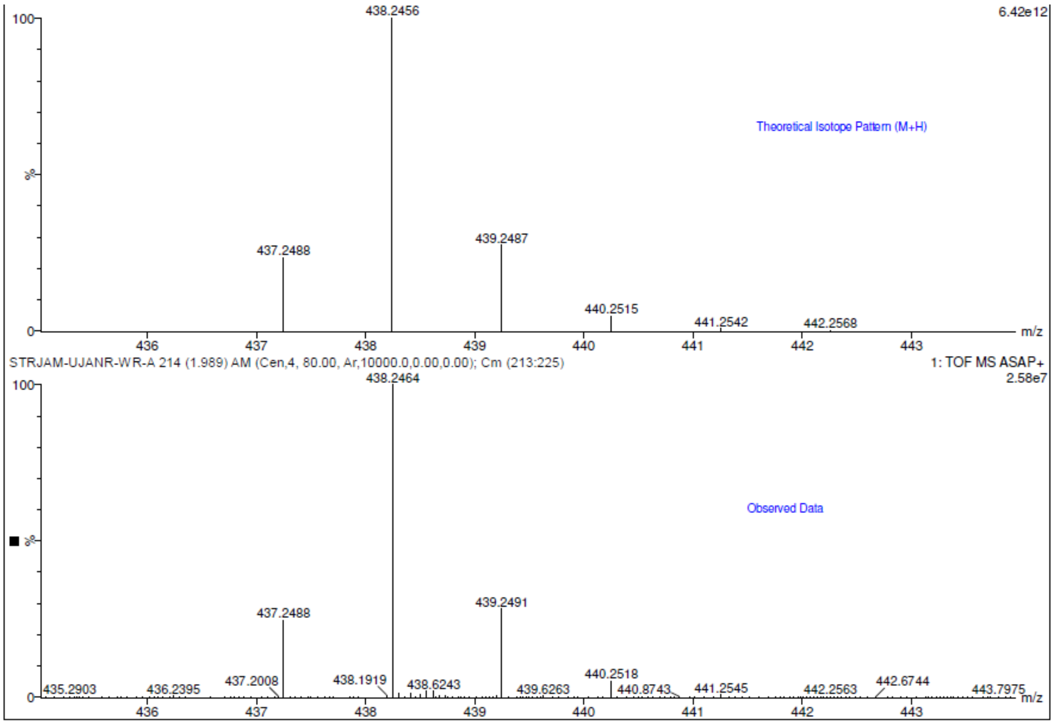

Theoretical Isotope Pattern (M+H)
6.42e12
438.2456
437.2488
439.2487
440.2515
441.2542
442.2568
STRJAM-UJANR-WR-A 214 (1.989) AM (Cen,4, 80.00, Ar,10000.0,0.00,0.00); Cm (213:225)
1: TOF MS ASAP+
2.58e7
Observed Data
438.2464
437.2488
439.2491
435.2903
436.2395
437.2008
438.1919
438.6243
439.6263
440.2518
440.8743
441.2545
442.2563
442.6744
443.7975
m/z
%

Spectra for intermediate **S9**

^{1}H at 300K

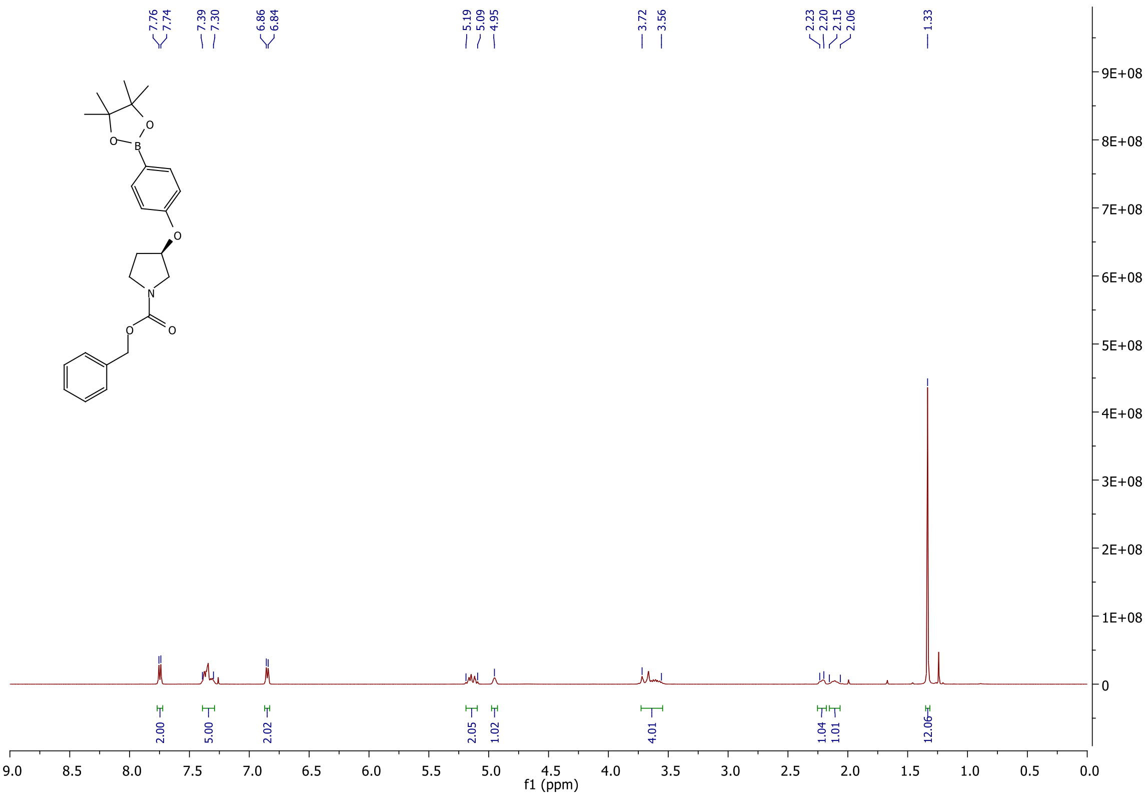

^{13}C at 300K

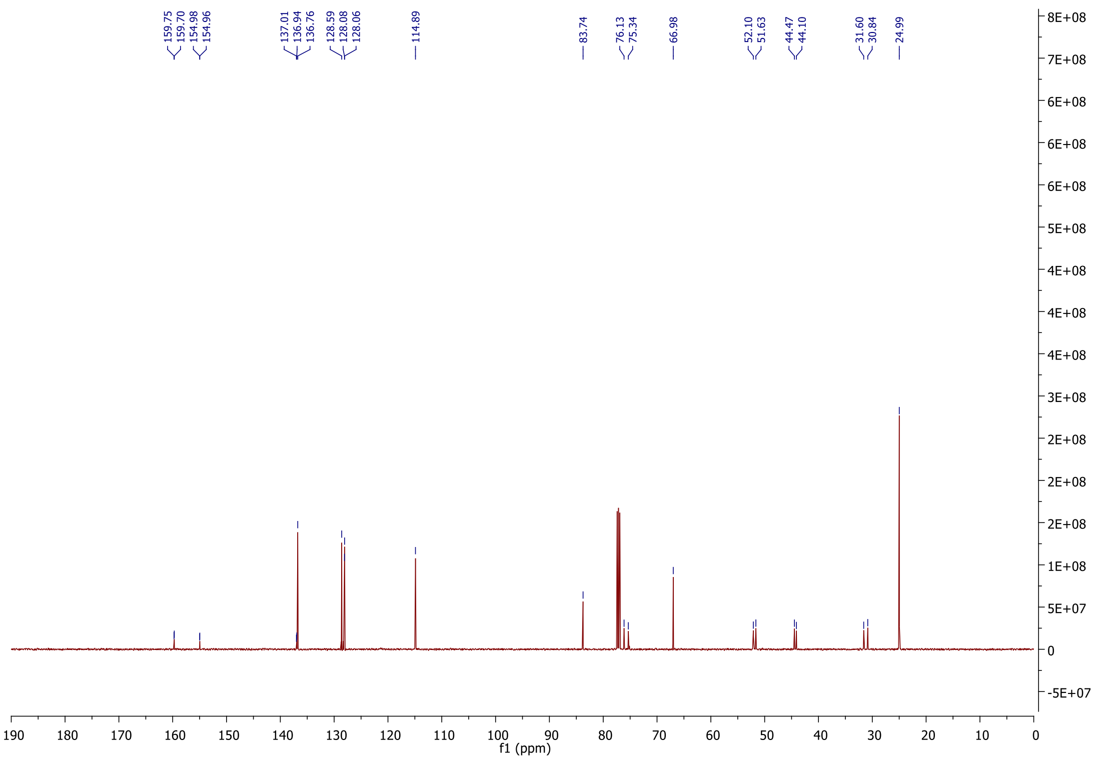

^{1}H at 373K

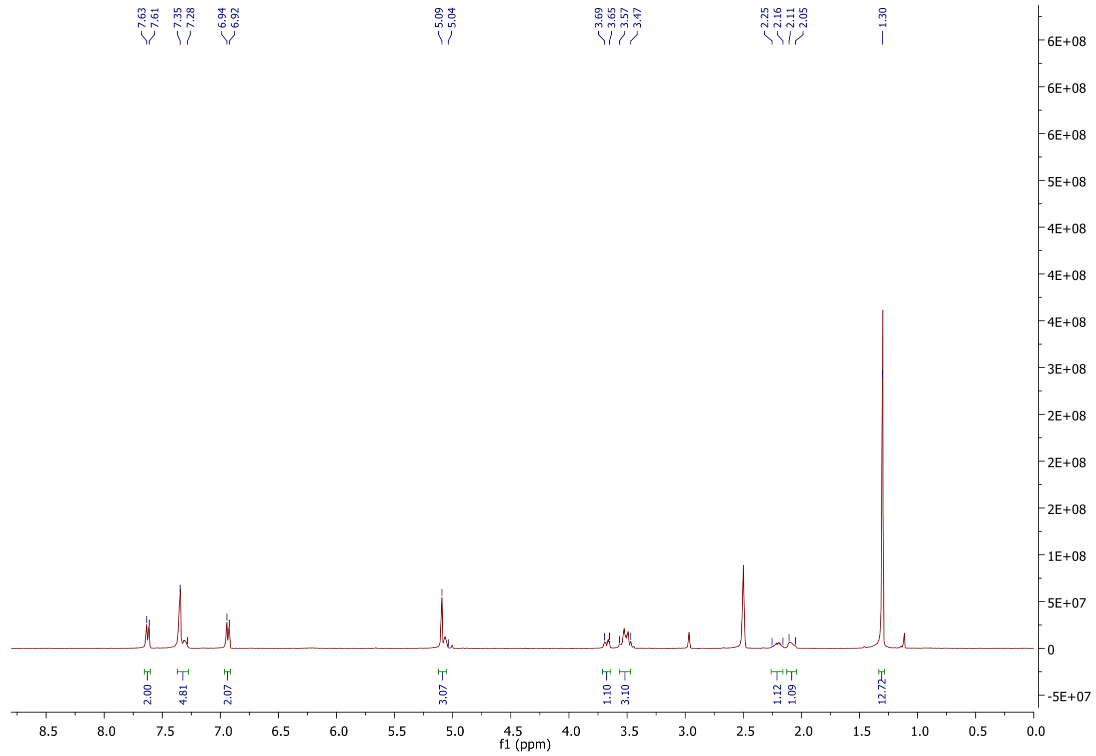

^{13}C at 373K

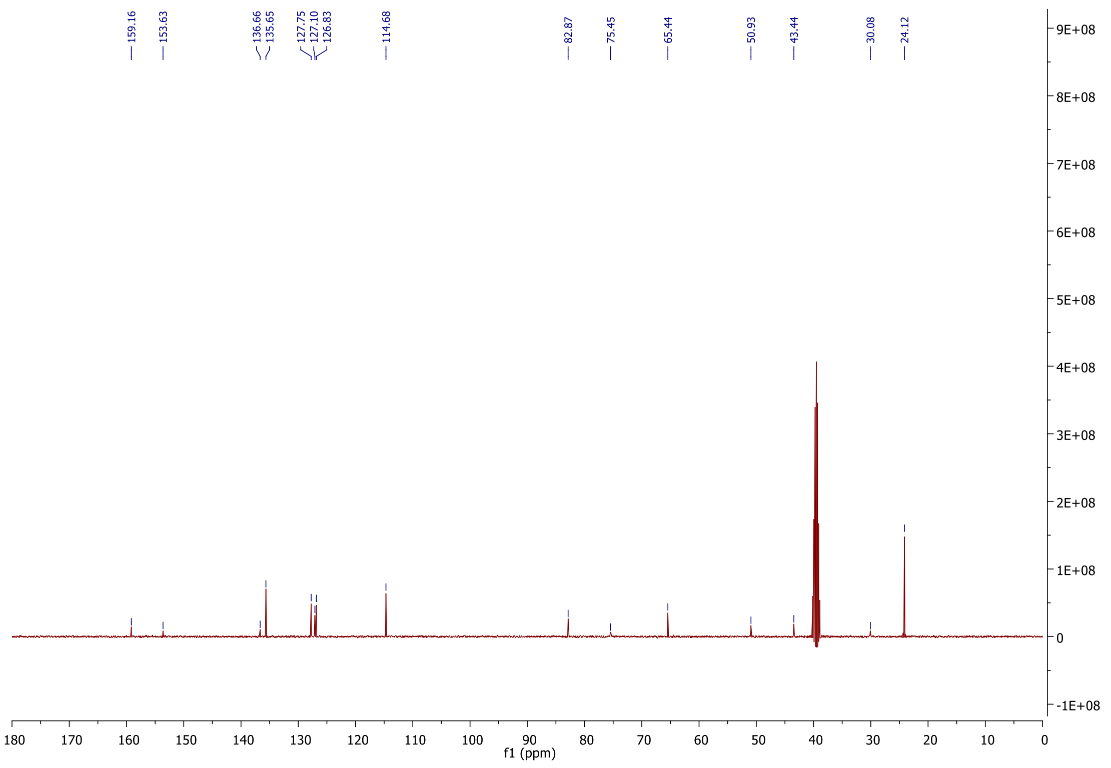

^{11}B

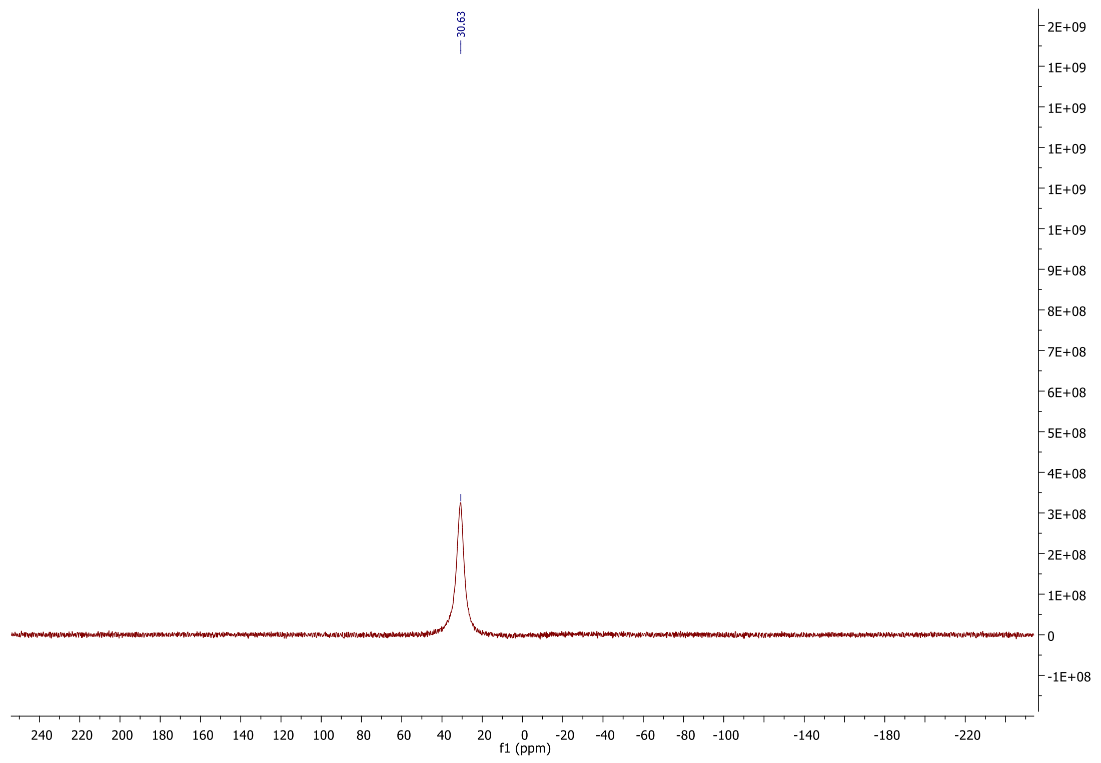

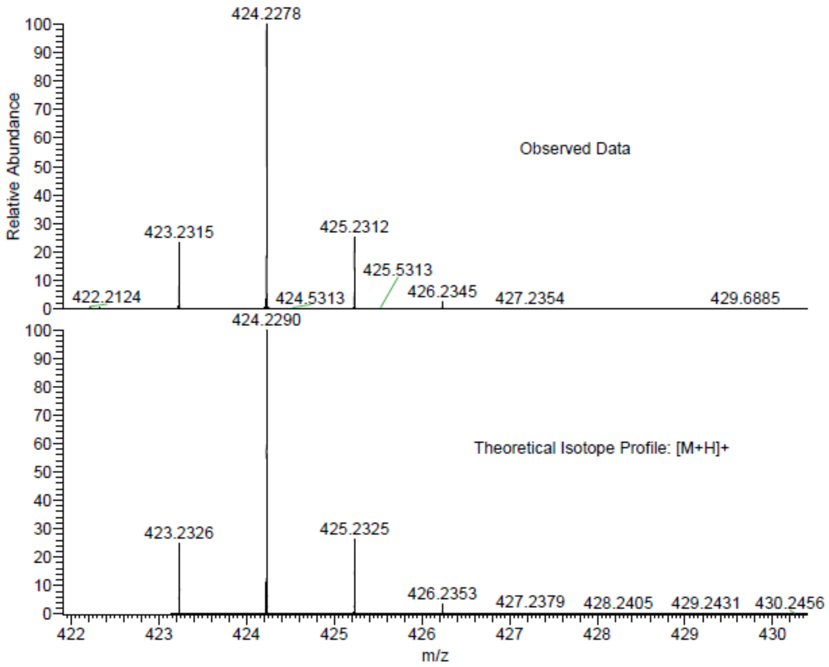

Relative Abundance
424.2278
Observed Data
423.2315
425.2312
425.5313
426.2345
422.2124
424.5313
427.2354
429.6885
424.2290
Theoretical Isotope Profile: [M+H]+
423.2326
425.2325
426.2353
427.2379
428.2405
429.2431
430.2456
m/z

Spectra for intermediate **S10**

^{1}H at 373K

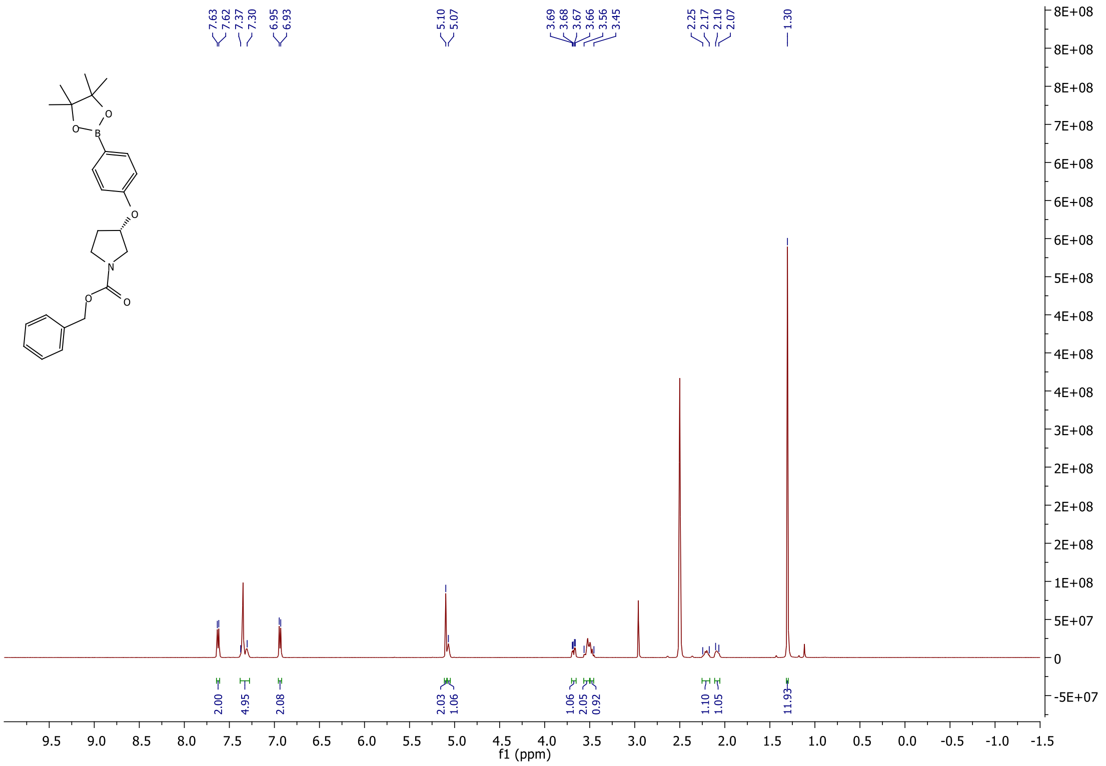

^{13}C at 373K

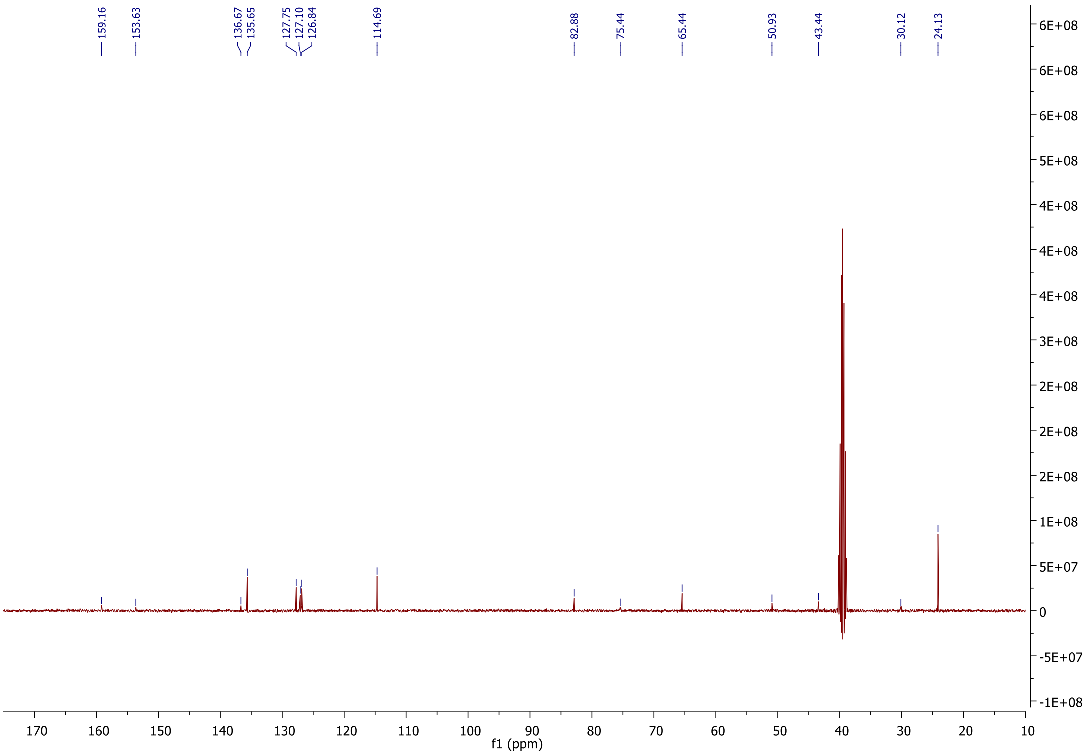
159.16
153.63
136.67
135.65
127.75
127.10
126.84
114.69
82.88
75.44
65.44
50.93
43.44
30.12
24.13
f1 (ppm)

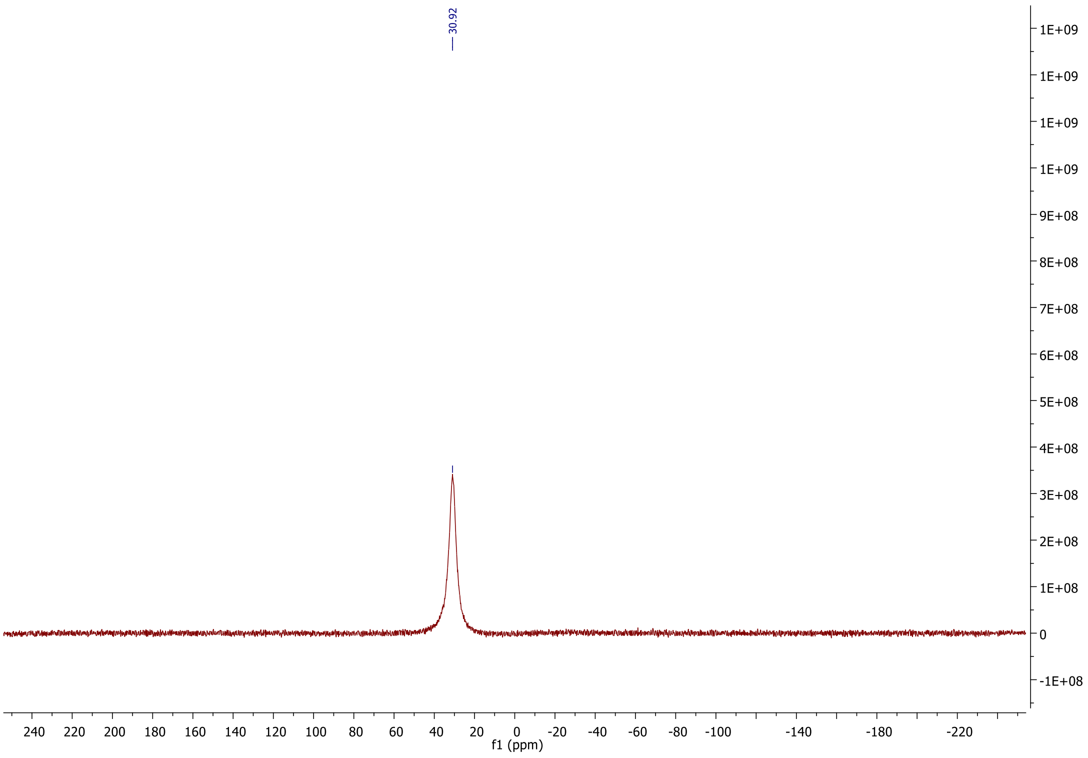
30.92
f1 (ppm)

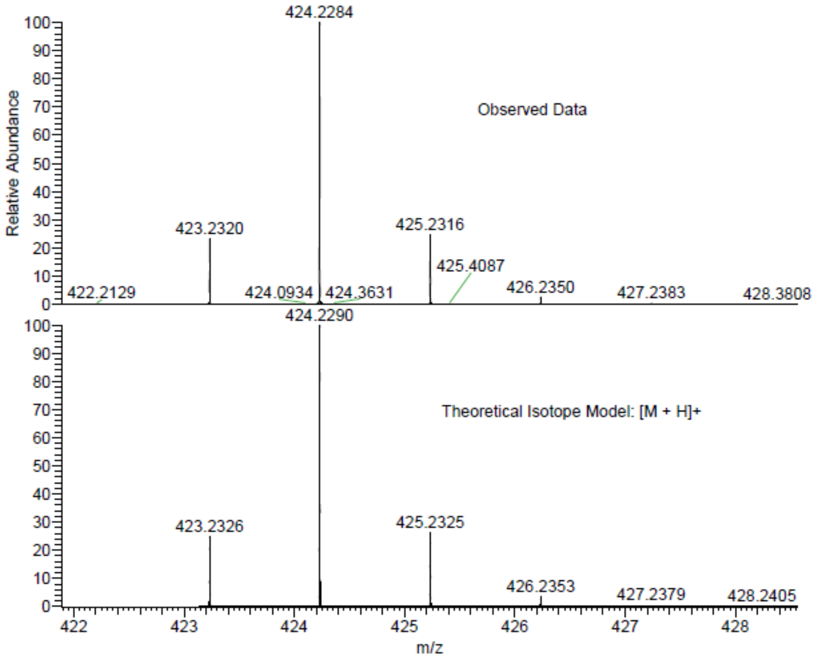

Spectra for **10**

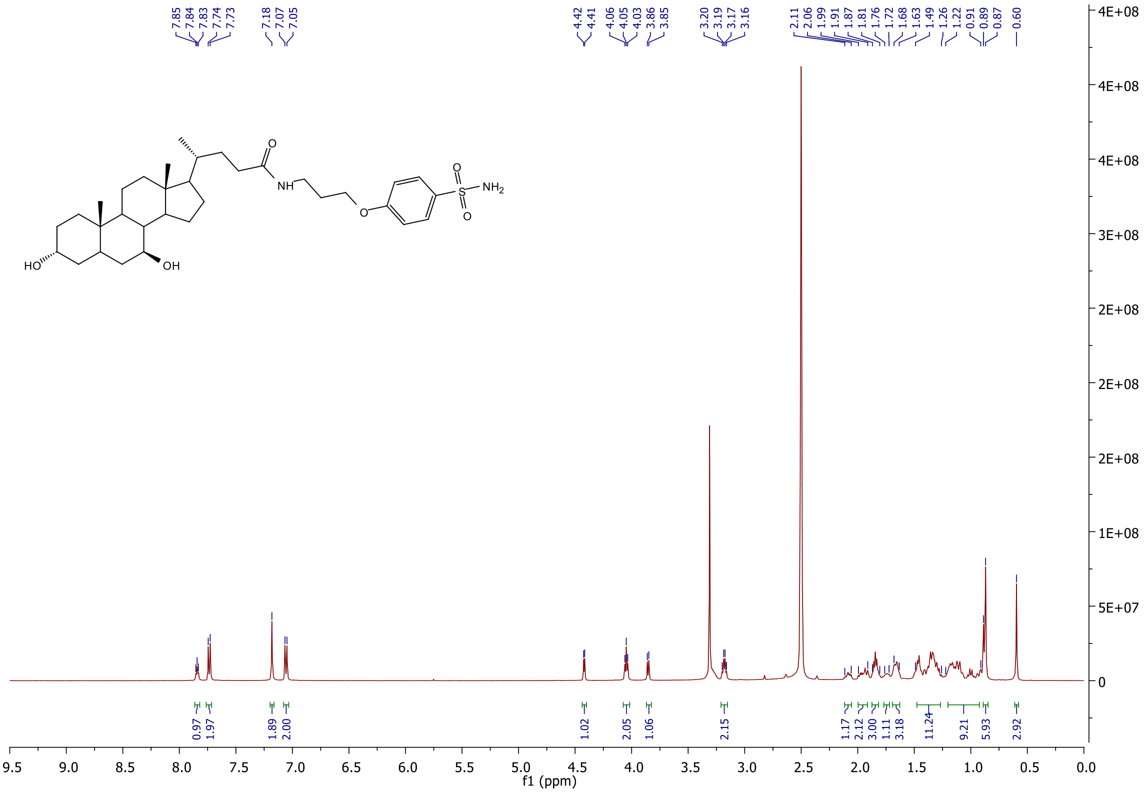

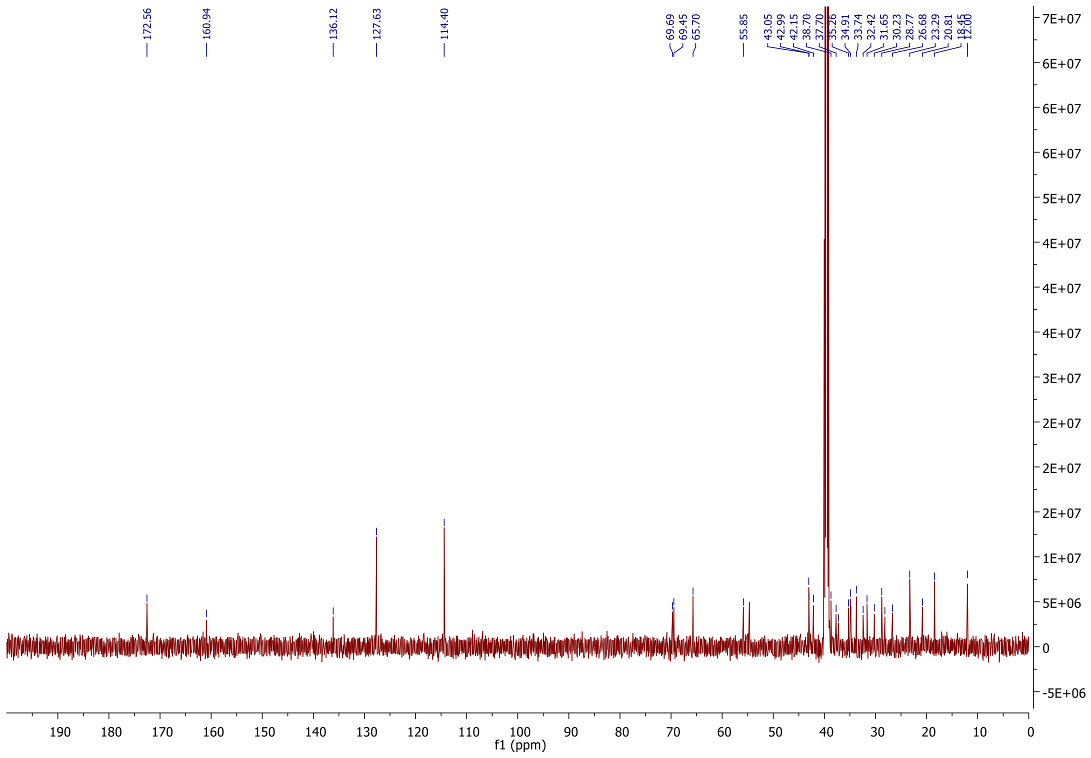
172.56
160.94
136.12
127.63
114.40
69.69
69.45
65.70
55.85
43.05
42.99
42.15
38.70
37.70
35.26
34.91
33.74
32.42
31.65
30.23
28.77
26.68
23.29
20.81
18.45
12.00
7E+07
6E+07
6E+07
6E+07
5E+07
4E+07
4E+07
4E+07
3E+07
2E+07
2E+07
2E+07
1E+07
5E+06
0
-5E+06
190 180 170 160 150 140 130 120 110 100 90 80 70 60 50 40 30 20 10 0
f1 (ppm)

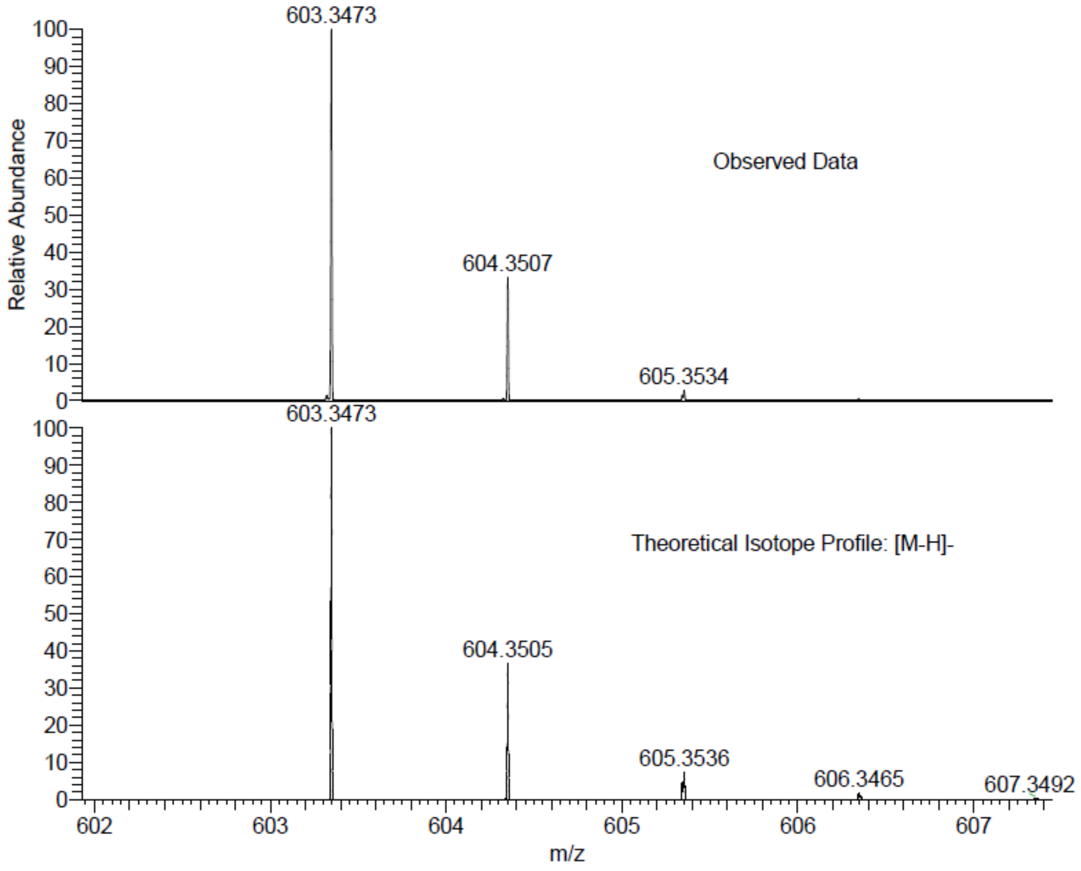
603.3473
604.3507
605.3534
Observed Data
603.3473
604.3505
605.3536
606.3465
607.3492
Theoretical Isotope Profile: [M-H]-
Relative Abundance
100 90 80 70 60 50 40 30 20 10 0
602 603 604 605 606 607
m/z

Spectra for **11**

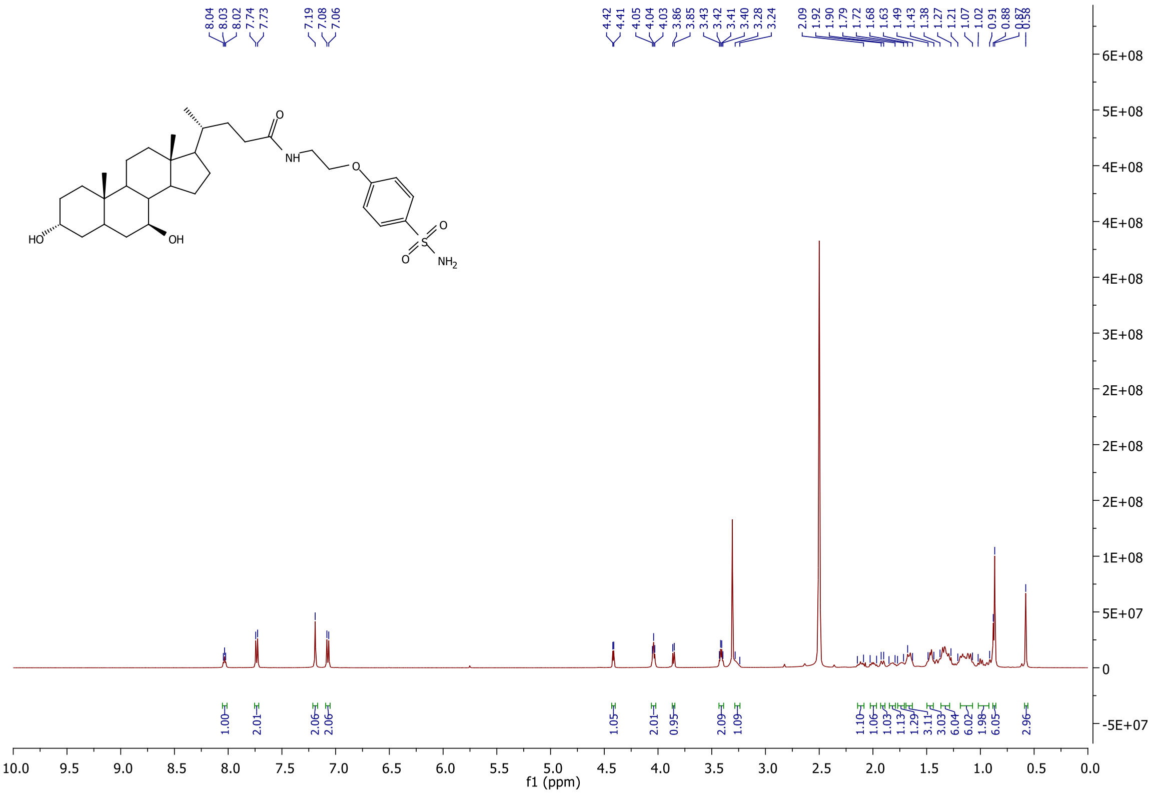

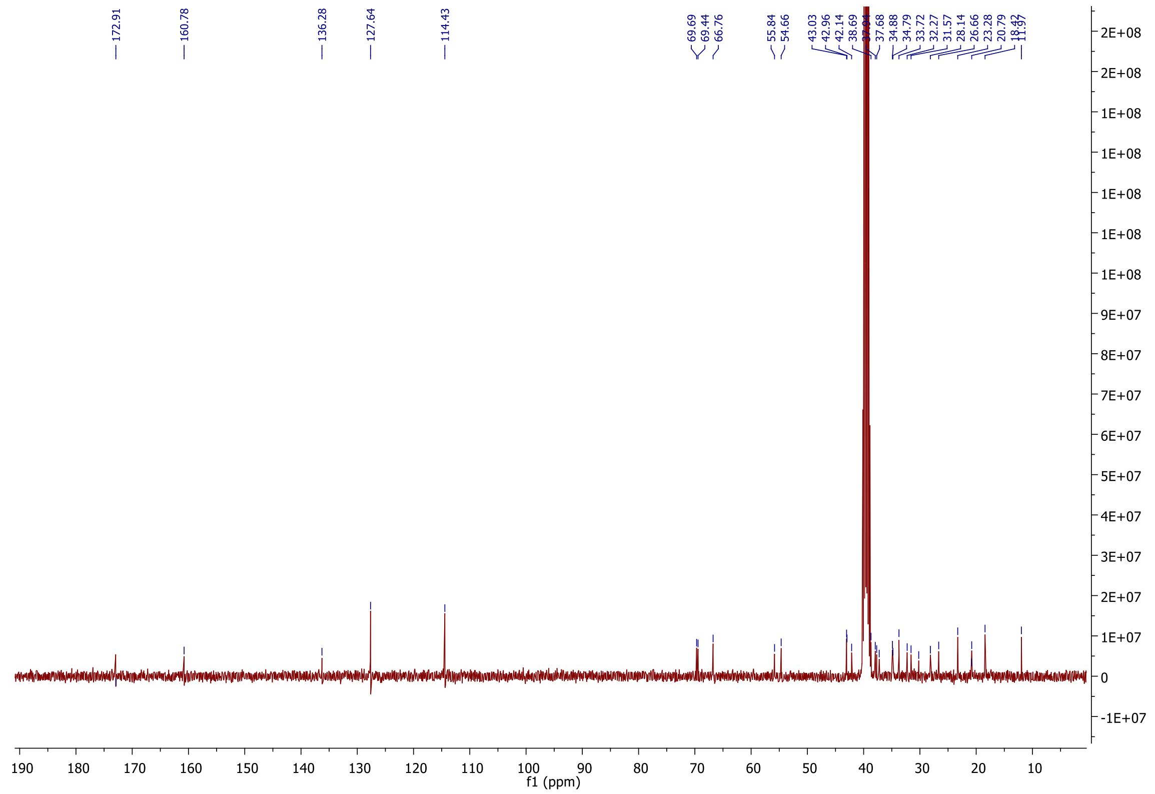

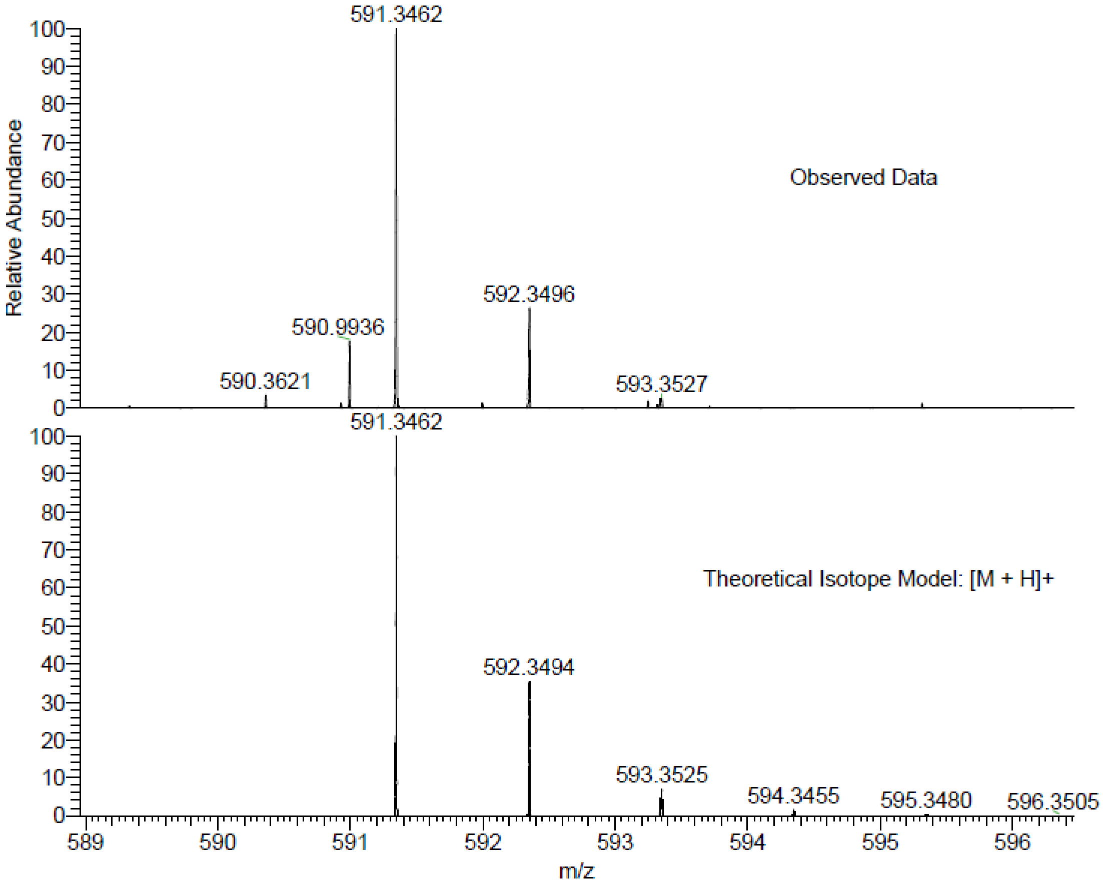

Spectra for **12**

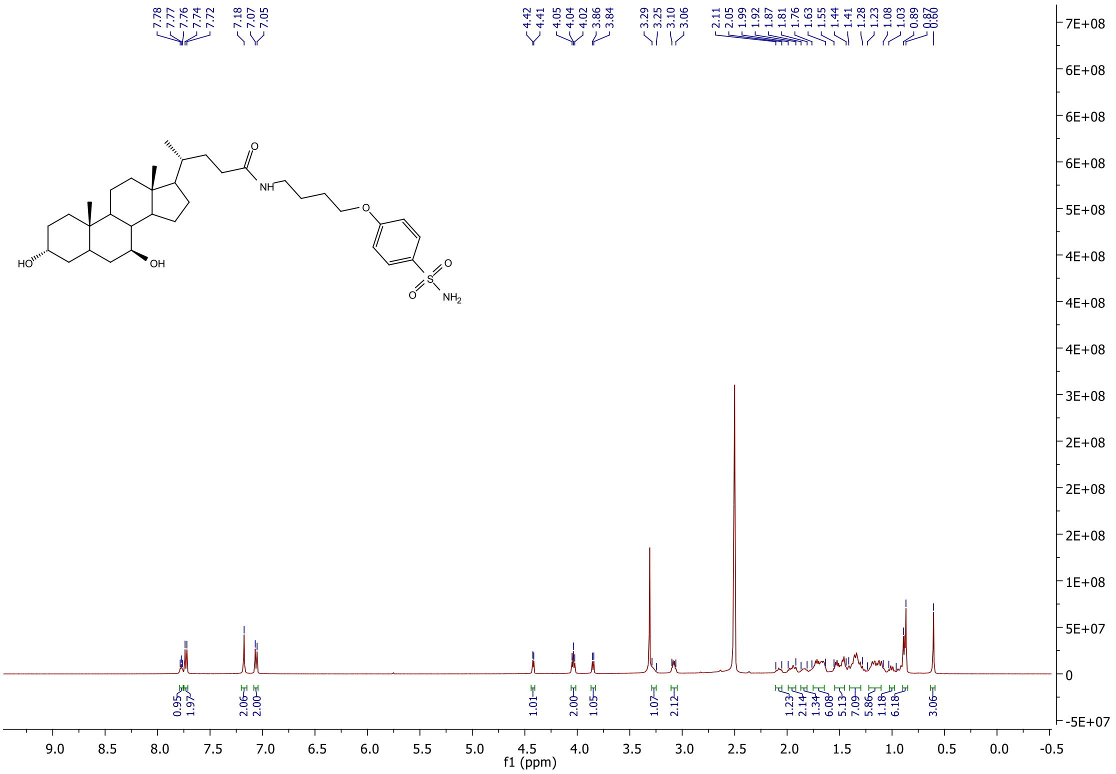

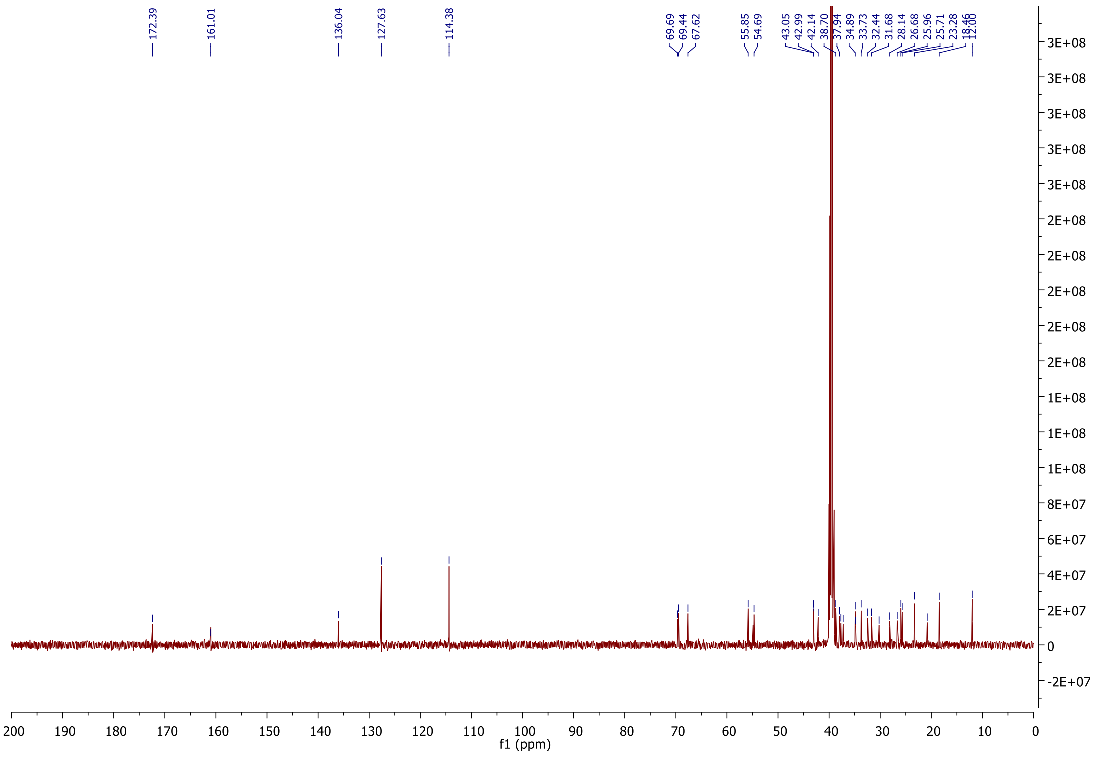

172.39
161.01
136.04
127.63
114.38
69.69
69.44
67.62
55.85
54.69
43.05
42.99
42.14
38.70
37.94
34.89
33.73
32.44
31.68
28.14
26.68
25.96
25.71
23.28
18.46
12.00
3E+08
3E+08
3E+08
3E+08
3E+08
2E+08
2E+08
2E+08
2E+08
2E+08
1E+08
1E+08
1E+08
8E+07
6E+07
4E+07
2E+07
0
-2E+07
200
190
180
170
160
150
140
130
120
110
100
90
80
70
60
50
40
30
20
10
0
f1 (ppm)

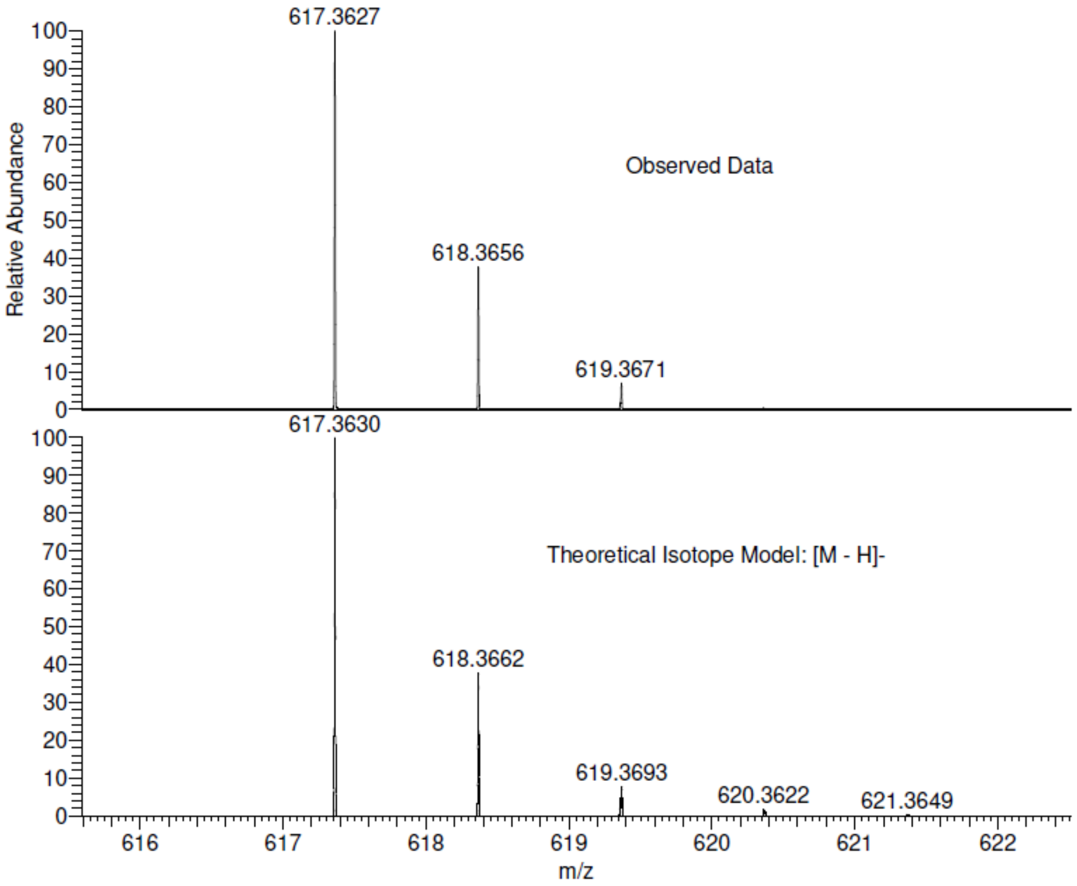

617.3627
Observed Data
618.3656
619.3671
617.3630
Theoretical Isotope Model: [M - H]-
618.3662
619.3693
620.3622
621.3649
Relative Abundance
100
90
80
70
60
50
40
30
20
10
0
616
617
618
619
620
621
622
m/z

Spectra for **13**

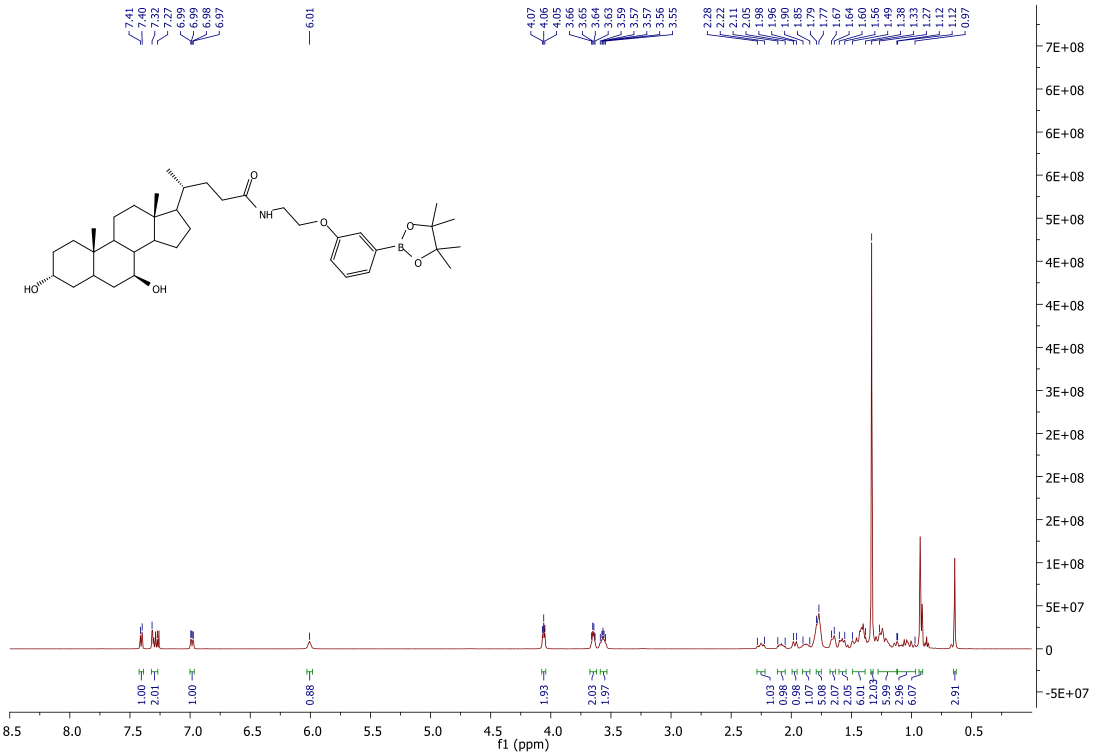

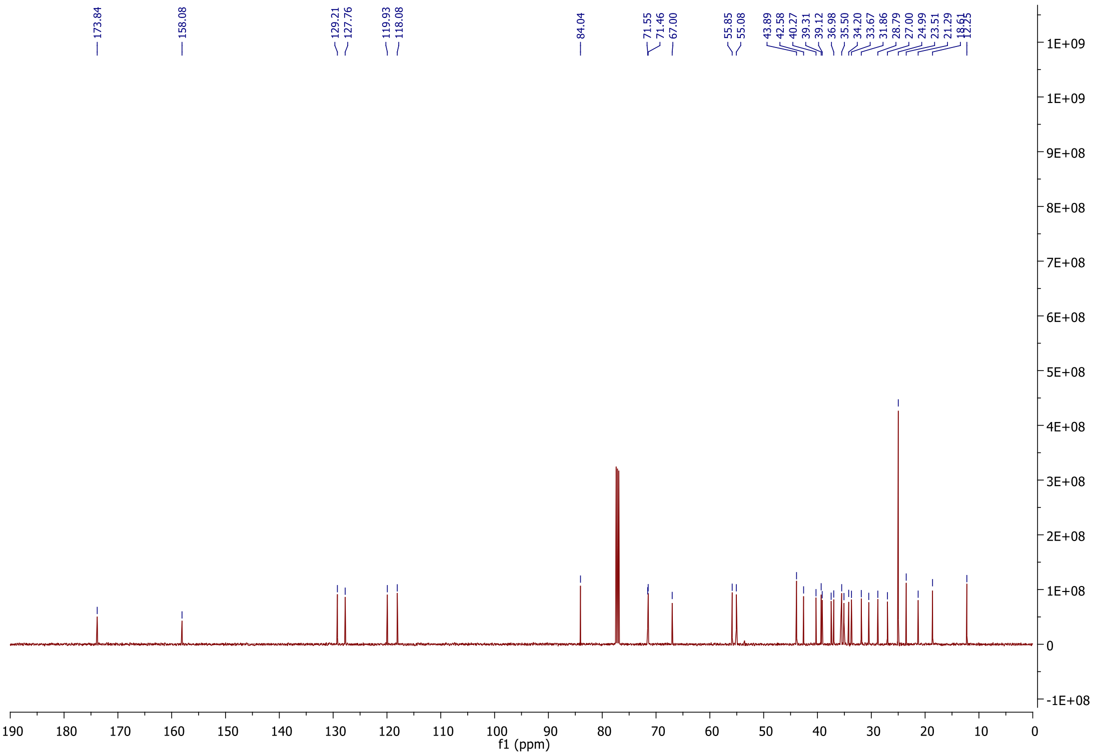

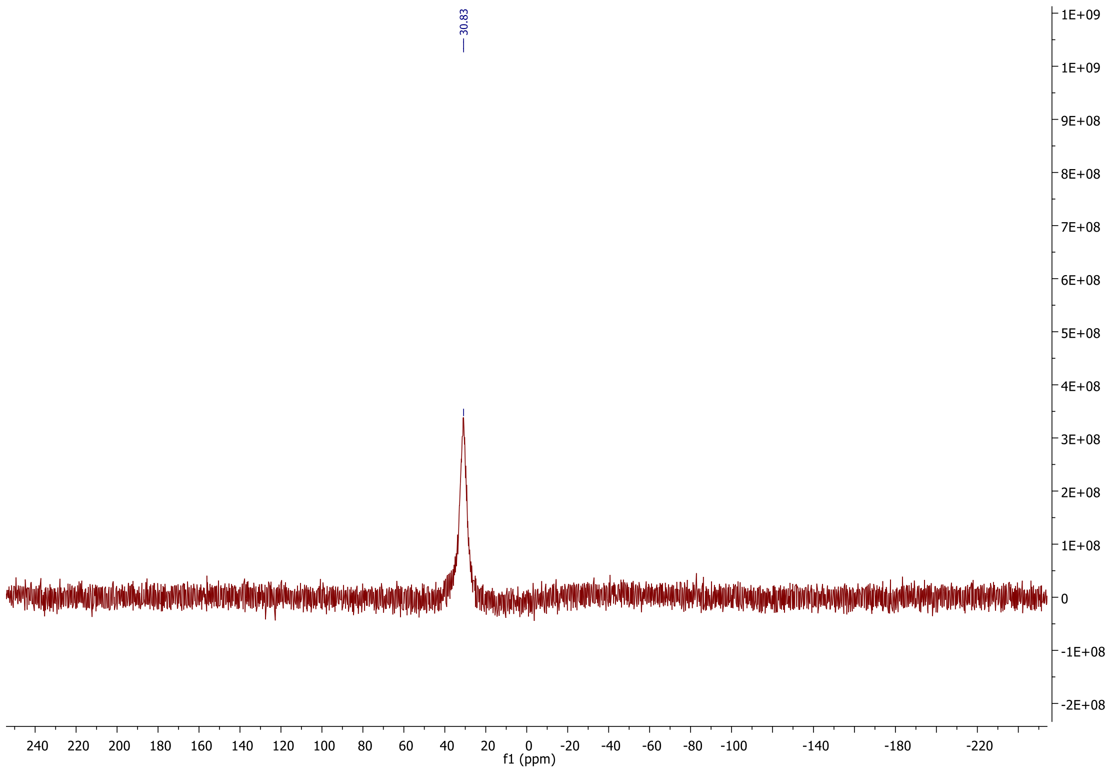
30.83
f1 (ppm)

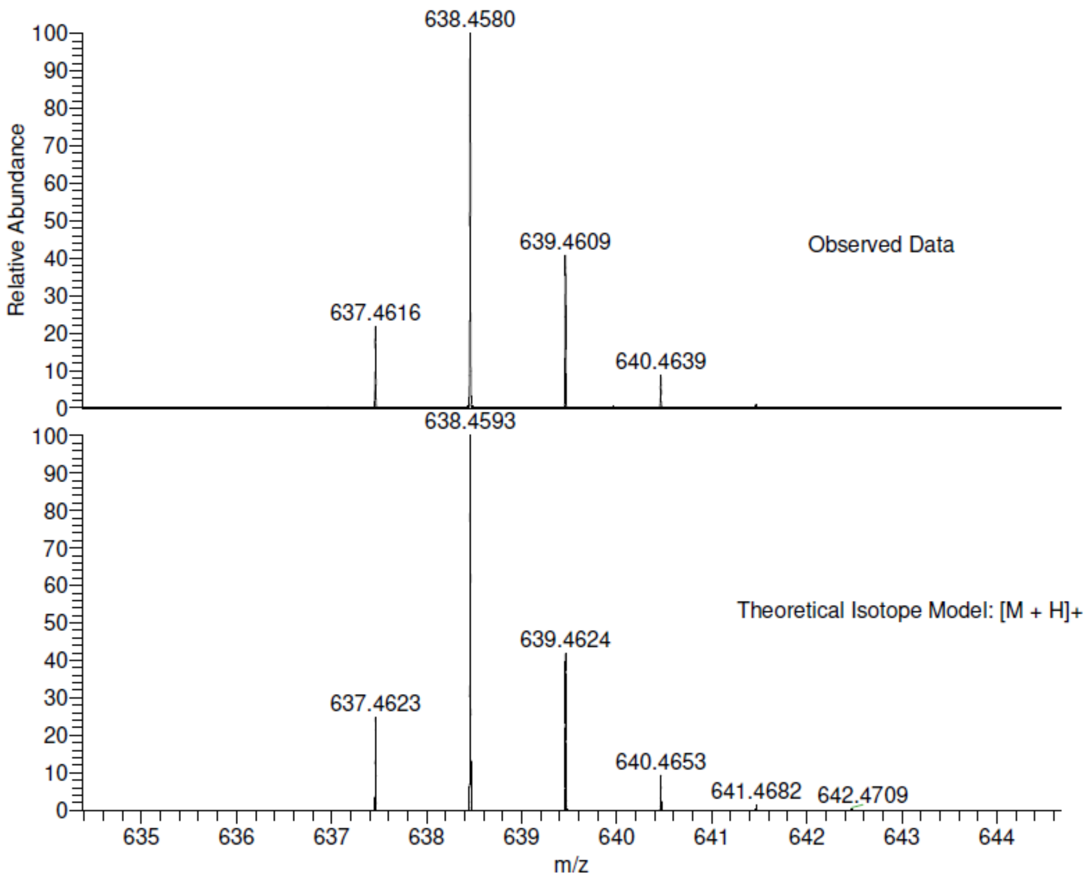
638.4580
639.4609
637.4616
640.4639
Observed Data
Relative Abundance
638.4593
639.4624
637.4623
640.4653
641.4682
642.4709
Theoretical Isotope Model: [M + H]+
m/z

Spectra for **14**

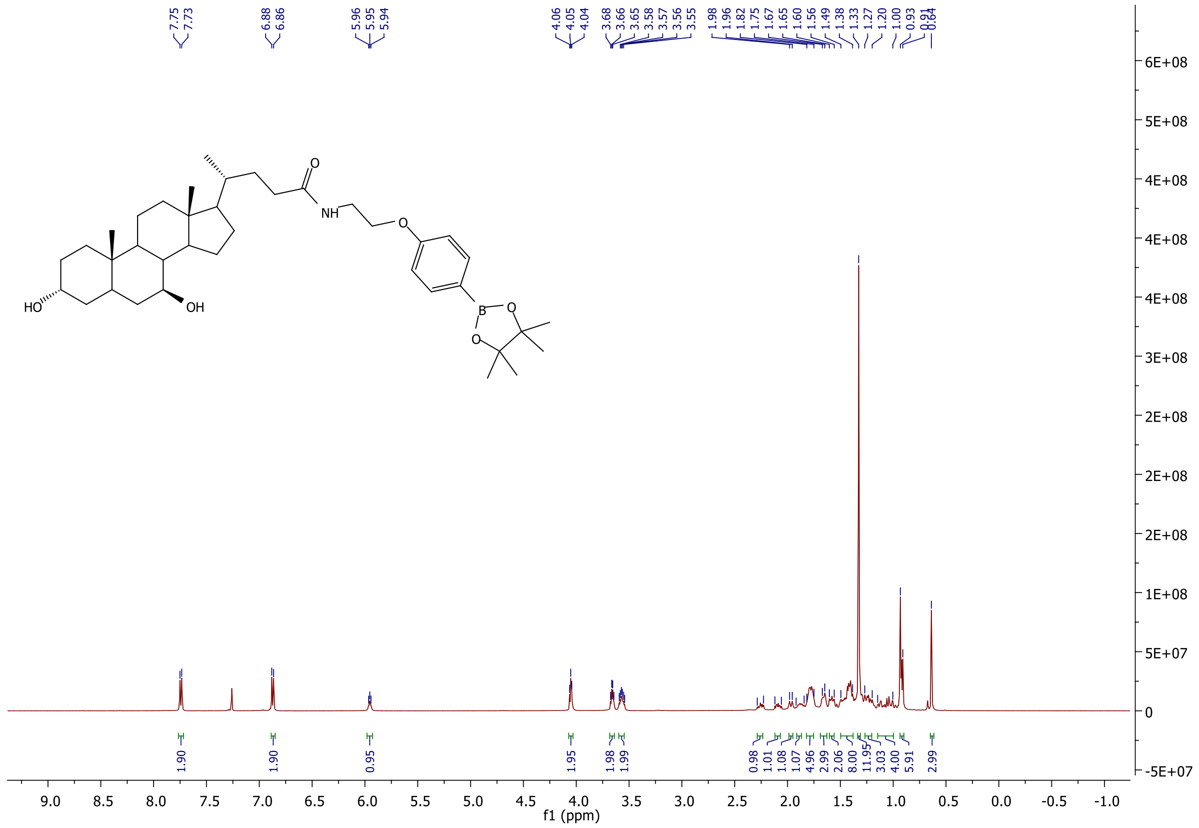

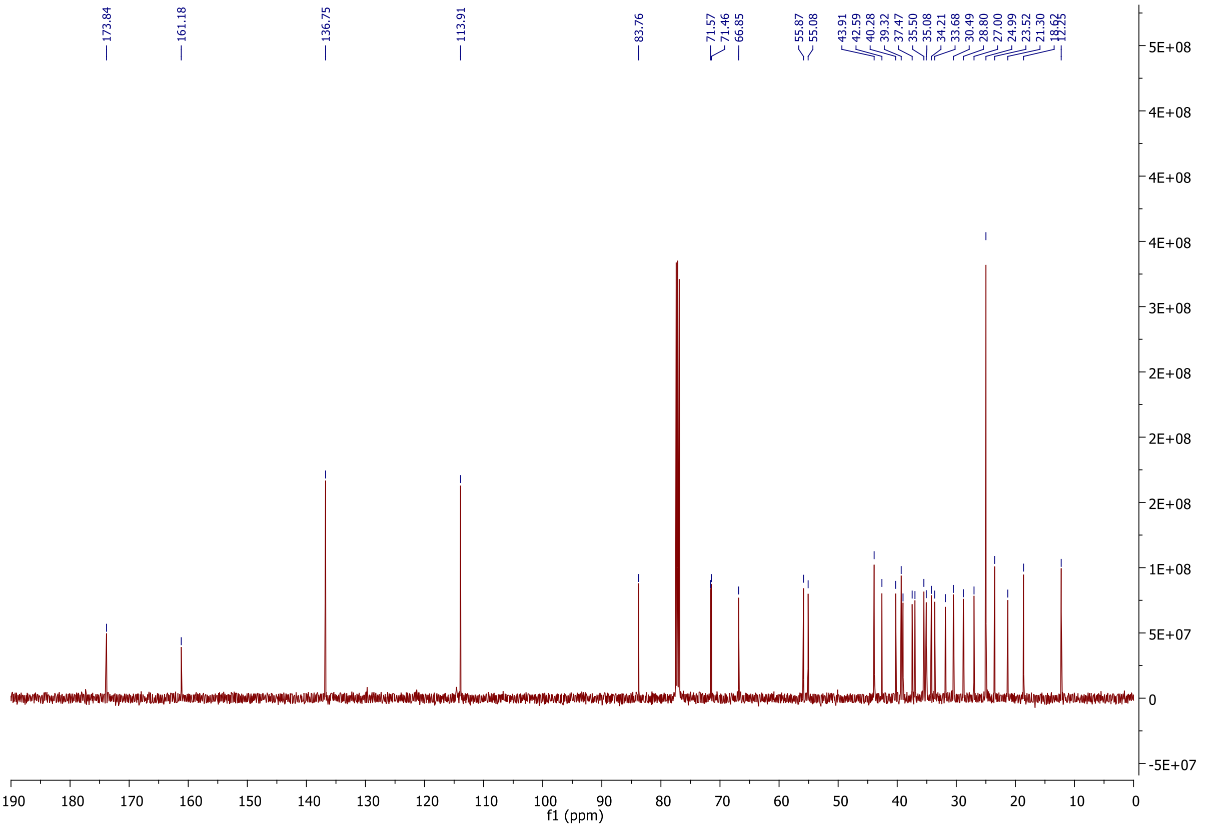

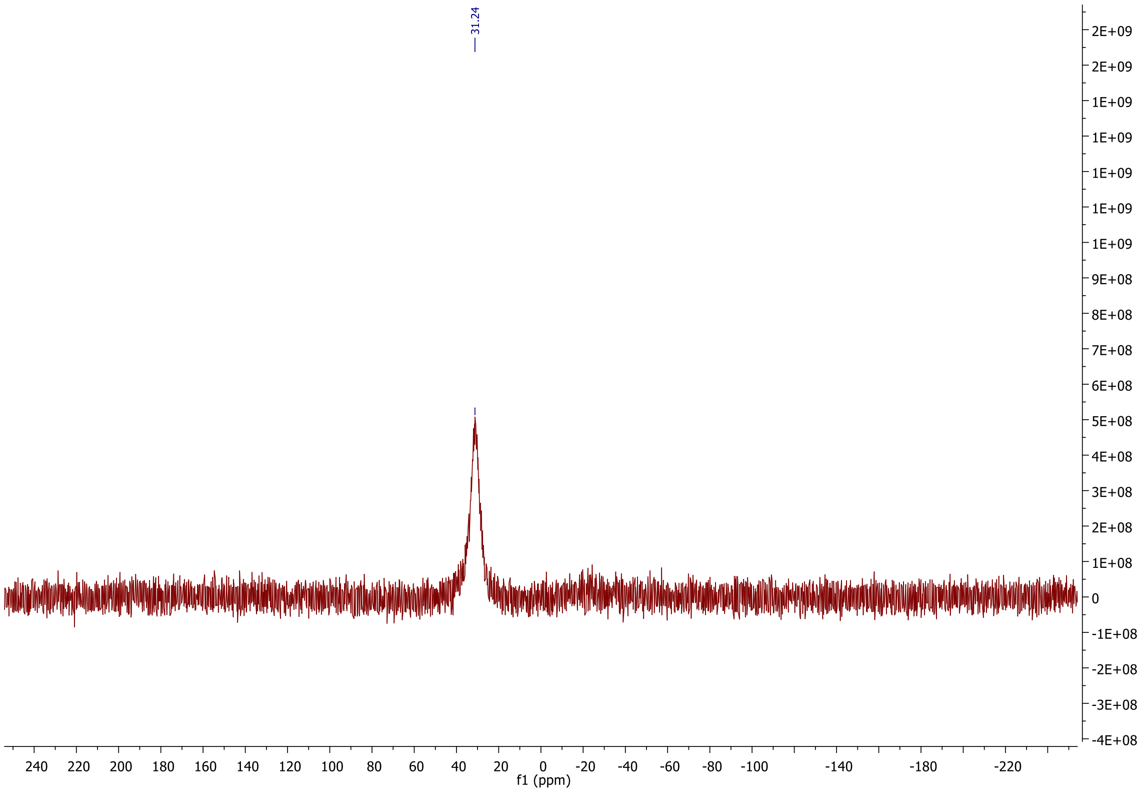

31.24
2E+09
2E+09
1E+09
1E+09
1E+09
1E+09
1E+09
9E+08
8E+08
7E+08
6E+08
5E+08
4E+08
3E+08
2E+08
1E+08
0
-1E+08
-2E+08
-3E+08
-4E+08
240 220 200 180 160 140 120 100 80 60 40 20 0 -20 -40 -60 -80 -100 -140 -180 -220
f1 (ppm)

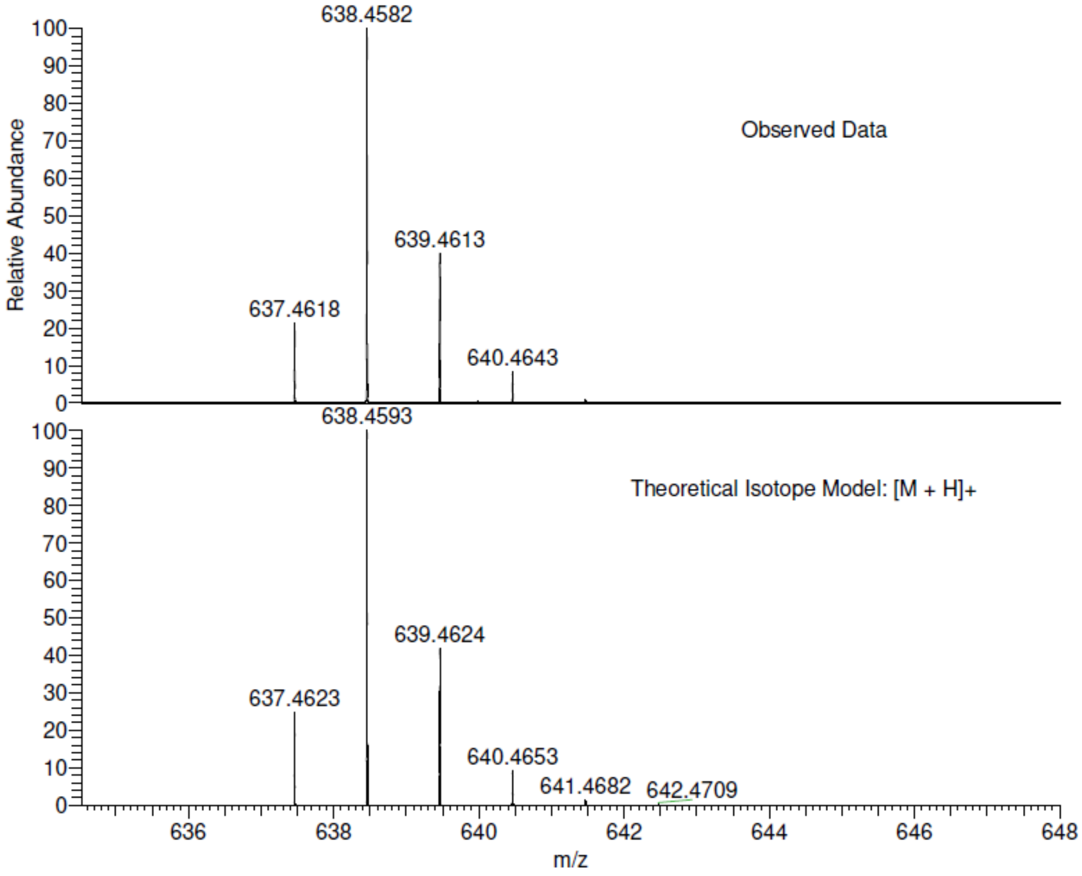

Relative Abundance
Observed Data
638.4582
639.4613
637.4618
640.4643
Theoretical Isotope Model: [M + H]+
638.4593
639.4624
637.4623
640.4653
641.4682
642.4709
636 638 640 642 644 646 648
m/z

Spectra for **15**

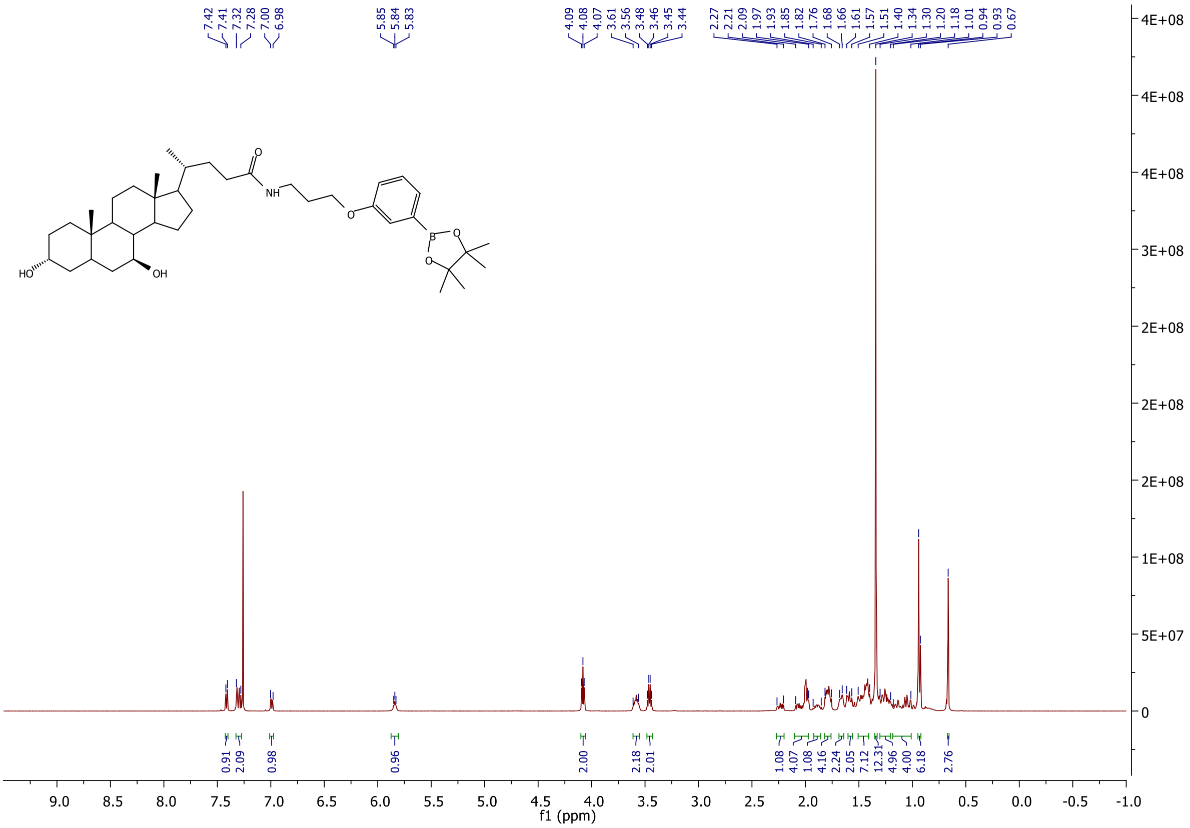

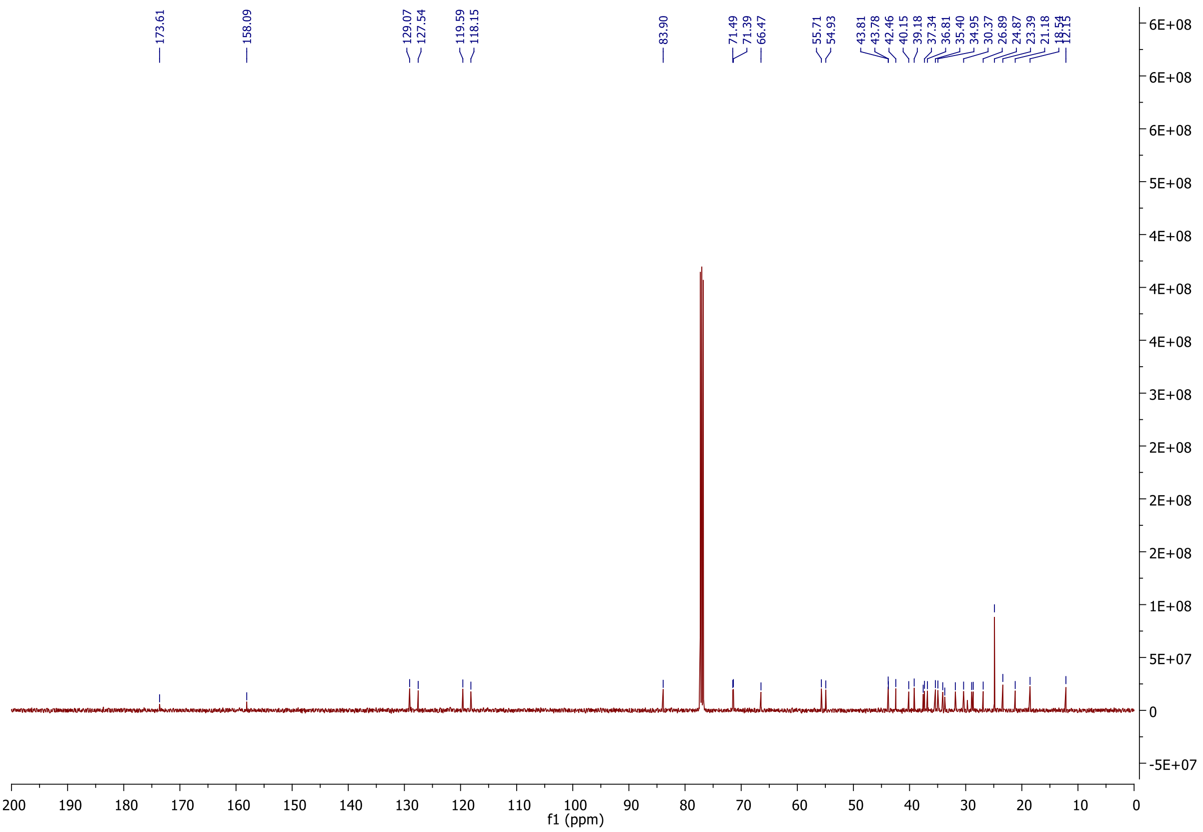
173.61
158.09
129.07
127.54
119.59
118.15
83.90
71.49
71.39
66.47
55.71
54.93
43.81
43.78
42.46
40.15
39.18
37.34
36.81
35.40
34.95
30.37
26.89
24.87
23.39
21.18
18.54
12.15
f1 (ppm)

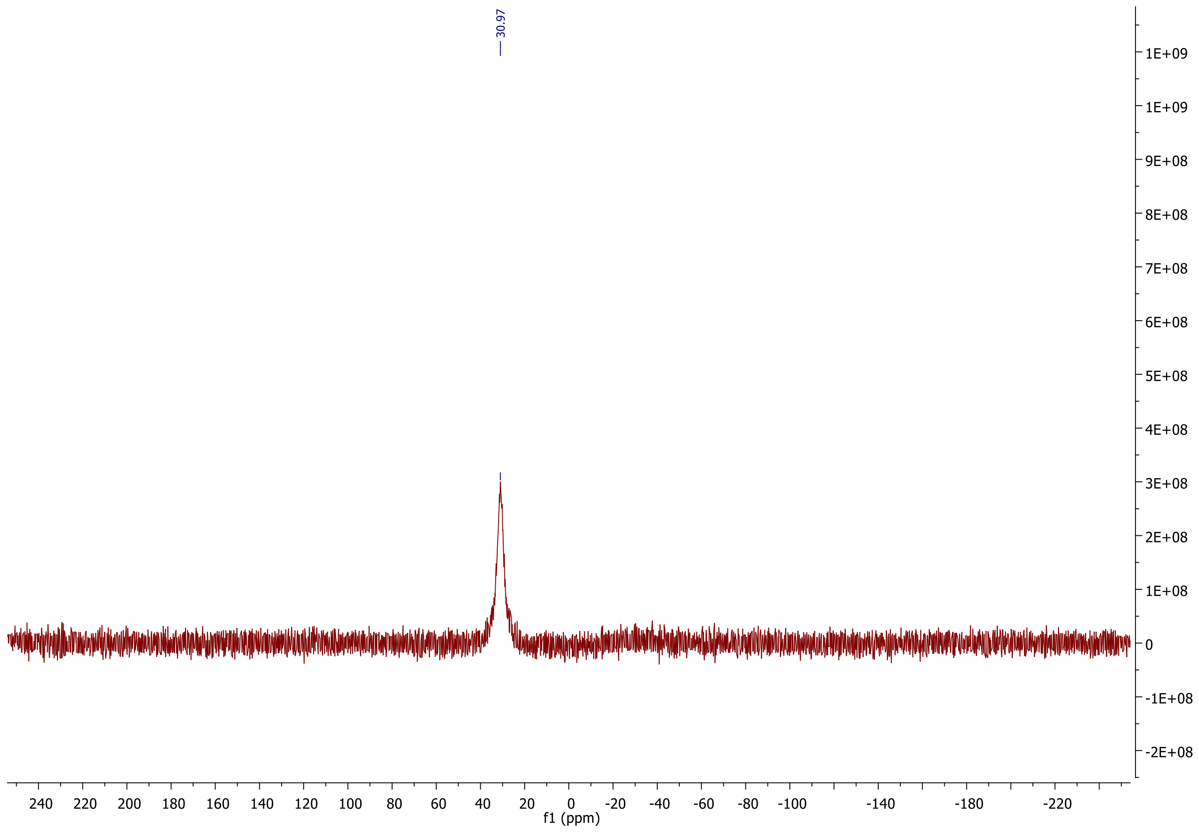
30.97
f1 (ppm)

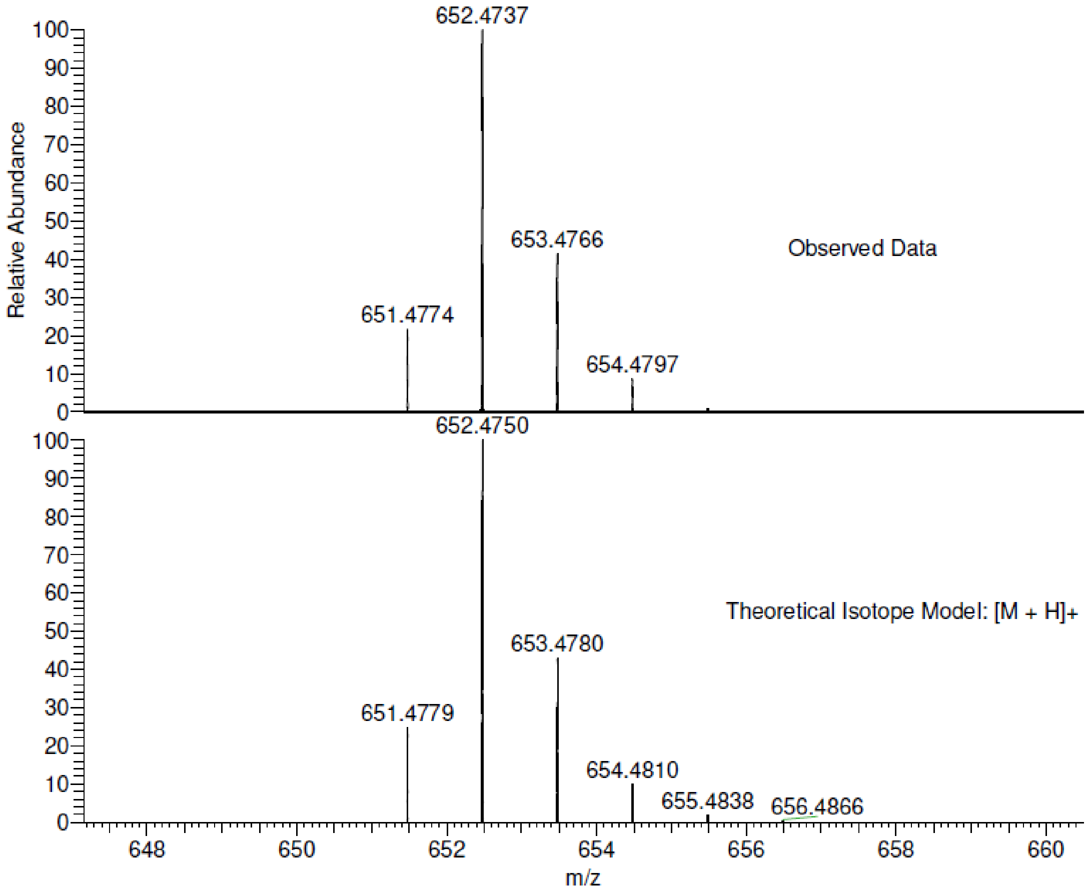

Spectra for **16**

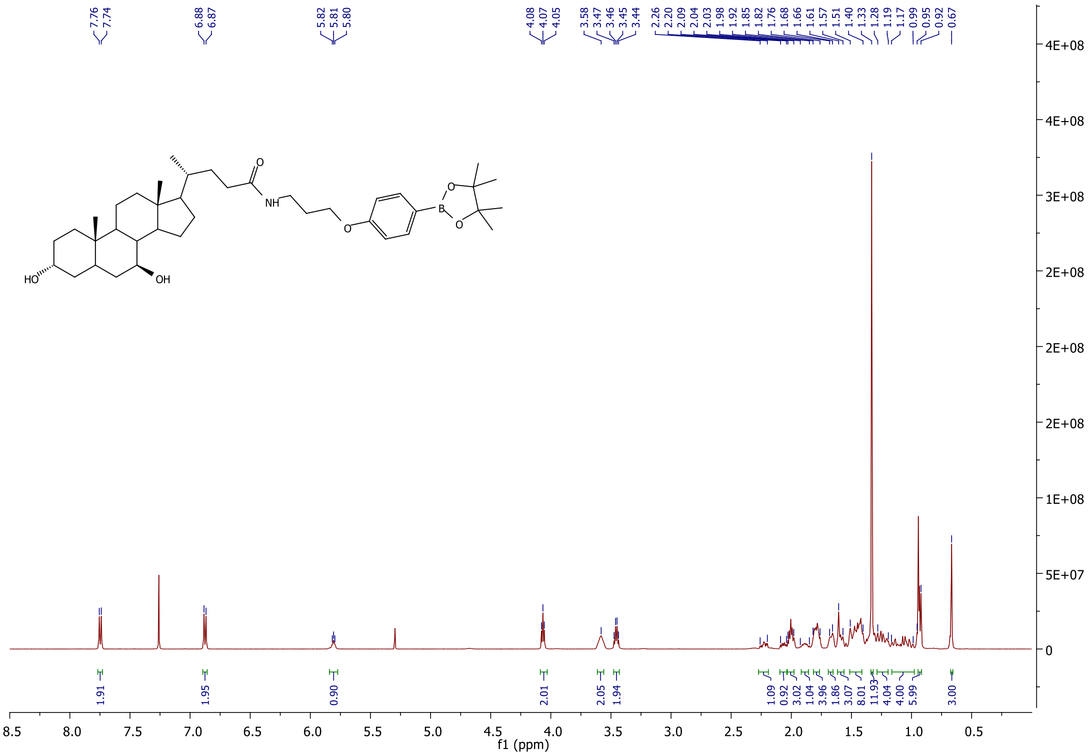

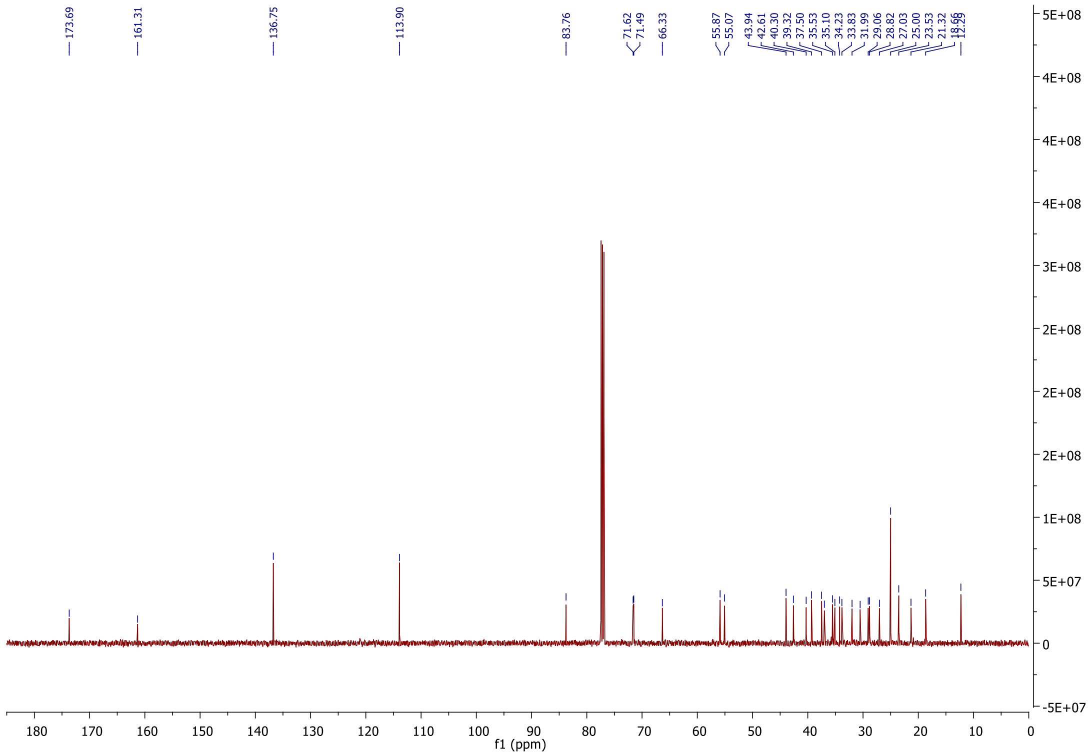
173.69
161.31
136.75
113.90
83.76
71.62
71.49
66.33
55.87
55.07
43.94
42.61
40.30
39.32
37.50
35.53
35.10
34.23
33.83
31.99
29.06
28.82
27.03
25.00
23.53
21.32
f1 (ppm)

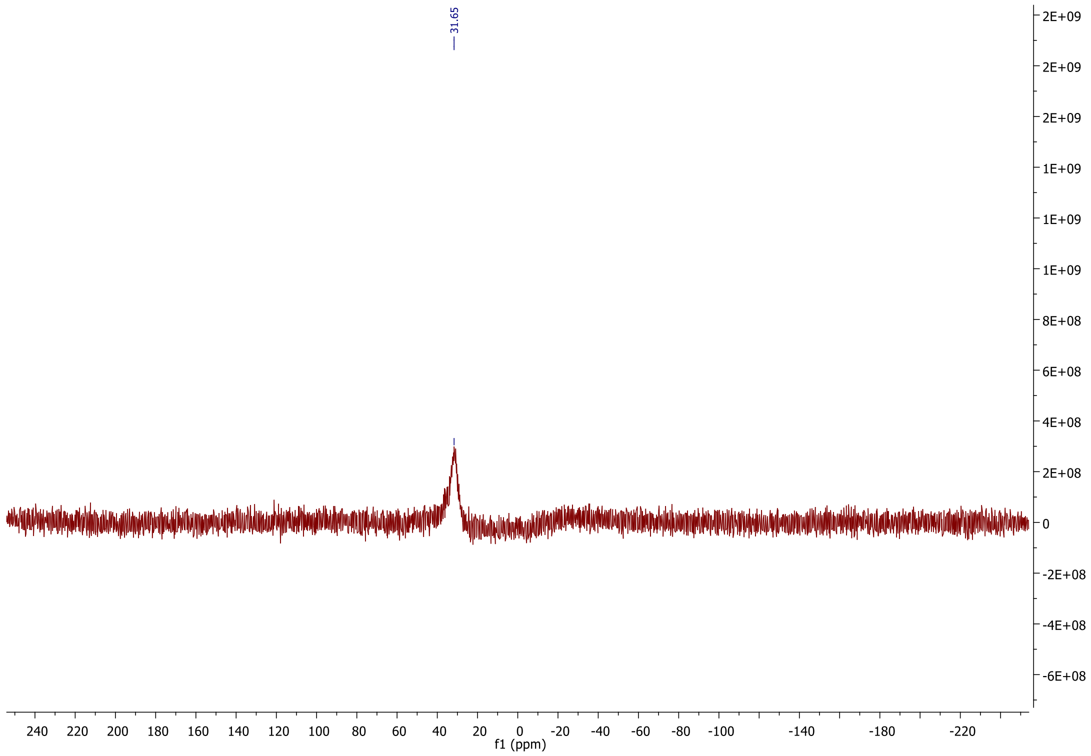
31.65
f1 (ppm)

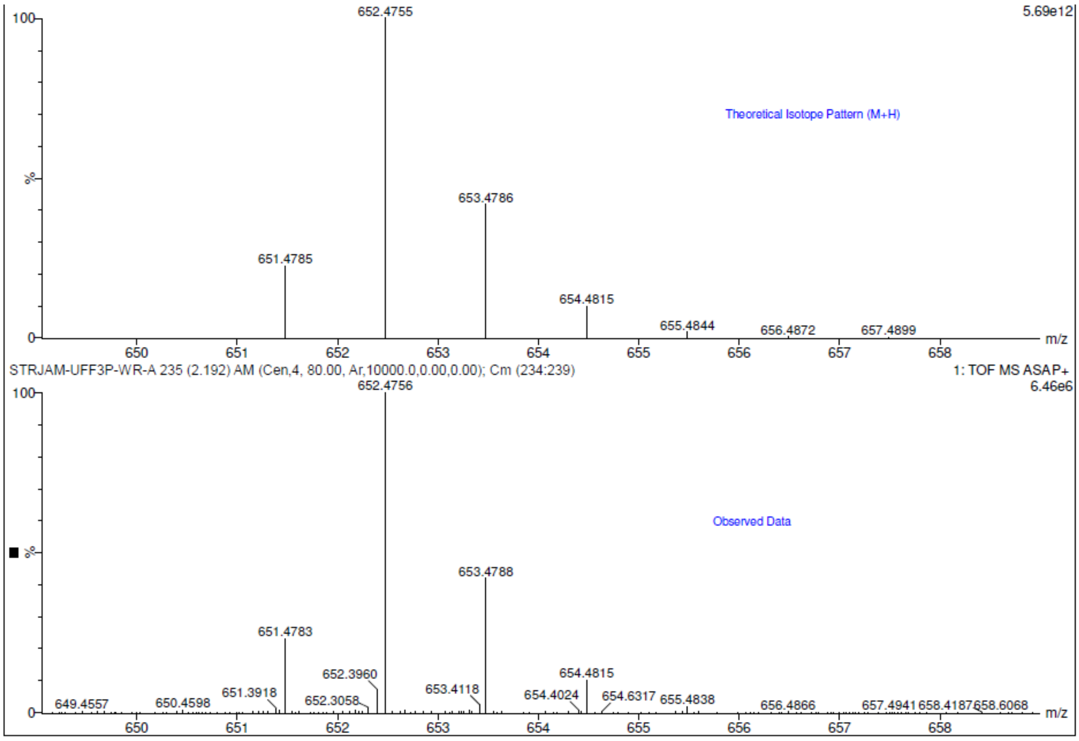

Spectra for **17**

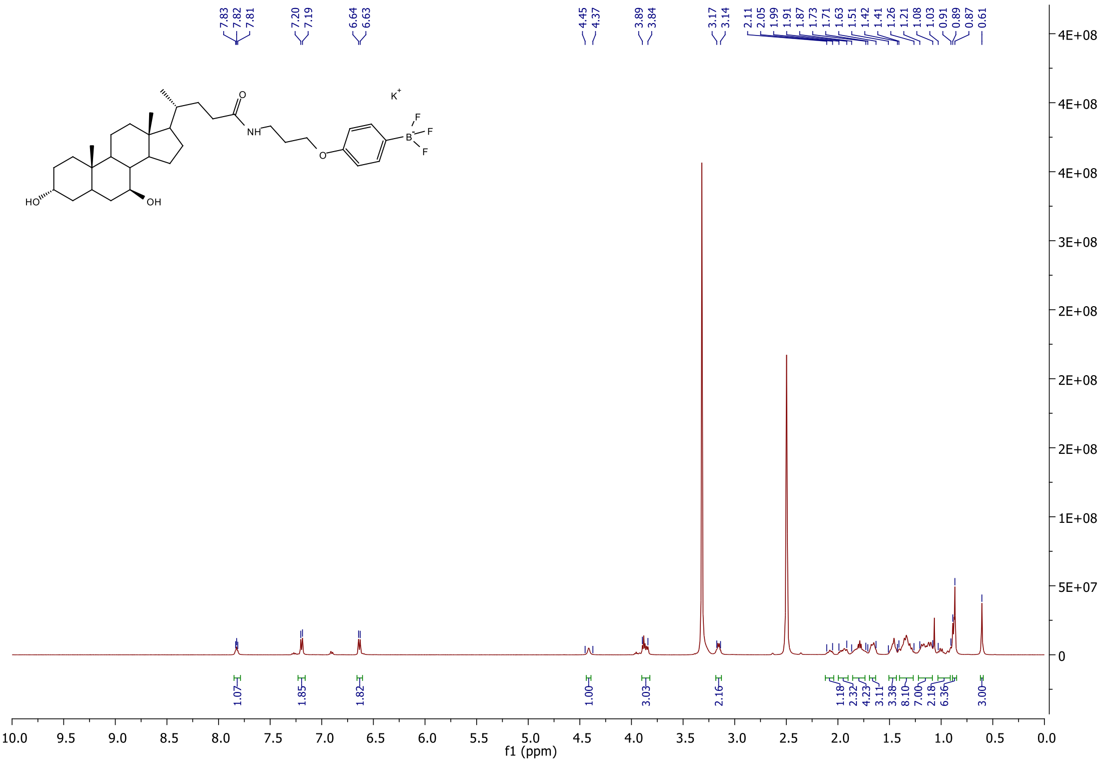

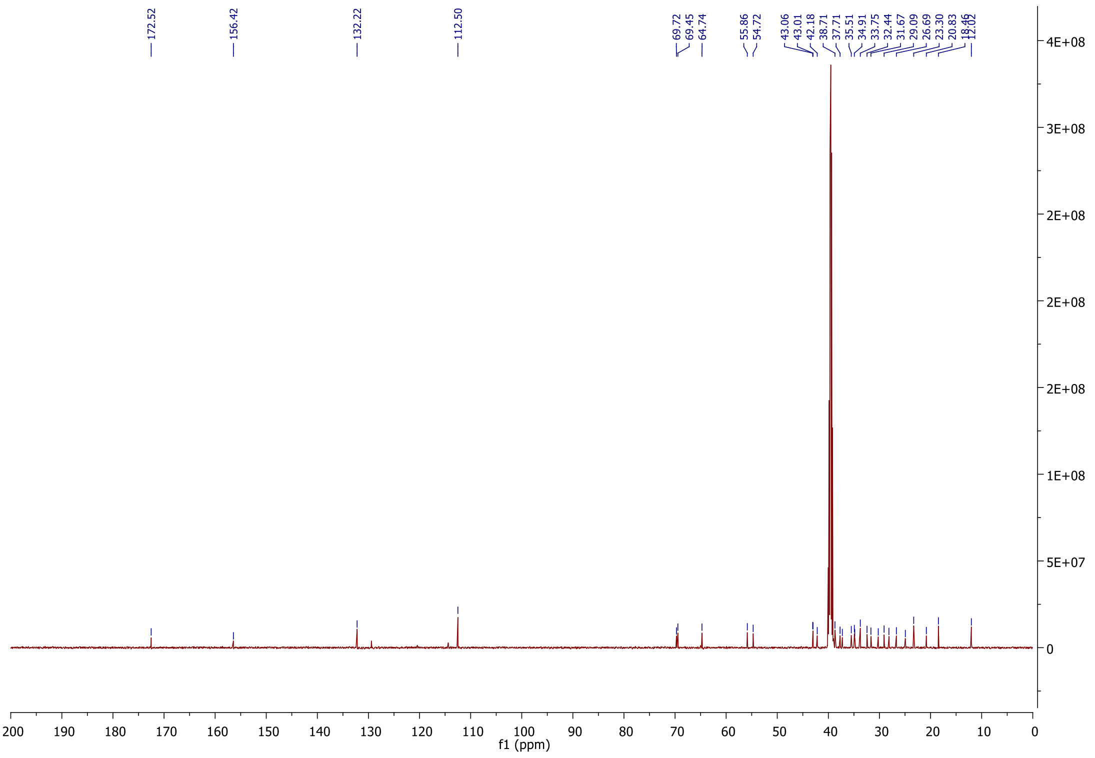
172.52
156.42
132.22
112.50
69.72
69.45
64.74
55.86
54.72
43.06
43.01
42.18
38.71
37.71
35.51
34.91
33.75
32.44
31.67
29.09
26.69
23.30
20.83
18.46
12.02
4E+08
3E+08
2E+08
2E+08
2E+08
1E+08
5E+07
0
200 190 180 170 160 150 140 130 120 110 100 90 80 70 60 50 40 30 20 10 0
f1 (ppm)

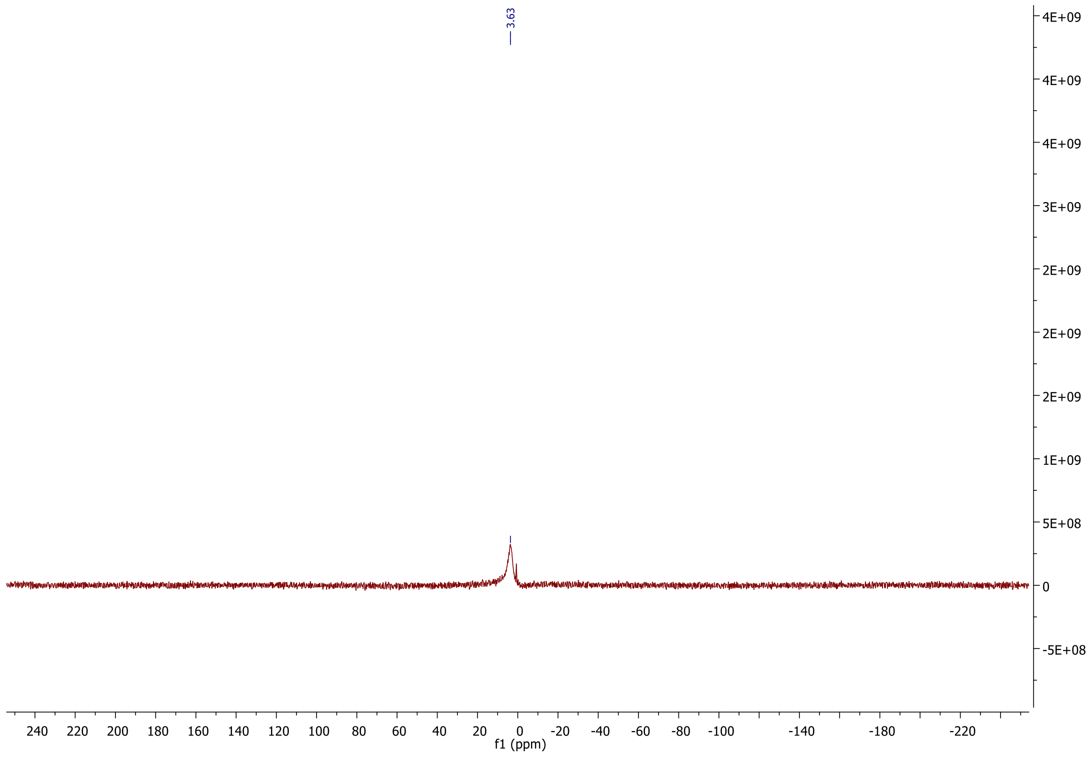
3.63
4E+09
4E+09
4E+09
3E+09
2E+09
2E+09
2E+09
1E+09
5E+08
0
-5E+08
240 220 200 180 160 140 120 100 80 60 40 20 0 -20 -40 -60 -80 -100 -140 -180 -220
f1 (ppm)

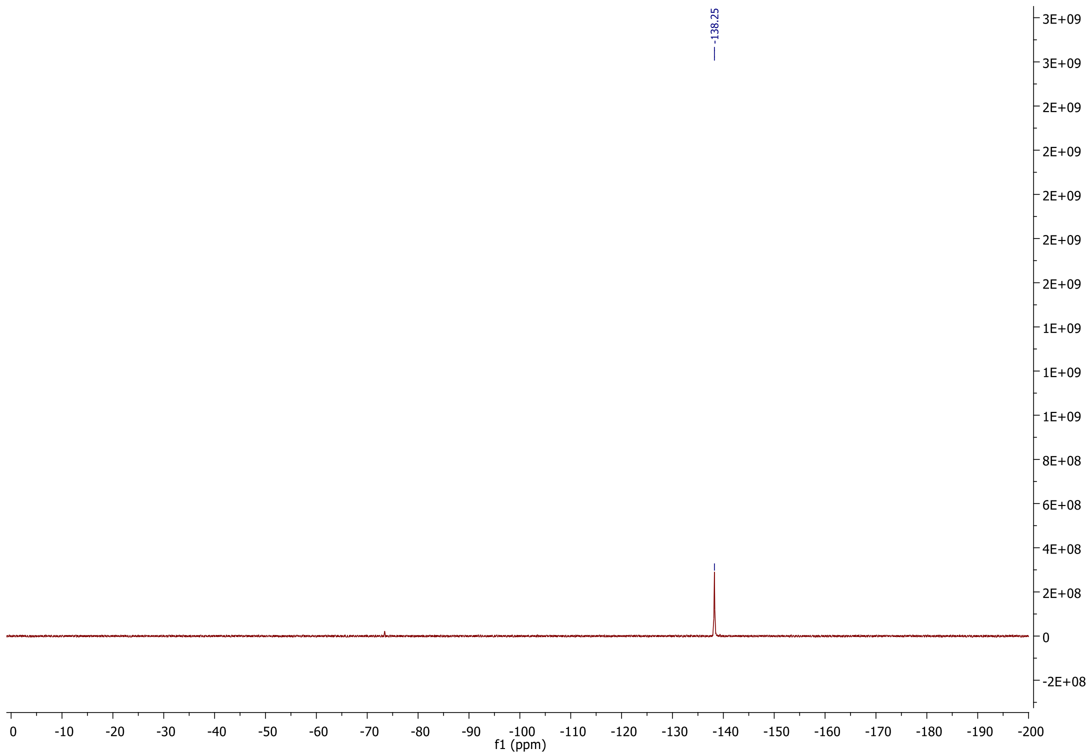

-138.25
3E+09
3E+09
2E+09
2E+09
2E+09
2E+09
2E+09
1E+09
1E+09
1E+09
8E+08
6E+08
4E+08
2E+08
0
-2E+08
0
-10
-20
-30
-40
-50
-60
-70
-80
-90
-100
-110
-120
-130
-140
-150
-160
-170
-180
-190
-200
f1 (ppm)

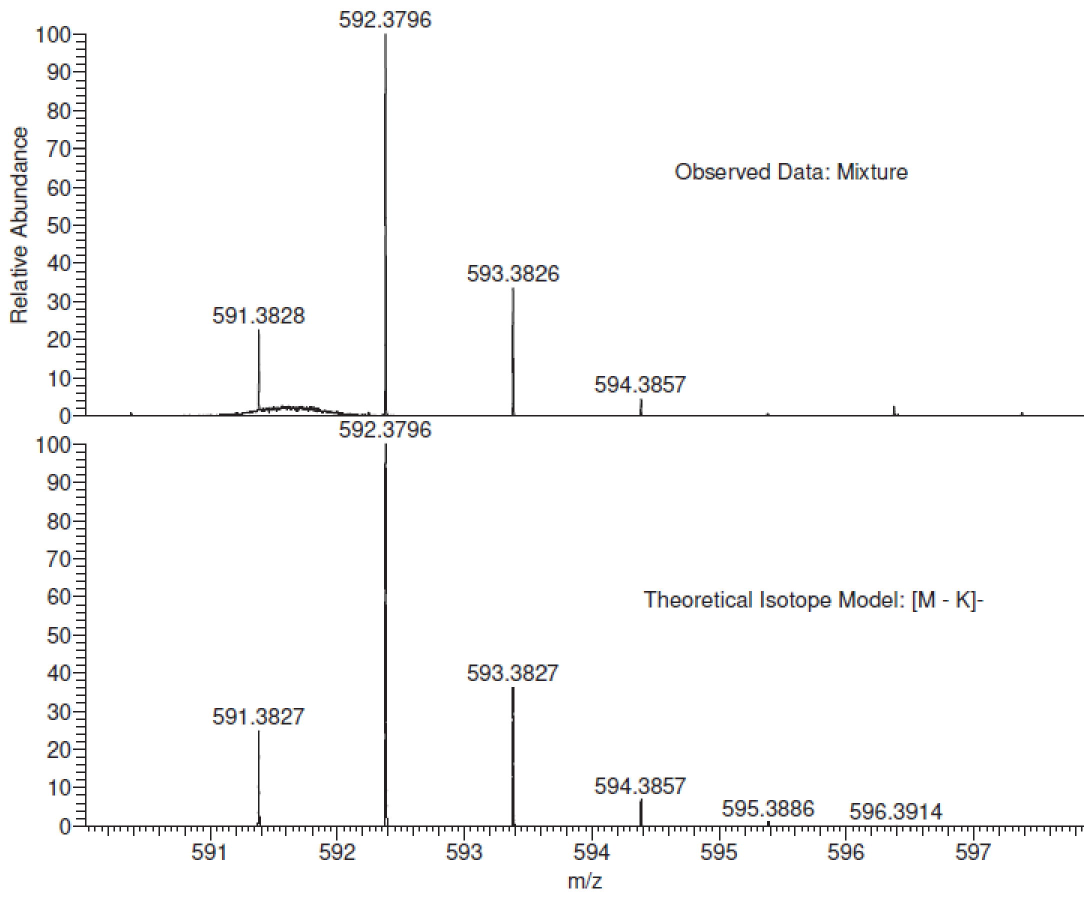

Relative Abundance
592.3796
Observed Data: Mixture
593.3826
591.3828
594.3857
592.3796
Theoretical Isotope Model: [M - K]-
593.3827
591.3827
594.3857
595.3886
596.3914
591
592
593
594
595
596
597
m/z

Spectra for **18**

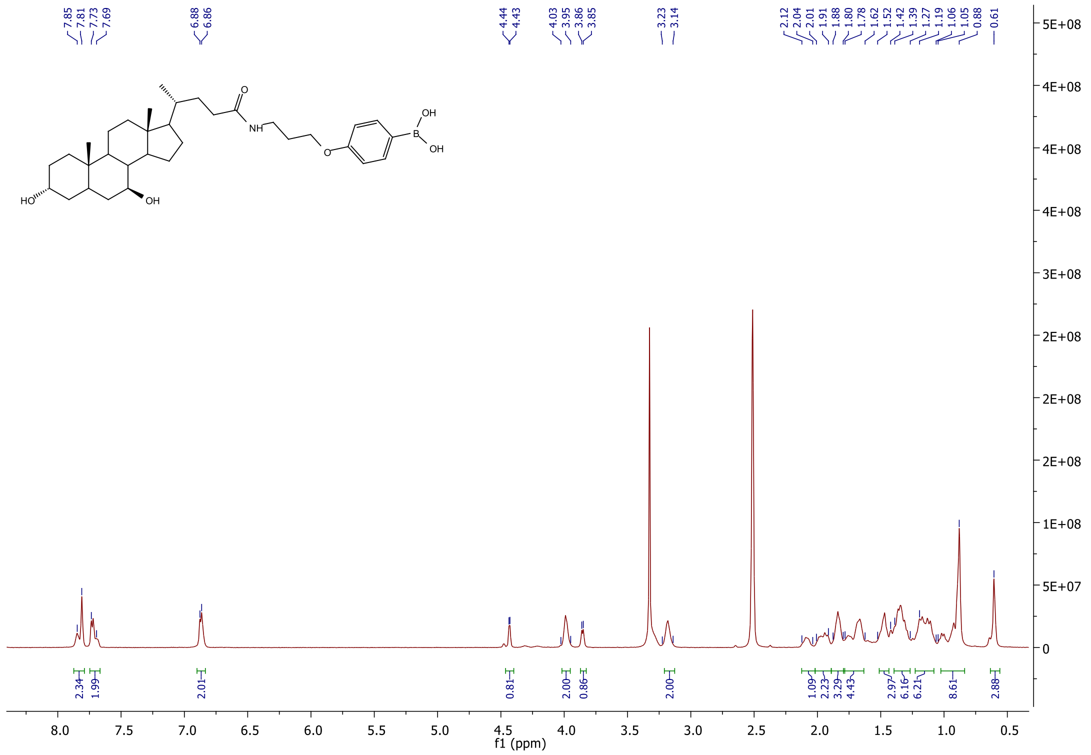

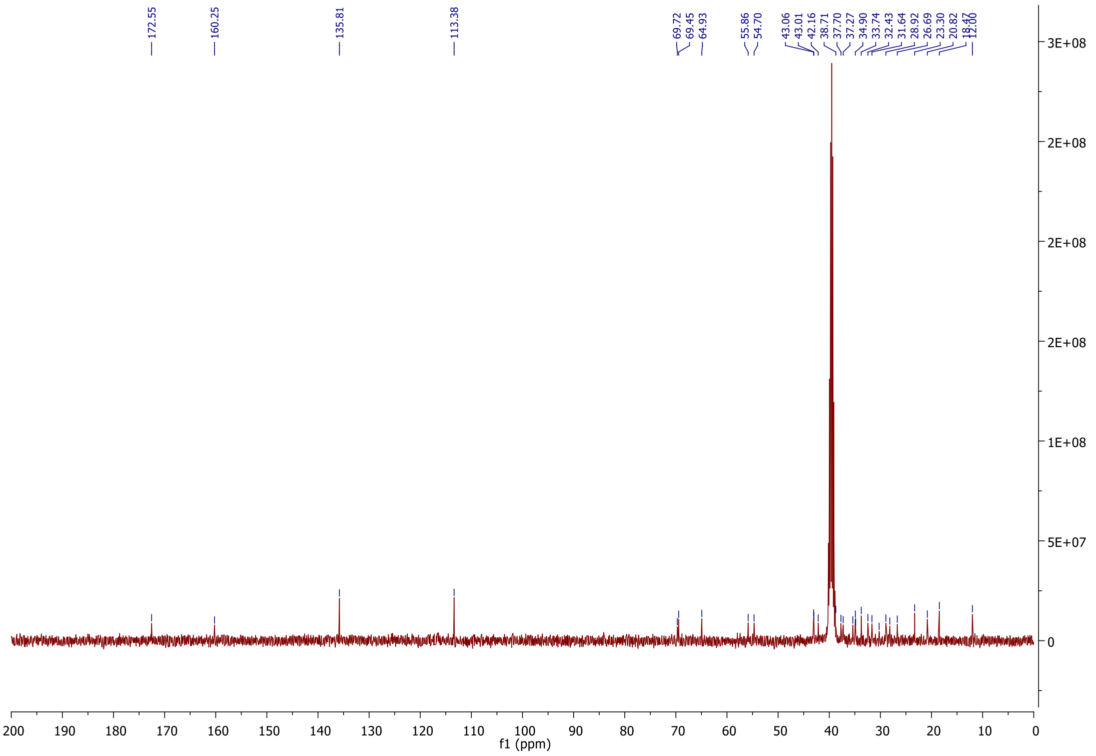

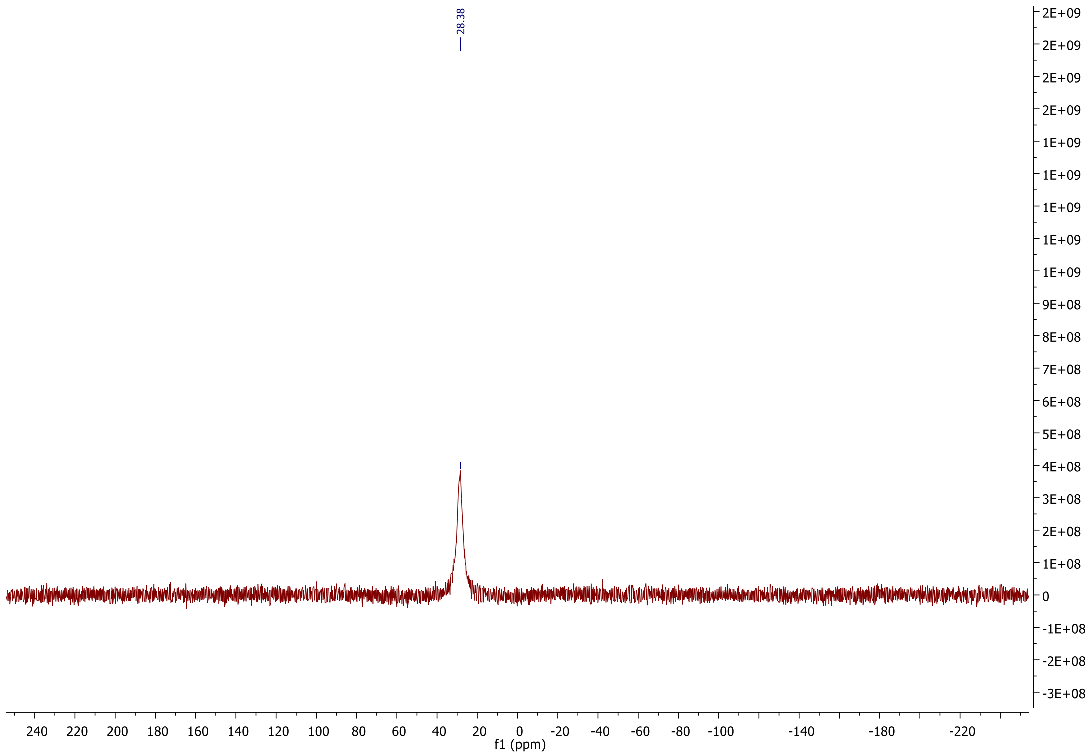

28.38
2E+09
1E+09
9E+08
8E+08
7E+08
6E+08
5E+08
4E+08
3E+08
2E+08
1E+08
0
-1E+08
-2E+08
-3E+08
240 220 200 180 160 140 120 100 80 60 40 20 0 -20 -40 -60 -80 -100 -140 -180 -220
f1 (ppm)

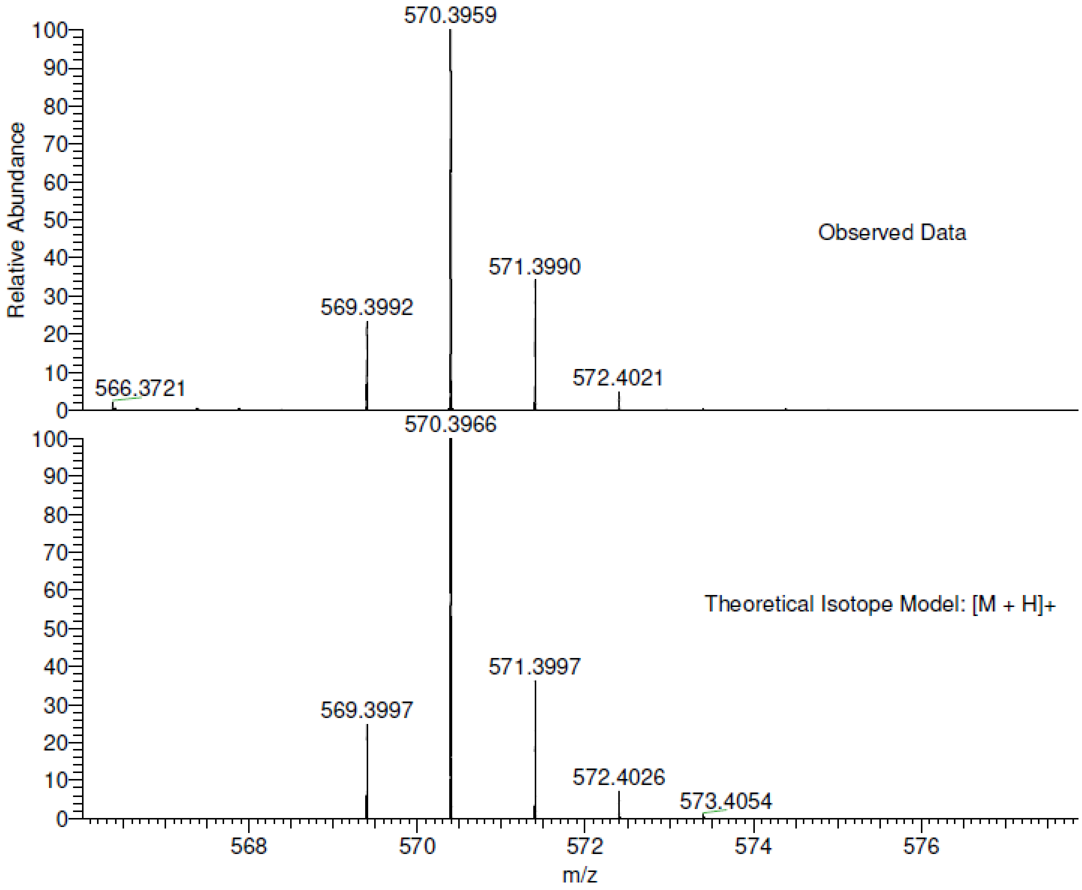

Relative Abundance
Observed Data
570.3959
571.3990
569.3992
572.4021
566.3721
Theoretical Isotope Model: [M + H]+
570.3966
571.3997
569.3997
572.4026
573.4054
568
570
572
574
576
m/z

Spectra for **19**

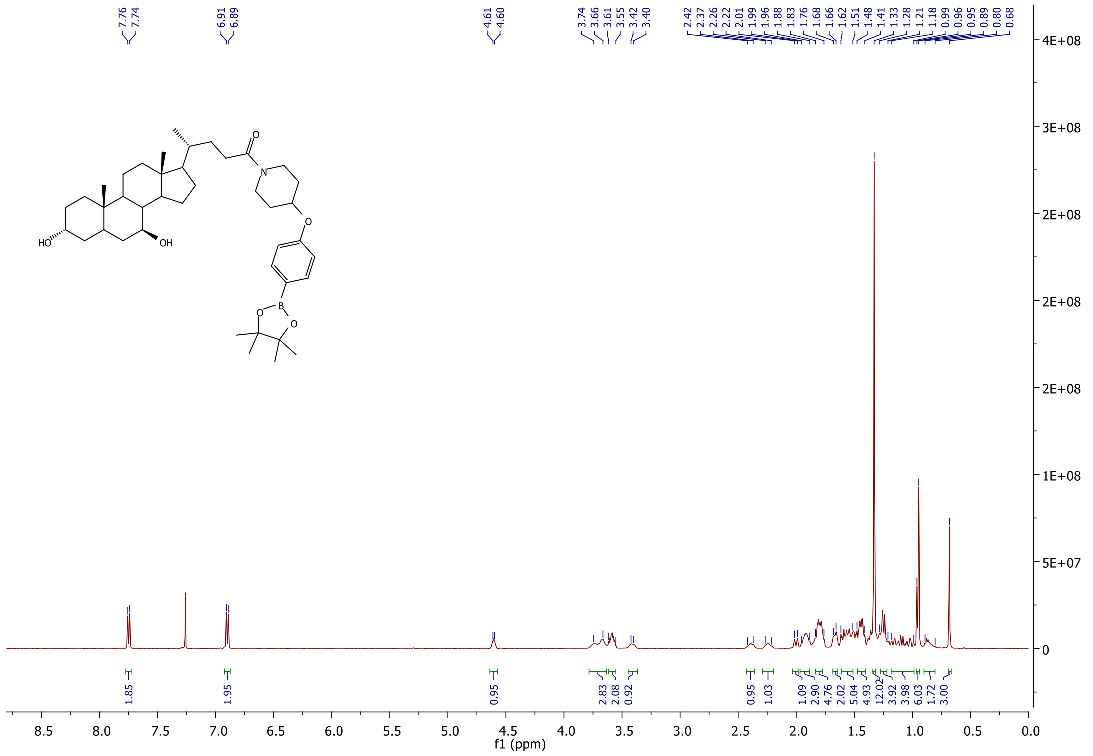

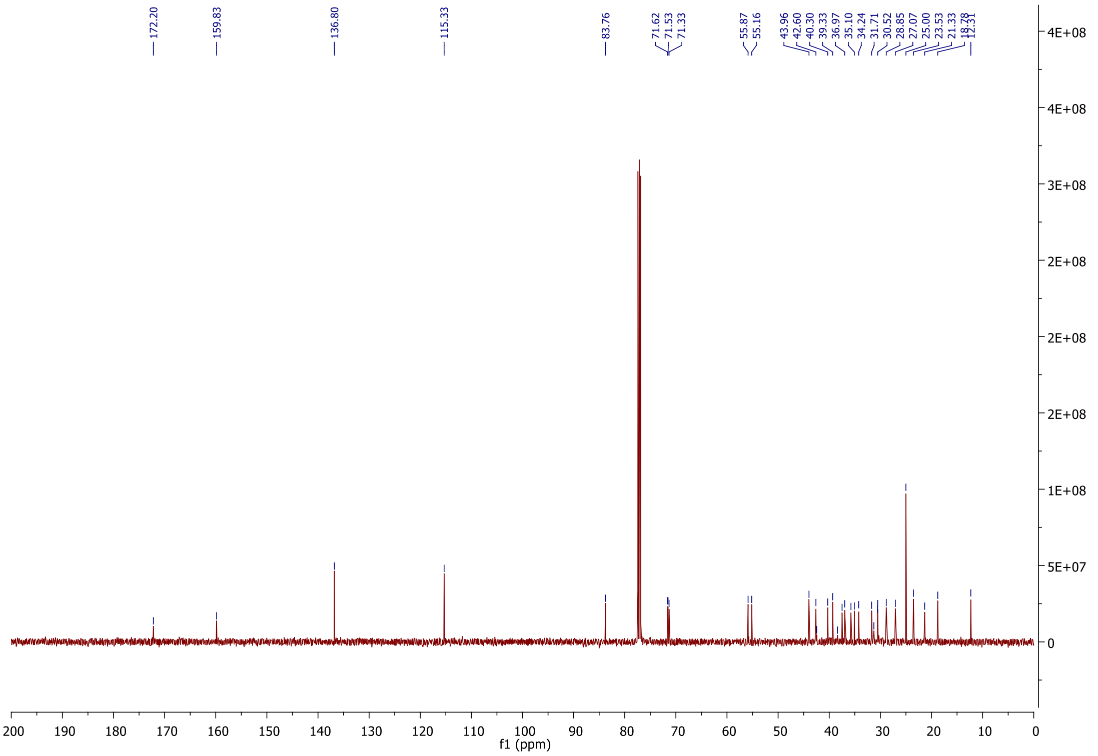

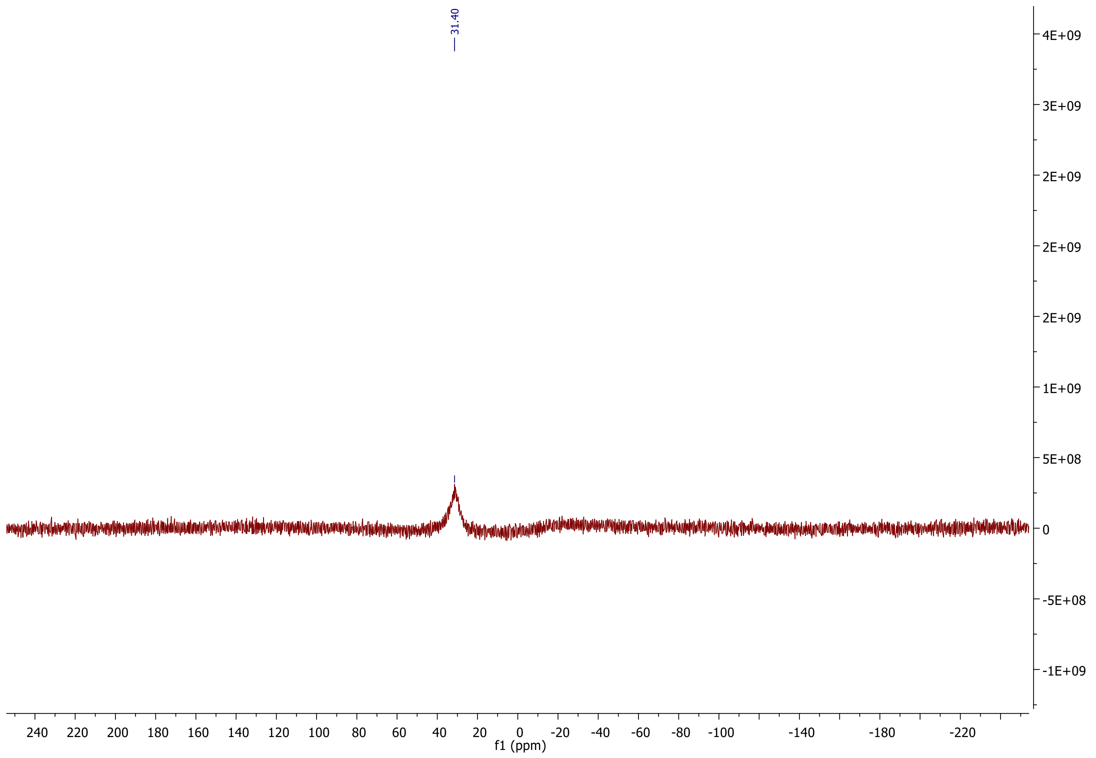
31.40
4E+09
3E+09
2E+09
2E+09
2E+09
1E+09
5E+08
0
-5E+08
-1E+09
240 220 200 180 160 140 120 100 80 60 40 20 0 -20 -40 -60 -80 -100 -140 -180 -220
f1 (ppm)

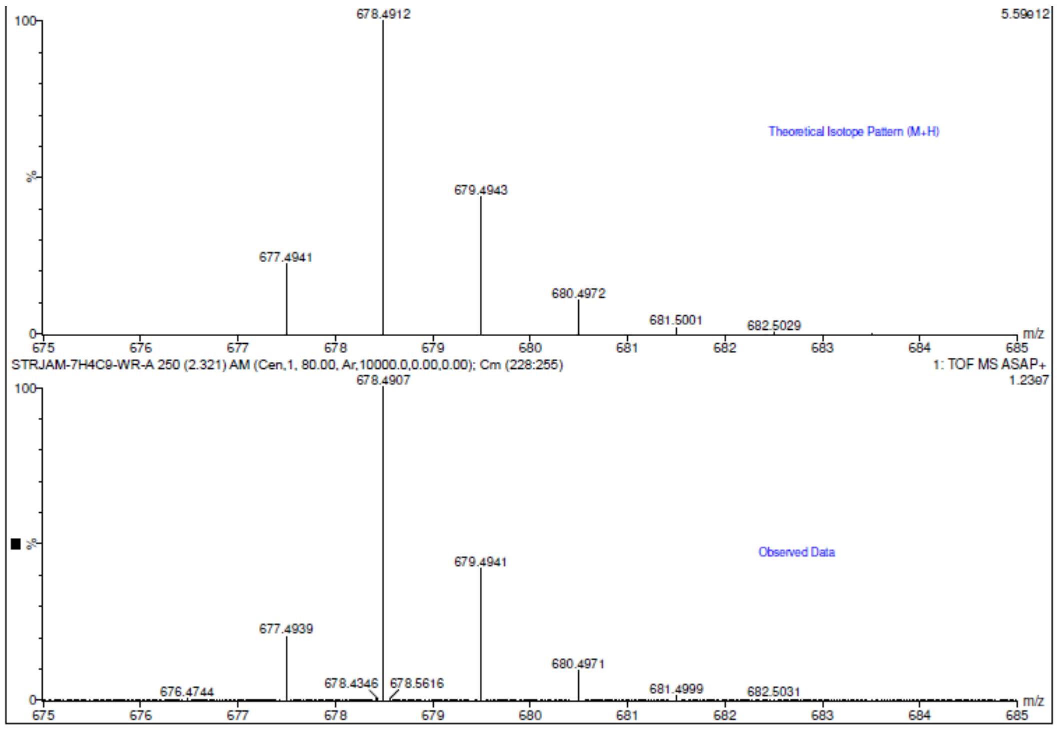
678.4912
5.59e12
100
%
0
Theoretical Isotope Pattern (M+H)
679.4943
677.4941
680.4972
681.5001
682.5029
m/z
675 676 677 678 679 680 681 682 683 684 685
STRJAM-7H4C9-WR-A 250 (2.321) AM (Cen,1, 80.00, Ar,10000.0,0.00,0.00); Cm (228:255)
1: TOF MS ASAP+
1.23e7
678.4907
Observed Data
679.4941
677.4939
680.4971
676.4744
678.4346
678.5616
681.4999
682.5031

Spectra for **20**

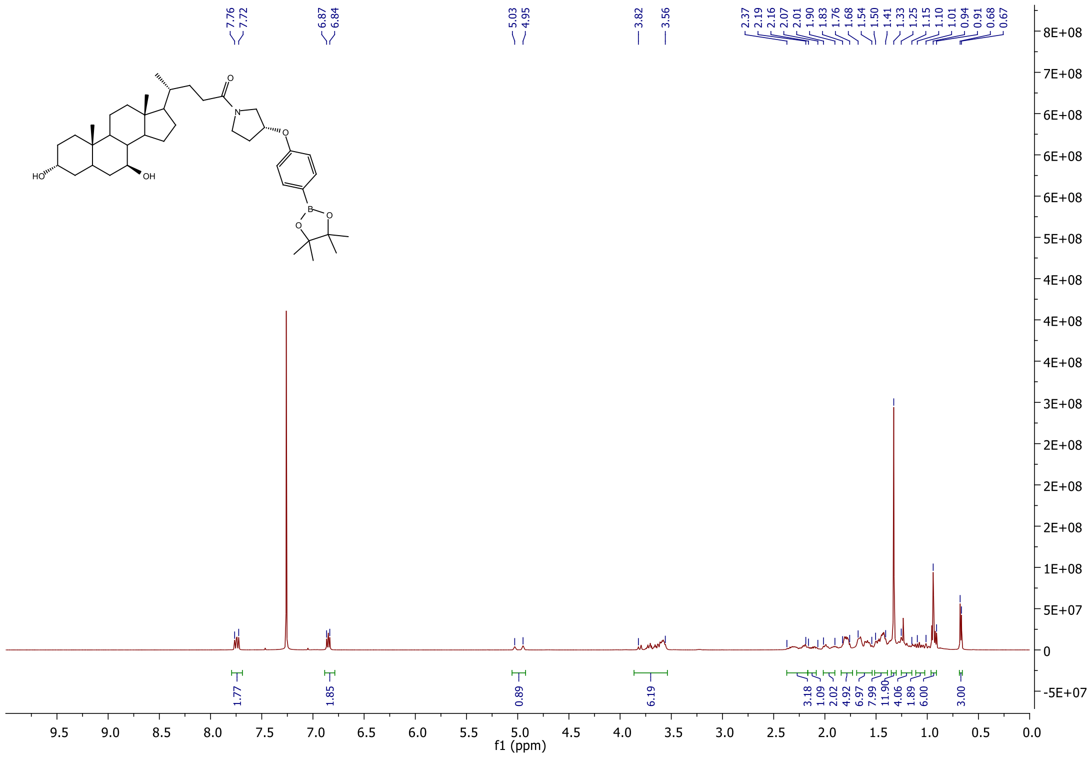

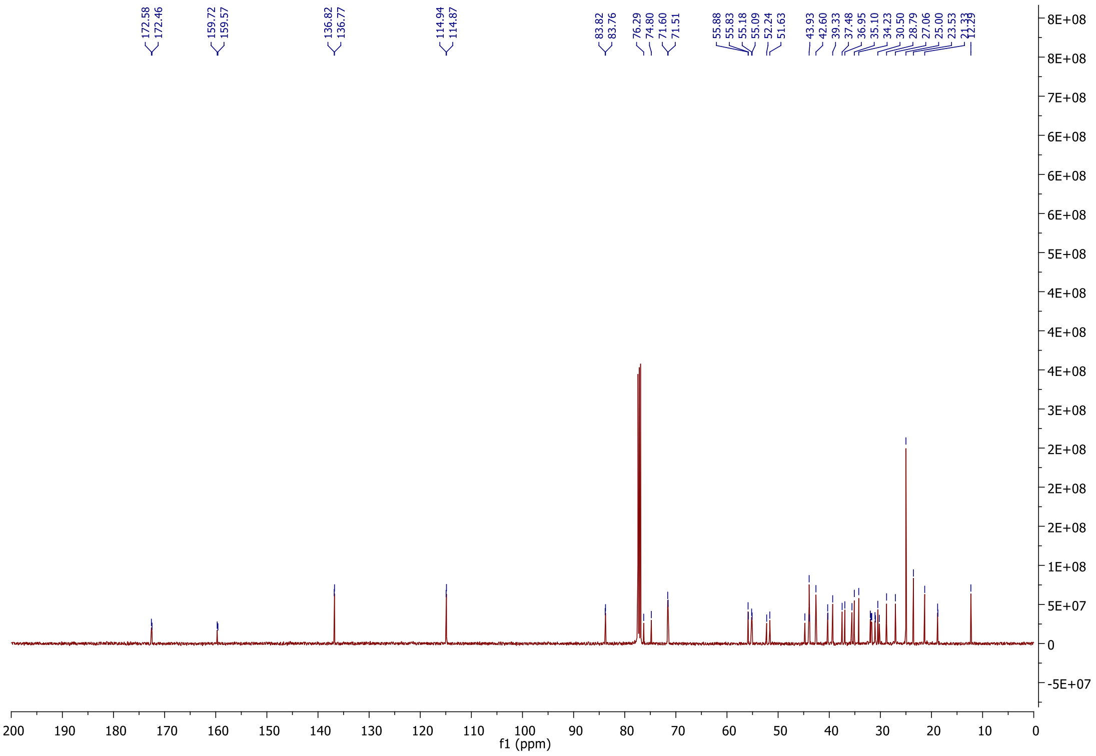

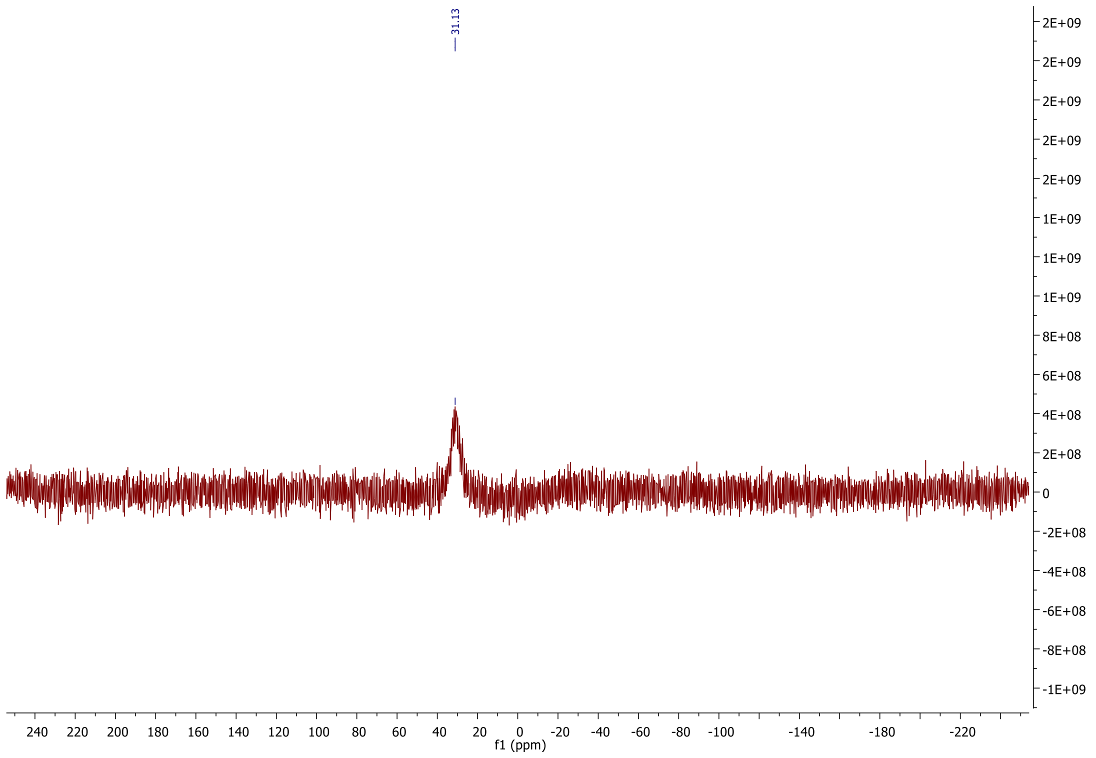
31.13
2E+09
2E+09
2E+09
2E+09
2E+09
1E+09
1E+09
1E+09
8E+08
6E+08
4E+08
2E+08
0
-2E+08
-4E+08
-6E+08
-8E+08
-1E+09
240 220 200 180 160 140 120 100 80 60 40 20 0 -20 -40 -60 -80 -100 -140 -180 -220
f1 (ppm)

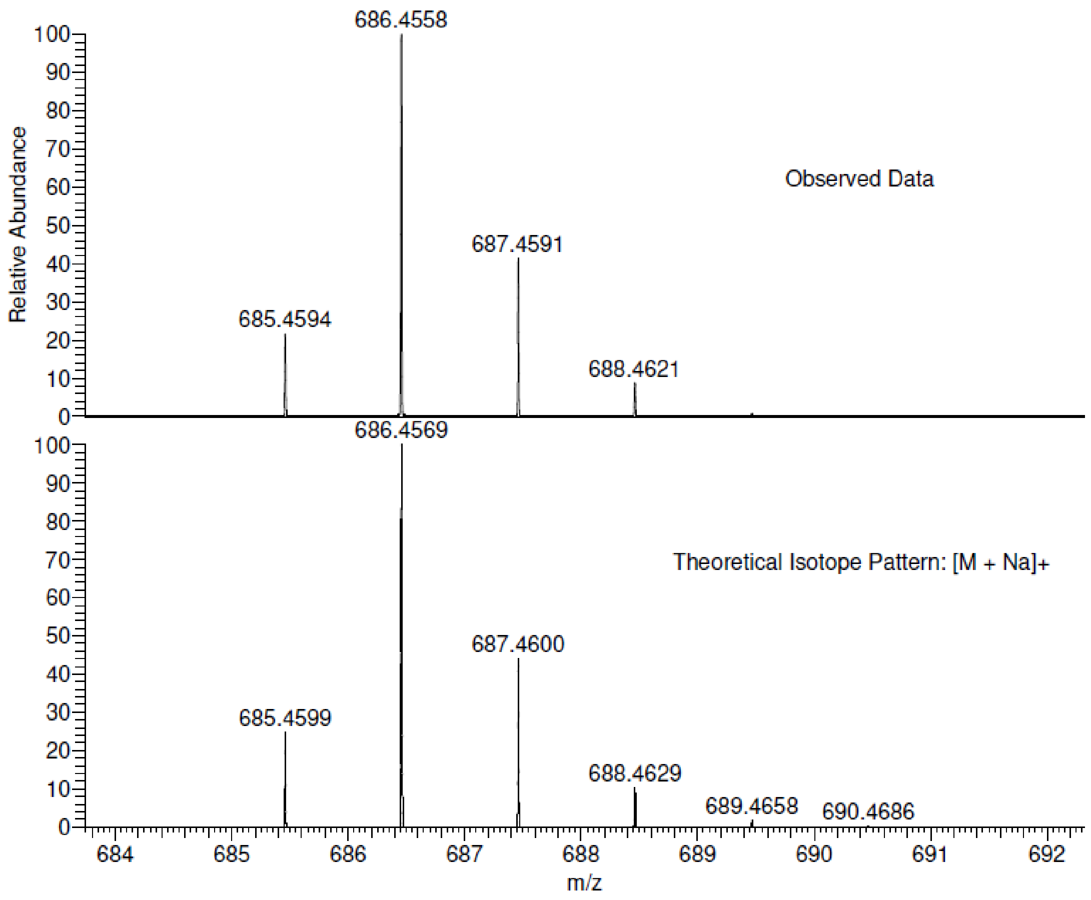
686.4558
Observed Data
687.4591
685.4594
688.4621
686.4569
Theoretical Isotope Pattern: [M + Na]+
687.4600
685.4599
688.4629
689.4658
690.4686
Relative Abundance
m/z
684 685 686 687 688 689 690 691 692

Spectra for **21**

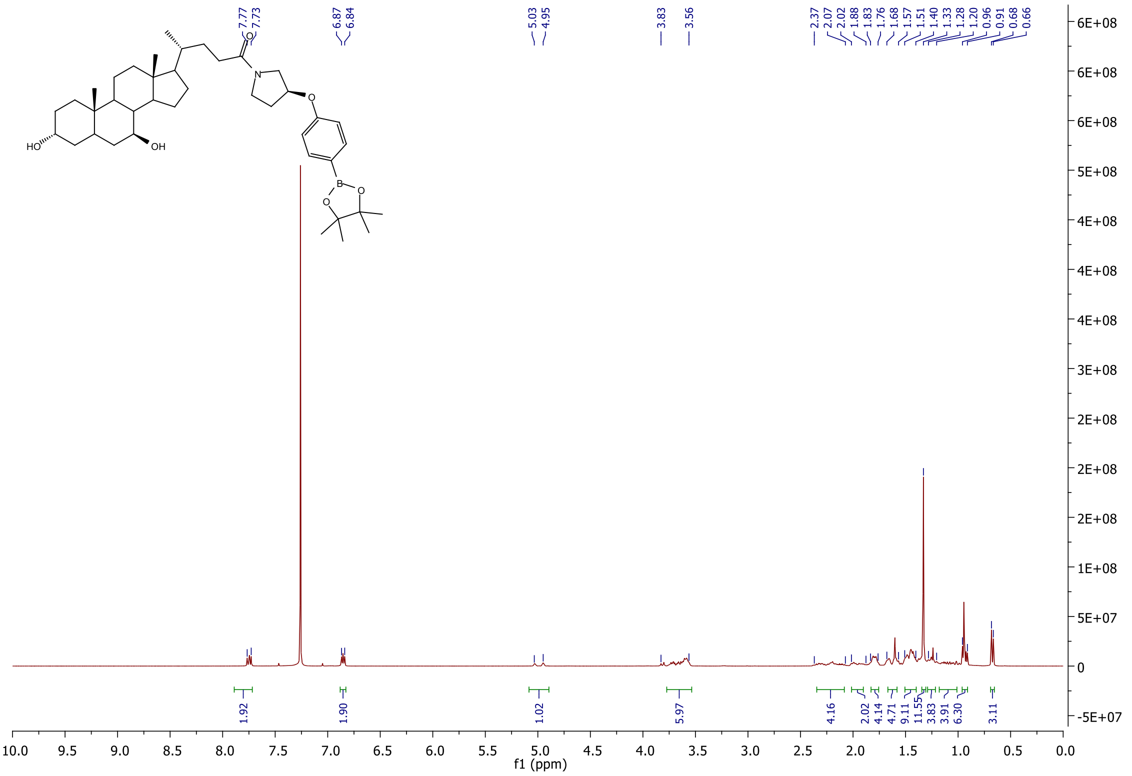

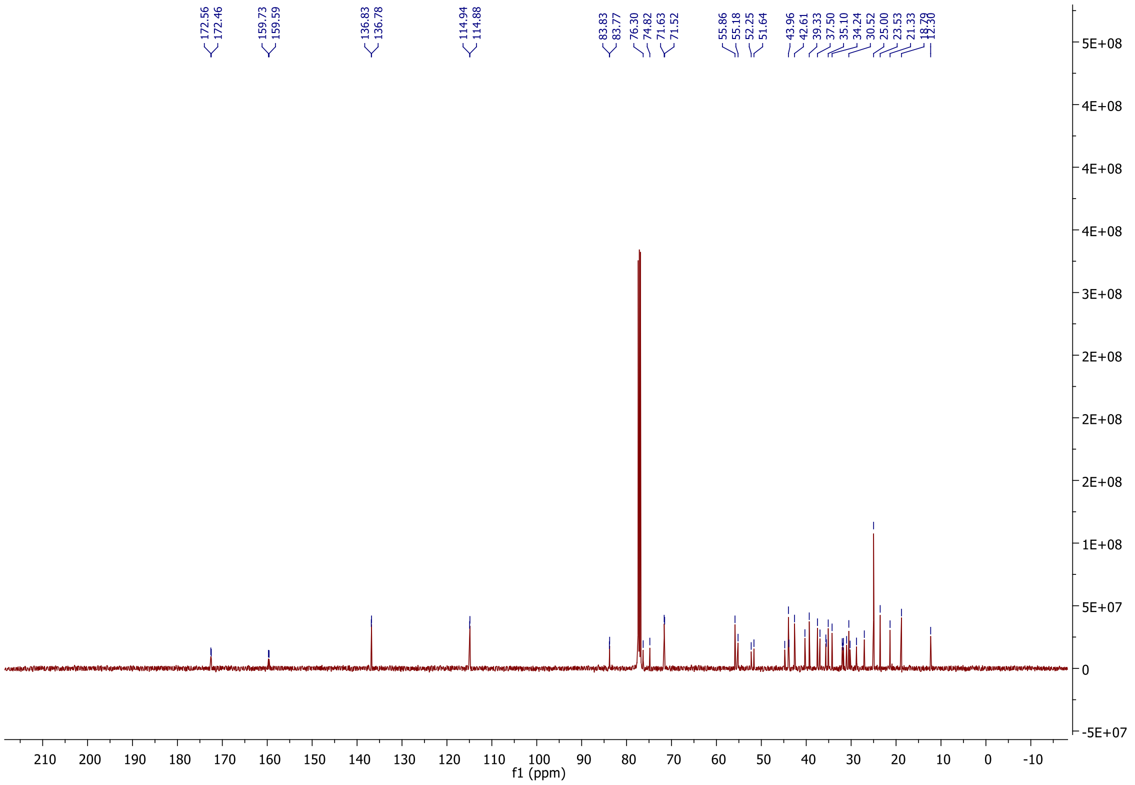

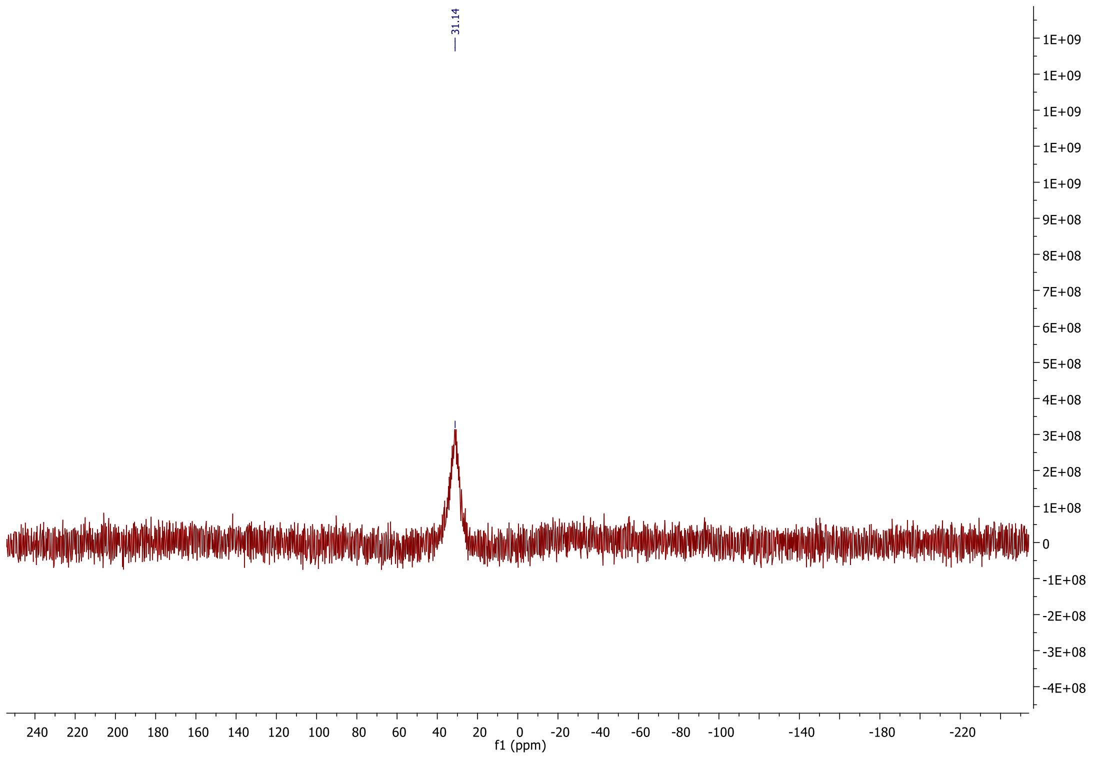

31.14
f1 (ppm)

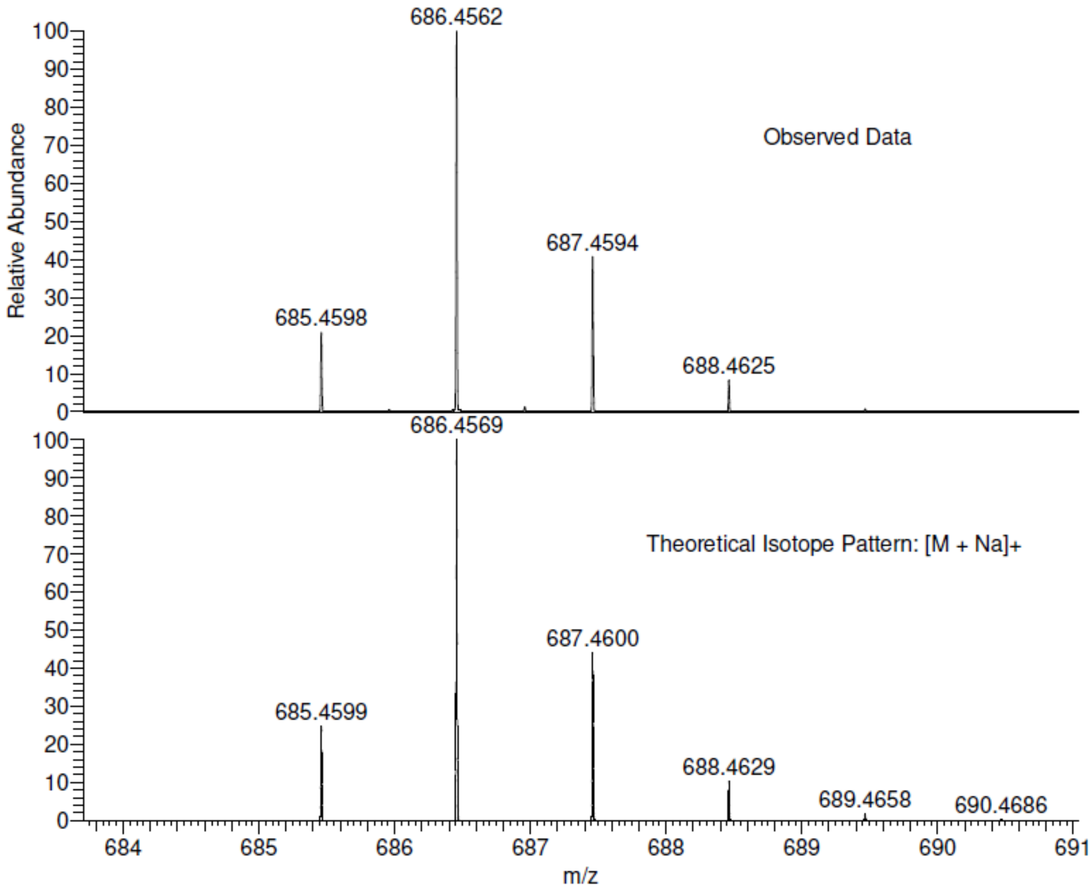

686.4562
Observed Data
687.4594
685.4598
688.4625
686.4569
Theoretical Isotope Pattern: [M + Na]+
687.4600
685.4599
688.4629
689.4658
690.4686
Relative Abundance
m/z

Spectra for **22**

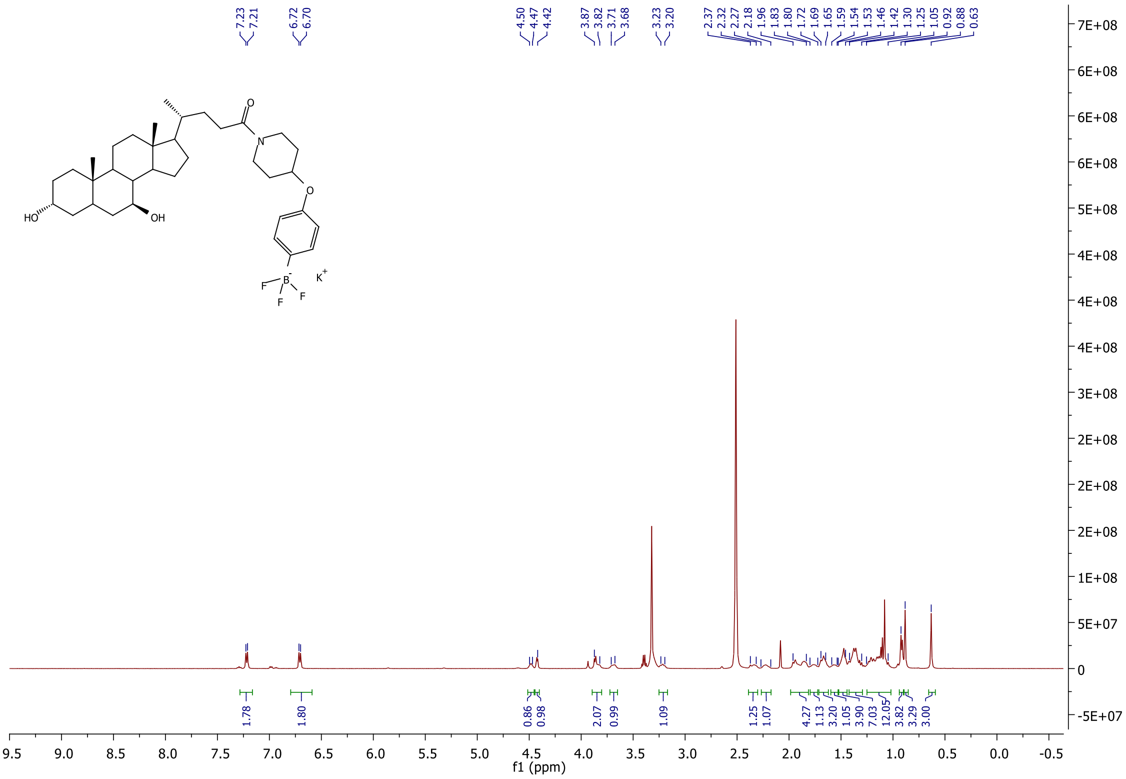

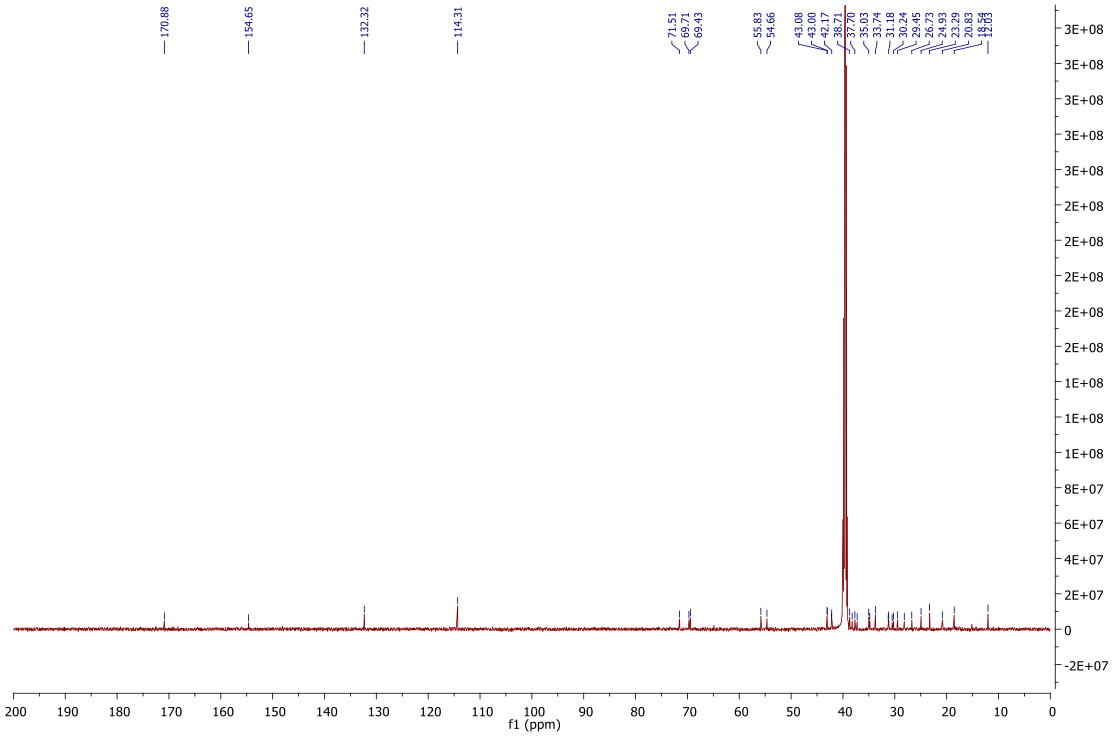

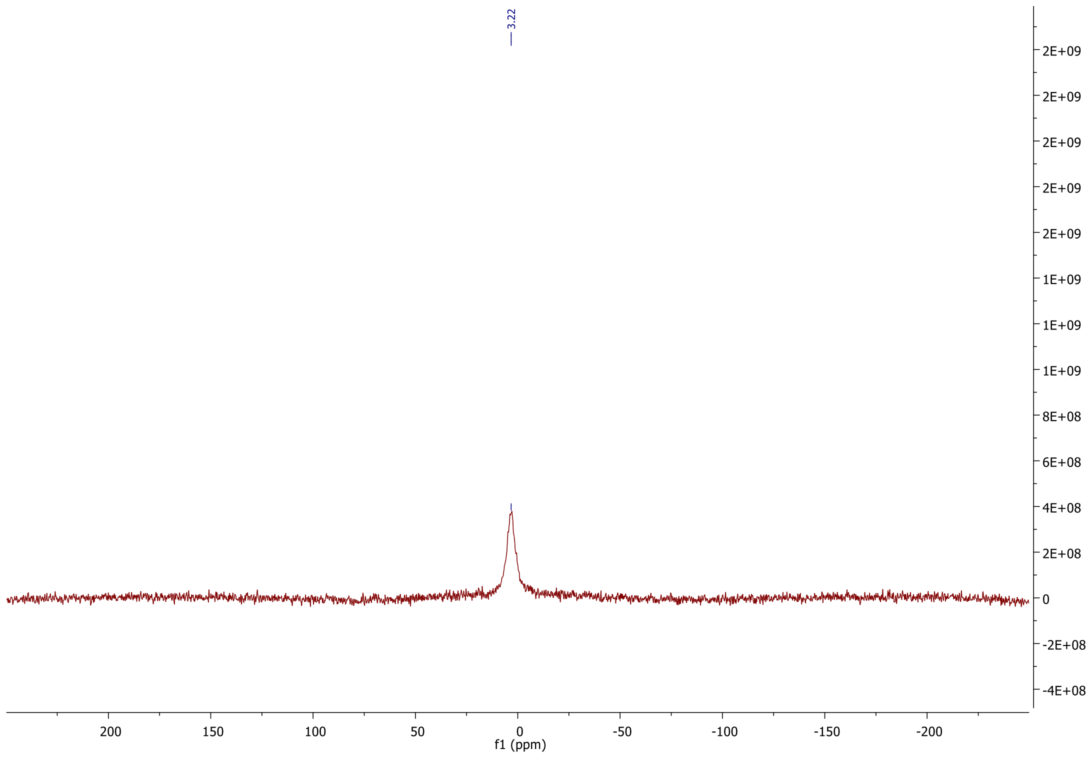
3.22
2E+09
2E+09
2E+09
2E+09
2E+09
1E+09
1E+09
1E+09
8E+08
6E+08
4E+08
2E+08
0
-2E+08
-4E+08
200
150
100
50
0
-50
-100
-150
-200
f1 (ppm)

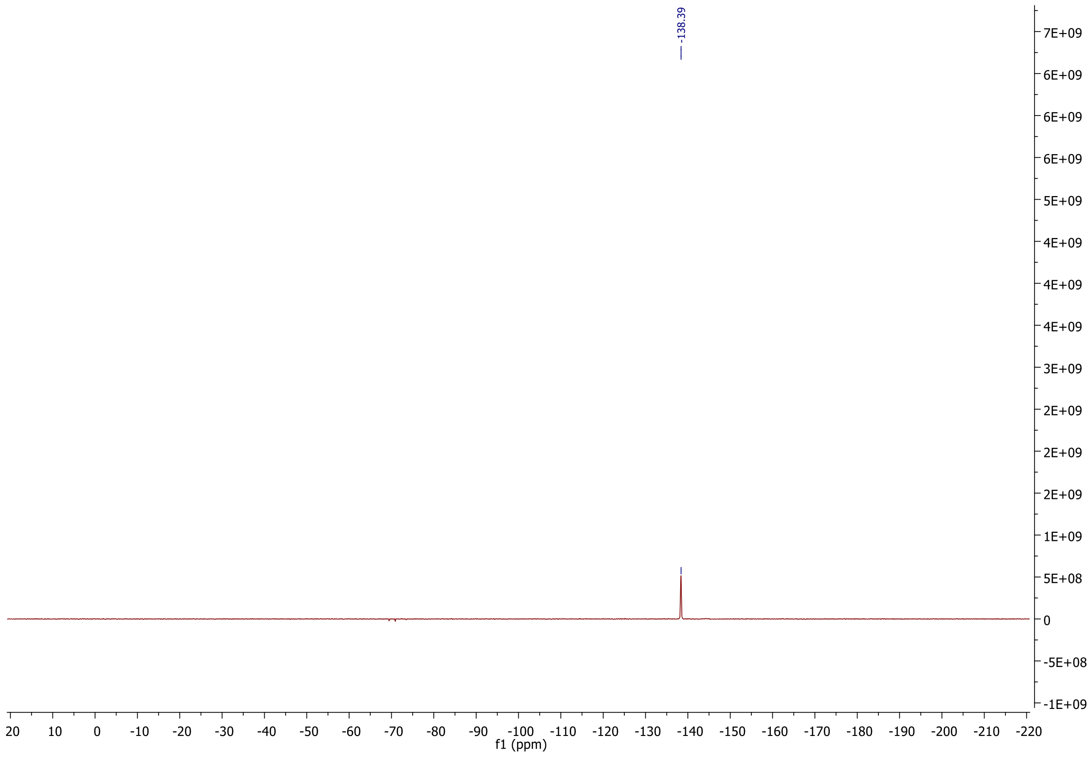
-138.39
7E+09
6E+09
6E+09
6E+09
5E+09
4E+09
4E+09
4E+09
3E+09
2E+09
2E+09
2E+09
1E+09
5E+08
0
-5E+08
-1E+09
20 10 0 -10 -20 -30 -40 -50 -60 -70 -80 -90 -100 -110 -120 -130 -140 -150 -160 -170 -180 -190 -200 -210 -220
f1 (ppm)

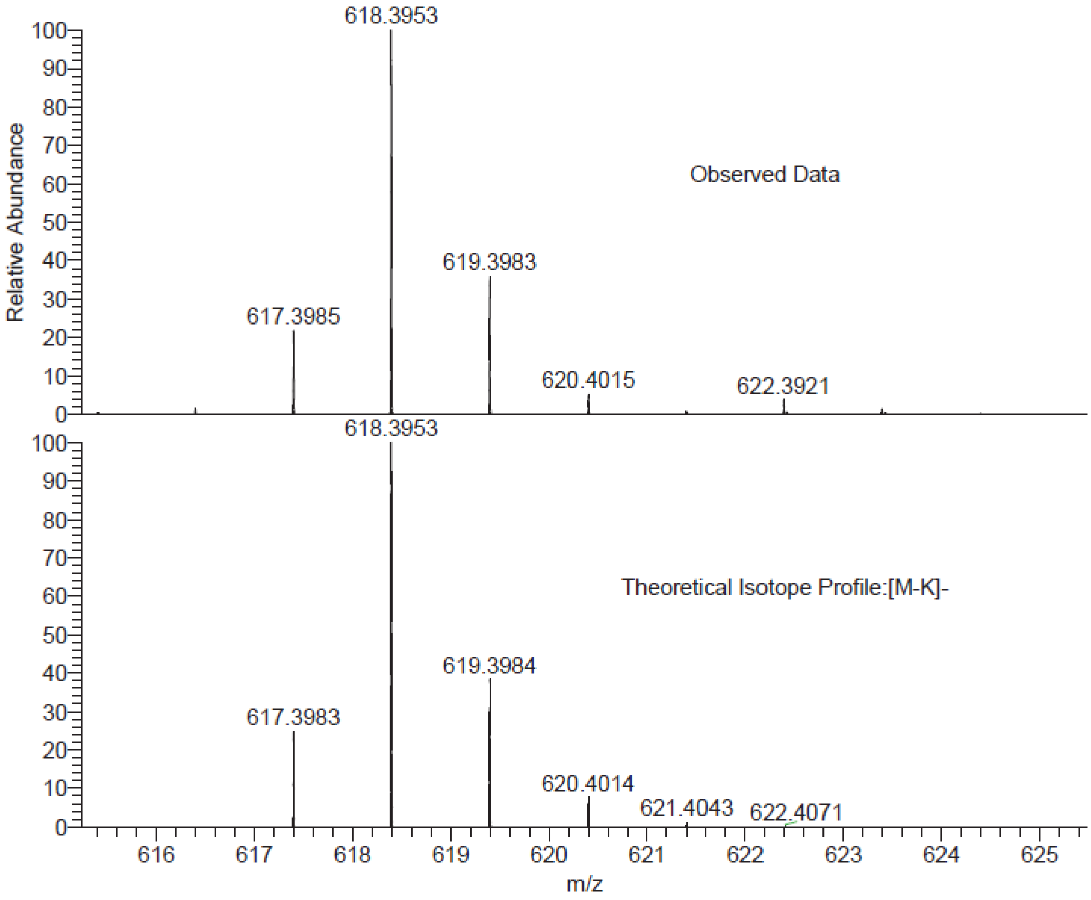

618.3953
Relative Abundance
Observed Data
619.3983
617.3985
620.4015
622.3921
618.3953
Theoretical Isotope Profile:[M-K]-
619.3984
617.3983
620.4014
621.4043
622.4071
m/z

Spectra for **23**

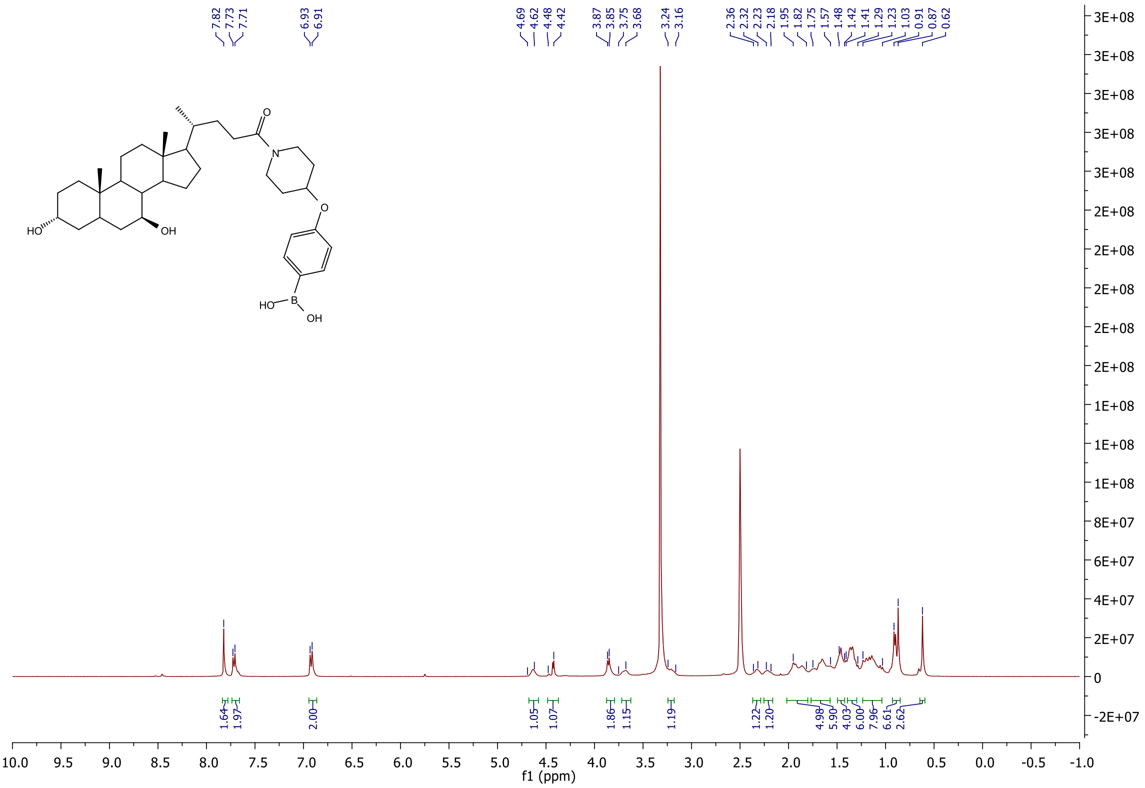

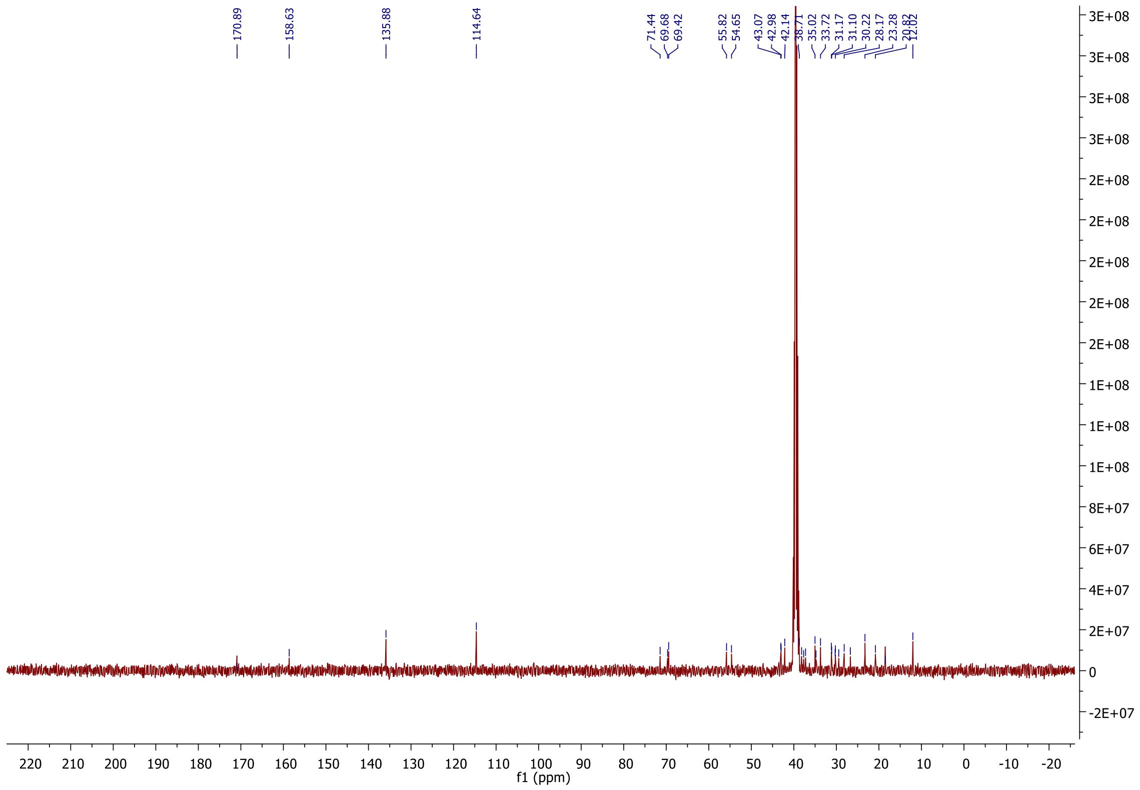

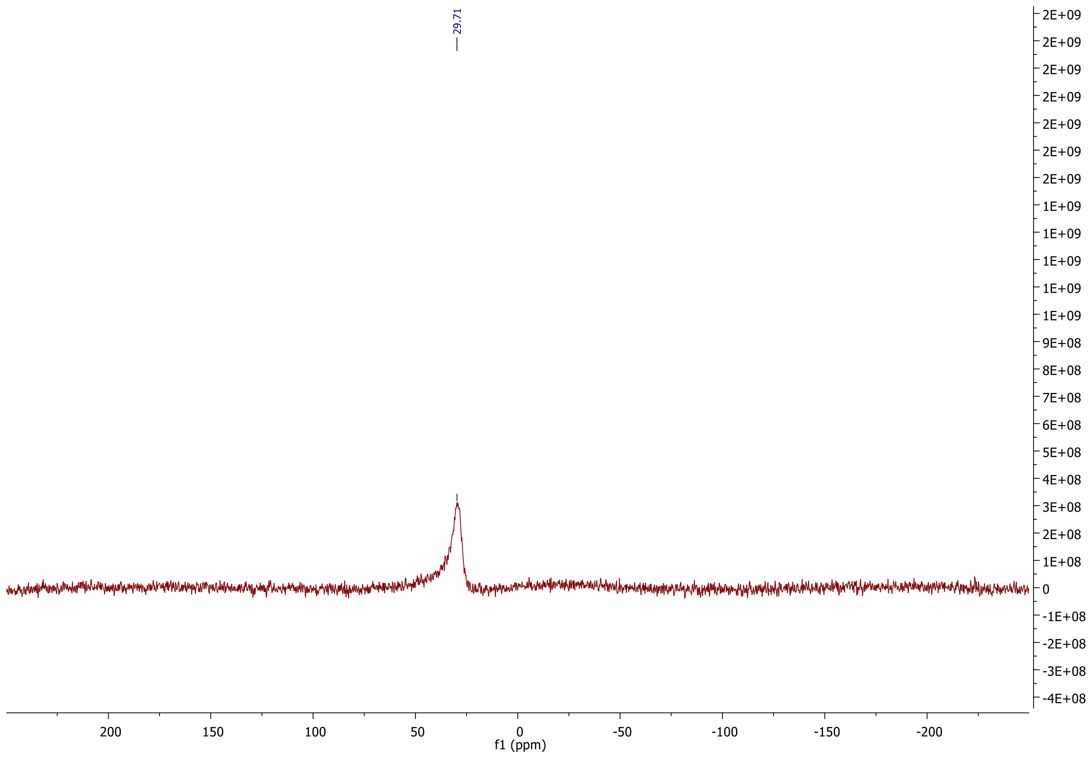
29.71
f1 (ppm)

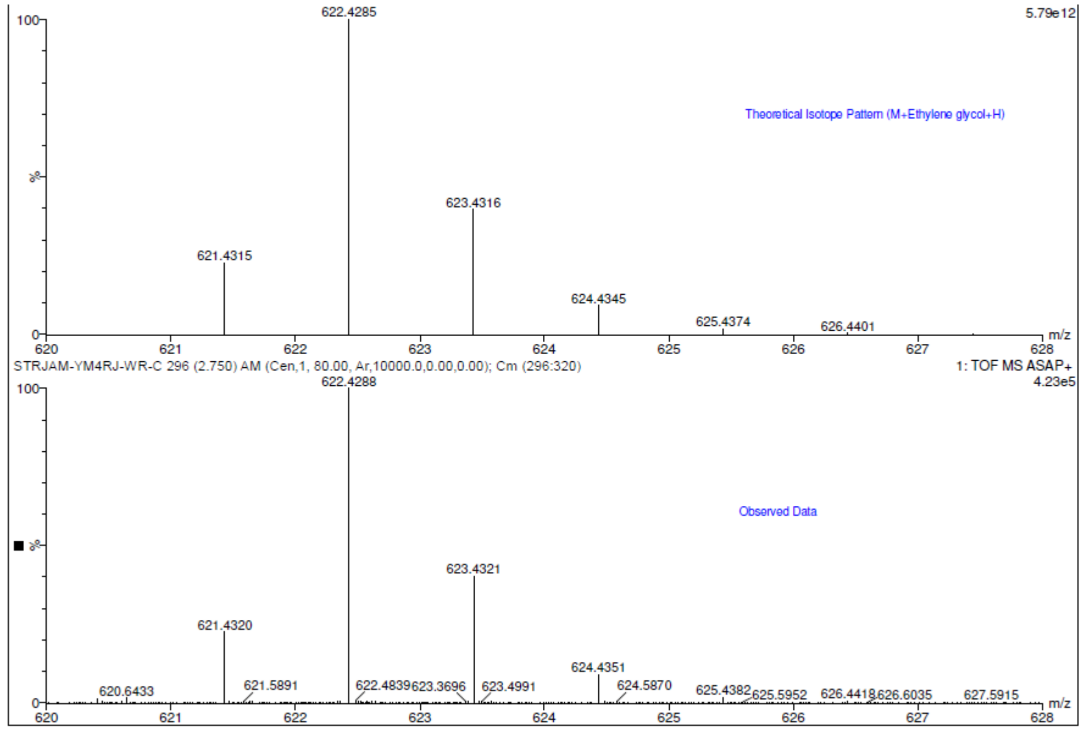
Theoretical Isotope Pattern (M+Ethylene glycol+H)
622.4285
623.4316
621.4315
624.4345
625.4374
626.4401
5.79e12
STRJAM-YM4RJ-WR-C 296 (2.750) AM (Cen,1, 80.00, Ar,10000.0,0.00,0.00); Cm (296:320)
1: TOF MS ASAP+
4.23e5
Observed Data
622.4288
623.4321
621.4320
624.4351
620.6433
621.5891
622.4839
623.3696
623.4991
624.5870
625.4382
625.5952
626.4418
626.6035
627.5915
m/z

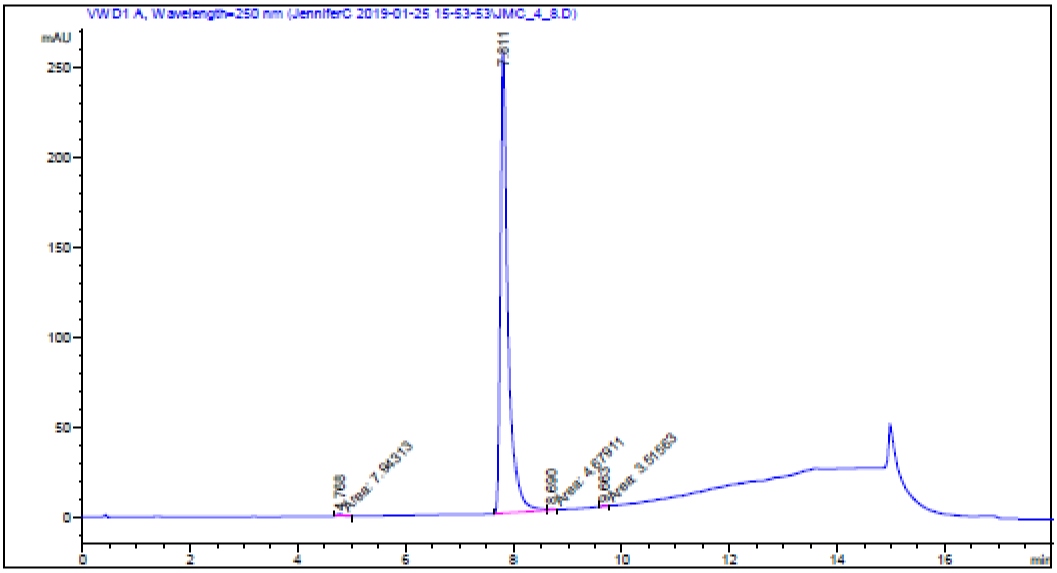

==

Area Percent Report

==

Sorted By : Signal
Multiplier : 1.0000
Dilution : 1.0000
Use Multiplier & Dilution Factor with ISTDs

Signal 1: VWD1 A, Wavelength=250 nm

Peak #	RetTime [min]	Type	Width [min]	Area [mAU*s]	Height [mAU]	Area %
1	4.768	MM	0.1064	7.94313	1.24405	0.3166
2	7.811	BV	0.1396	2492.83911	256.41290	99.3568
3	8.690	MM	0.1307	4.67911	5.96480e-1	0.1865
4	9.663	MM	0.1012	3.51563	5.78959e-1	0.1401

Totals : 2508.97698 258.83239

==

*** End of Report ***

5. References

1. M. Bozdag, M. Pinard, F. Carta, E. Masini, A. Scozzafava, R. McKenna, C. T. Supuran. A Class of 4-Sulfamoylphenyl-ω-aminoalkyl Ethers with Effective Carbonic Anhydrase Inhibitory Action and Antiglaucoma Effects. *J. Med. Chem.* **2014**, *57*, 9673–9686.

2. A. J. J. Lennox, G. C. Lloyd-Jones. Preparation of Organotrifluoroborate Salts: Precipitation-Driven Equilibrium under Non-Etching Conditions. *Angew. Chemie Int. Ed.* **2012**, *51*, 9385–9388.
